# Characteristics of *Amorphophallus konjac* as indicated by its genome

**DOI:** 10.1038/s41598-023-49963-9

**Published:** 2023-12-19

**Authors:** Lifang Li, Min Yang, Wei Wei, Jianrong Zhao, Xuya Yu, Rarisara Impaprasert, Jianguang Wang, Jiani Liu, Feiyan Huang, George Srzednicki, Lei Yu

**Affiliations:** 1 College of Agronomy, Yunnan Urban Agricultural Engineering and Technological Research Center, Kunming University, Kunming, China; 2 Faculty of Life Science and Technology, Kunming University of Science and Technology, Kunming, China; 3 Department of Microbiology, King Mongkut’s University of Technology Thonburi, Bangkok, Thailand; 4 School of Life Sciences, Yunnan University, Kunming, China; 5 Food Science & Technology, School of Chemical Engineering, The University of New South Wales, Sydney, Australia

**Keywords:** Plant breeding, Plant genetics, Plant molecular biology, Agricultural genetics, Genomics, Plant breeding, Plant genetics

## Abstract

*Amorphophallus konjac*, belonging to the genus *Amorphophallus* of the Araceae family, is an economically important crop widely used in health products and biomaterials. In the present work, we performed the whole-genome assembly of *A. konjac* based on the NovaSeq platform sequence data. The final genome assembly was 4.58 Gb with a scaffold N50 of 3212 bp. The genome includes 39,421 protein-coding genes, and 71.75% of the assemblies were repetitive sequences. Comparative genomic analysis showed 1647 gene families have expanded and 2685 contracted in the *A. konjac* genome. Likewise, genome evolution analysis indicated that *A. konjac* underwent whole-genome duplication, possibly contributing to the expansion of certain gene families. Furthermore, we identified many candidate genes involved in the tuber formation and development, cellulose and lignification synthesis. The genome of *A. konjac* obtained in this work provides a valuable resource for the further study of the genetics, genomics, and breeding of this economically important crop, as well as for evolutionary studies of Araceae family.

## Introduction

**Figure 1:**
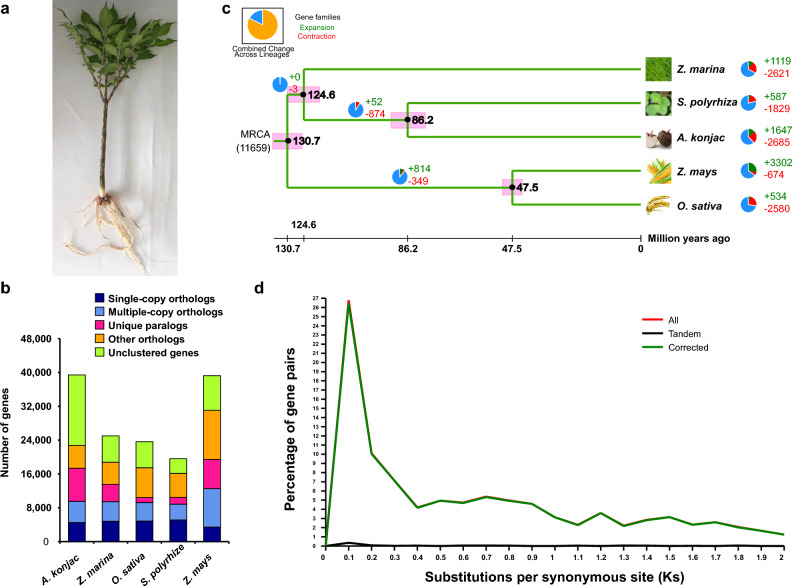
Overview for evolutionary analysis of *A. konjac*. (**a**) Images of the sequenced *A. konjac*. (**b**) Ortholog clustering analysis of the protein-coding genes in the *A. konjac* genome. (**c**) Phylogenetic tree and divergence time of *A. konjac* and four plant species. Phylogenetic tree was generated from the single-copy orthologs using the maximum-likelihood method. The divergence time range is shown by red blocks. The predicted divergence time is shown as number inside the pink blocks. The pie charts show the proportion of expanded/contracted gene families in each plant species. (**d**) Distribution of substitutions per synonymous site (Ks) in *A. konjac*.

The genus *Amorphophallus*^1^, a member of the Araceae family, is a perennial, herbaceous plant (Fig. 1a). It is estimated that it includes over 170 species occurring from West Africa, through subtropical and tropical Asia and further south in the tropical regions of the western Pacific and north-eastern Australia^2^. The *Amorphophallus* plants store their reserve polysaccharides, starch and glucomannan, in underground tubers. Some of these species contain considerable amounts of konjac glucomannan (KGM). The species producing glucomannan are generally known by the common name ‘konjac’ and are economically important as a raw material for food and pharmaceutical products worldwide^3^. This common name comes from *Amorphophallus konjac*, species that has been used widely in China and Japan for commercial KGM production. KGM it is used in products ranging from emulsifiers to weight loss supplements, in addition to its long-standing usage as a food and traditional medicine. China is both, a center of diversity for *Amorphophallus* and one of the major producers of this plant worldwide. It is also, along with Japan, one of the leading producers of KGM derived products. *A. konjac* is a diploid species (2n = 13) and is one of the important commercial crops cultivated in the central and western regions of China because it is the only plant species which is rich in KGM concentration^4^. KGM is a water-soluble, neutral polysaccharide with a high molecular weight^5,6^. KGM is a β-1, 4 linked polysaccharide composed of a d-glucose (G) and d-mannoses (M) backbone^7^. The KGM backbone possesses 5–10% acetyl-substituted residues and the presence of substituted group benefits KGM for the solubility in aqueous solution, leading to high viscosity that forms a thick hydrocolloid even when used at low concentrations^8^. This property makes it one of the most versatile and economically useful hydrocolloids with industrial applications including the manufacture of foods, pharmaceuticals and chemicals. KGM is used in a wide range of commercial products throughout Asia and increasingly throughout the rest of the world^6^. Thus, the high quality and purity of KGM obtained from *A. konjac* make it the most abundant cultivated *Amophophallus* species in China, especially in Yunnan. Daguan county is one of the largest plantation areas of *A. konjac* in Yunnan and the local *A. konjac* as an economically important crop for rural revitalization in this region. Then, the representative landrace *A. konjac* in this region was used for whole genome sequencing.

Given the economic potential of KGM, a number of studies have been conducted on *Amorphophallus* species producing this biopolymer^6,9–11^. The researchers focused on the relationship between genetic markers and KGM biosynthesis in *A. konjac*, and adopted a transcriptomics approach to identify potentially useful regions in the genome. They also studied several other KGM producing *Amorphophallus* species. These studies are on-going in order to better understand the association between genetic diversity and KGM content in a broader population of *Amorphophallus* species.

The main species of *Amorphophallus* genus have been studied and described in relation to their morphology and palynology^12–15^. Since the morphological and palynological characters are highly variable, a number of molecular markers have been employed to determine relationships in the genus. These markers include the *LEAFY* (*FLint2*) gene and the chloroplast regions *rbcL*, *matK* and *trnL*^16–19^. Since phylogenetic studies based on these regions do not produce consistent cladograms (due to a high level of conflicting signals in the informative characters), further variable regions and also other non-sequencing molecular methods are needed to establish the evolutionary history of *Amorphophallus*. The transcriptomics approach may lead to useful insights into important traits such as KGM production, tuber formation and development and other characteristics.

The genomes of two important monocotyledonous species in the order of Alismatales namely *Spirodela polyrhiza*^20^ and *Zostera marina*^21^ have been sequenced and their characteristics have been described by the authors of these papers*.* Although *A. konjac* as a glucomannan-producing cash crop in many Asian countries, there have been no any genomic information reports on *A. konjac* before we conducted whole-genome sequencing on this species. Therefore, we sequenced the whole genome of *A. konjac*, and the data was submitted to the NCBI database in 2020. Although Gao et al. subsequently provided a high-quality chromosome-level genome of *A. konjac*^22^, our results can also enrich the genomic information of *Amorphophallus* to a certain extent. In this study, we performed a series of genomic analyses on *A. konjac* including assembly, annotations, identification of phylogenetic relationship, gene family analysis, divergence time estimation. We also identified cellulose and lignification synthesis genes, and tuber formation and development genes. The results will provide important insights as well as resources for future study of *A. konjac*.

## Results

### Genome assembly and annotation

The DNA sequencing data (1119.58 Gb, average 110× coverage) of the *A. konjac* sample were obtained using the Illumina Hiseq 2500 sequencer. A summary of the sequence data used for the assembly is presented in Table S1. The estimated genome size is 4,512,012,462 bp using 19-mer frequency distribution based on the paired-end sequenceing data (Fig. S1), which is consistent with measurement by flow cytometry (Fig. S2). Based on the Illumina sequencing data, 2.99 Gb contigs were assembled using SOAPdenovo2^23^ (Table S2). After constructing scaffolds and filling gaps, the 4.58 Gb *A. konja*c reference genome was assembled, and this resulted in the 7,423,768 scaffolds with a scaffold N50 of 3212 bp (Tables 1, S2). The *A. konjac* genome shows significant genomic synteny with *S. polyrhiza*. The assembly performed in this study captured 75.81% (188 of 248) of core eukaryotic genes (Table S3) and captured 624 complete BUSCOs v5.2.2 (Table S4) using core eukaryotic genes mapping approach software (CEGMA) and BUSCO software^24^, respectively.

**Table 1:** Summary of genome assembly and annotation.

Assembly	
Assembled genome size (bp)	4,584,988,971
Genome-sequencing depth (×)	244.18
No. of scaffolds	7,423,768
N50 of scaffolds (bp)	3212
Longest scaffold (bp)	85,347
GC content of the genome (%)	45.71
N length (bp)	887,681,325
Annotation	
Percentage of repeat sequences (%)	71.75
Repeat sequence length (bp)	3,289,511,160
No. of predicted protein-coding genes	39,241
Percentage of average gene length (bp)	1,372.75
Average exon length (bp)	257.08
Average exon per gene	2.29
Total intron length (bp)	30,870,726
tRNAs	761
rRNAs	2894
snRNAs	1553
miRNAs	1078
Family number	13,190
Genes in families	22,730
Average genes per family	1.72
Unique families	3001
Un-clustered genes	16,691

Combination of de novo prediction and homology-based search resulted in identification of 3,289,511,160 bp repetitive elements in *A. konjac* genome (Table S5), make up about 71.75% of the assembled genomes (Table S5). Most of the repeats were de novo predicted (70.98%), the repeats detected by homologous method were relatively few (Table S5). Among the repeats in the *A. konjac* genome, 69.16% were transposable elements (TEs), of which 52.06% were long terminal repeats (LTR), including 31.42% Gypsy LTRs and 11.6% Copia LTRs (Table S6).

A total of 39,241 protein-coding genes were predicted in assembled genomes following a combination of homology and ab initio methods, with an average coding length of 1372.75 bp and a mean of 2.29 exons per gene, respectively (Table 1, Fig. S3, Table S7), the gene number and average gene length is close to that of *S. polyrhiza* and the average gene is longer than that of *Oryza sativa* and *Zea mays* (Fig. S4, Table S7). Moreover, an average of 92.22% of the RNA sequencing (RNA-seq) reads of the four *A. konjac* tissues (leaf, stem, root and tuber) could be mapped to the genome. In addition, 65.26% of the predicted genes (25,725/39,241) showed expression levels (FPKM > 0.05) by aligning leaf, stem, root and tuber RNA-seq data to the set of protein-coding genes using TopHat2^25^, and estimating expression values based on the resulting alignments using Cufflinks^26^. In total, 26,456, 26,512, 25,797 and 33,715 of the predicted genes were assigned with a functional annotation in the Swiss-Prot, KEGG, InterProScan, and Trembl databases, respectively (Table S8), a total of 34,126 of the predicted genes (87%) were assigned with a functional annotation in at least one database (Table S8).

An overview of annotated ncRNA is shown in Table S9. 1078 miRNAs, 761 tRNAs, 2894 rRNAs and 1553 snRNAs were predicted in *A. konjac*.

### Gene family cluster

Based on pair-wise protein sequence similarity, the gene family clustering analysis of five species genes, *Z. marina, O. sativa, S. polyrhiza, Z. mays* and *A. konjac* has been carried out. A total of 22,730 genes in *A. konjac* were clustered into 13,190 gene families, however, *A. konjac* has 16,691 unclustered genes and 3001 unique gene families (Table 1, Fig. 1b, Fig. S5A, Table S10), that is more than other four species, and the number of single-copy orthologs genes in *A. konjac* is 4509. The Venn diagram (Fig. S5a) shows that five species share a common core set of 6438 gene families.

The unique gene families in *A. konjac* were enriched in nucleobase-containing compound biosynthetic process, nucleobase-containing compound catabolic process, regulation of nucleobase-containing compound metabolic process, aromatic compound biosynthetic process, heterocycle catabolic process, negative regulation of growth, 1,3-beta-d-glucan synthase complex, cytoskeleton organization, membrane, molecular function regulator, peptidase regulator activity, 1,3-beta-d-glucan synthase activity and so on (Fig. S5B). Moreover, the unique gene families containa large number of unique paralogous genes (7847 genes) that are not orthologous to any known genes in other four species, which were enriched in 1,3-beta-d-glucan synthase complex, a series of related components of vesicle membrane and so on in cellular component. The 1,3-beta-d-glucan synthase complex can catalyse the transfer of a glucose group from UDP-glucose to a (1→3)-beta-d-glucan chain, which may be related with the high starch content in tuber and the fast-growing trait in *A. konjac*.

### Evolution, expansion and contraction

To systematically study the evolutionary dynamics of Alismatales species, species phylogeny was performed utilizing single-copy orthologous genes among five species, which included 4509 single-copy orthologous genes in *A. konjac*. As illustrated in Fig. 1c, the estimated divergence time is 130.7 (124.6–139.9) million years ago (MYA) between Alismatales and Poaceae, Araceae and Zosteraceae separated at about 124.6 (115.3–131.9) MYA, the divergence time is 86.2 (78.2–96.0) MYA between *S. polyrhiza* and *A. konjac* (Fig. 1c). This result based on genomic data will provide a phylogenetic framework for interpreting the evolutionary events of the family.

Comparative analysis of the gene family expansion and contraction showed that 1647 gene families have expanded and 2685 contracted in the *A. konjac* genome (Fig. 1c). Based on the InterProScan functional annotation, the expansive genes in *A. konjac* were enriched in iron coordination entity transport, vitamin E metabolic process, vitamin E biosynthetic process, cofactor transport, heme transport and so on in the biochemical processes (p-value < 0.05) (Fig. S6). Furthermore, the gene families that had undergone contraction in *A. konjac* were enriched in reproduction, pollination, pollen-pistil interaction, multi-sprout formation, reproductive process, cell recognition and various biochemical processes (p-value < 0.05) (Fig. S7), which may suggest that the mode of reproduction is asexual reproduction principally in *A. konjac*, and the occurrence of sexual reproduction needs particular conditions.

Whole-genome duplication (WGD) followed by gene loss has been found in most eudicots and is regarded as the major evolutionary force that gives rise to gene neofunctionalisation in both plants and animals^27^. Synonymous substitution rates showed a unimodal distribution, indicating that the WGD of *A. konjac* occurred recently (Fig. 1d), it needs better reference genome to identify that whether or not it corresponds to the ⍺SP/βSP WGDs in Alismatales^20^.

### Detection of positively selected genes

Positive selection was proposed to contribute to fitness. Respectively 686 and 122 genes of *A. konjac* were determined as positively selected genes and compared with *S. polyrhiza* and *Z. marina* (Tables S11, S12). GO enrichments showed that more positively selected genes in *A. konjac* in comparison with *S. polyrhiza* were involved in RNA biosynthetic process, regulation of biosynthetic process, regulation of gene expression, protein modification process, cell wall organization or biogenesis, transcription, DNA-templated cell synthesis, cell growth and so on (Fig. S8). Moreover, the positively selected genes in *A. konjac* were more involved than those in *Z. marina* in leucine biosynthetic process, regulation of signal transduction, regulation of cell communication, regulation of signaling, regulation of response to stimulus and so on (Fig. S9).

### Analysis of transcription factor families

Transcription factors regulate gene expression and protein kinases regulate cellular activities by phosphorylating target proteins in response to internal or external signals. This study identified a total of 1275 transcription factors and 345 transcriptional regulators in *A. konjac* (Table S13). The number of transcription factors in *A. konjac* is more than in *S. polyrhiza* (1115 genes)*,* and the number of transcriptional regulators in *A. konjac* is more than in both, *S. polyrhiza* and *Z. marina* (271 and 307 genes, respectively), but fewer than that in maize (573 genes)*.* The *AP2/ERF-ERF*, *GRAS*, *HSF*, *SBP*, *ULT* transcription factors are more abundant in *A. konjac* in comparison with *S. polyrhiza* and *Z. marina*, as well as the *AUX/IAA*, *mTERF*, and *SNF2* transcriptional regulators. This difference may be caused by different growth environment, *A. konjac* is a terrestrial plant, while other two are hydrophilous plants. In addition, the number of *BBR-BPC* and *ULT* genes in *A. konjac* is higher than in maize. In co-transfection experiments, BBR activates (GA/TC)-containing promoters^27^, and its overexpression in tobacco leads to a pronounced leaf shape modification^28^. In *Arabidopsis*, the *ULTRAPETALA1* (*ULT1*) gene is a key negative regulator of cell accumulation shoot and floral meristems, and the mutations in *ULT1* can cause the enlargement of inflorescence and floral meristems, the production of supernumerary flowers and floral organs, and a delay in floral meristem termination, downregulation of both *ULT* genes can lead to shoot apical meristem arrest shortly after germination, revealing a requirement for *ULT* activity in early development^29^.

### Contractive cellulose and lignification synthesis genes

**Figure 2:**
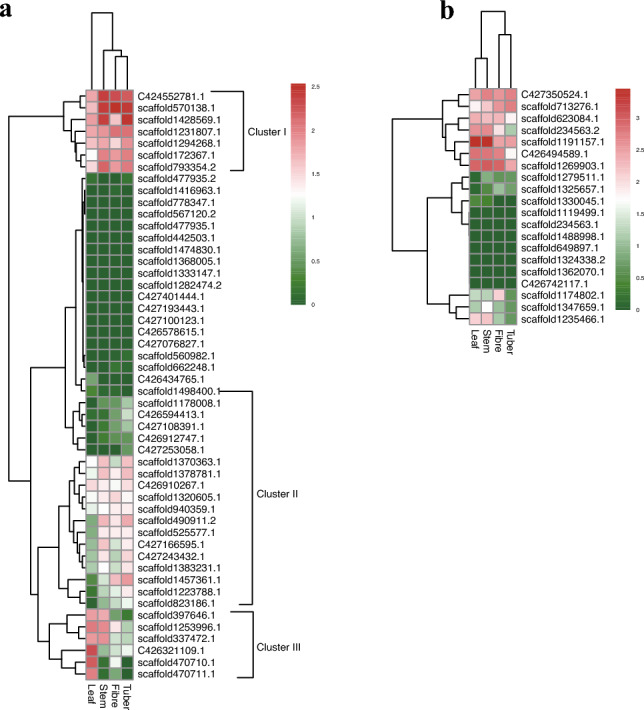
Heatmaps of gene expression. (**a**) Heatmap depicting the expressed profile of *CesA* and *Csl* genes; (**b**) Heatmap depicting the expressed profile of lignin-related genes.

*Amorphophallus konjac* is a lodging plant a trait that is consistent with a reduction of genes involved in cell wall biosynthesis and lignification. According to InterProScan annotation, 50 cellulose synthase (*CesA*) and cellulose synthase-like (*Csl*) genes were identified in *A. konjac* (Table 2), which is obviously fewer than in the woody bamboo species. Lignin, a major component of secondary cell wall, plays an important role for support, water transport and stress responses in vascular plants^19^. A total of 20 genes involved in the lignin biosynthesis pathway were detected in *A. konjac* (Table 2), which contained 6 lignin biosynthesis gene families out of 10 families (*PAL*, *4CL*, *HCT*, *CCR*, *F5H*, *CAD* but not *C4H*, *C3H*, *CCoAMT*, *COMT*). Overall, the absolute copy number of both cellulose- and lignin-related genes decreased in *A. konjac* compared with woody species. The expression of *CesA* and *Csl* genes also showed two different profiles (Fig. 2a), of which the expression of most genes (Cluster I and Cluster II) was higher in tuber, fibre and stem than in leaf, and expression of six genes (cluster III) were higher in leaf than in tuber, fibre and stem. For the expressed profile of lignin-related genes, the leaf and stem showed distinct difference against fibre and tuber (Fig. 2b).

**Table 2:** Copy number variations of cellulose synthase (CesA), cellulose synthase-like (Csl), and lignification synthesis related genes between 12 plants.

	Akon	Bam^a^	Ped^a^	Ola^a^	Rgu^a^	Bdi^a^	Osa^a^	Zma^a^	Sbi^a^	Ath^a^	Ptr^a^	Spir^b^
CesA	15	27	26	12	10	19	11	20	12	10	18	10
Csl	35	55	51	40	35	24	34	33	37	29	37	21
PAL	3	13	8	6	7	8	9	10	9	4	5	3
C4H	0	6	4	1	2	3	4	4	3	1	2	3
4CL	7	11	6	4	5	5	5	3	5	4	5	9
HCT	1	5	4	2	2	2	2	2	2	1	2	20
C3H	0	3	3	1	2	1	2	2	2	3	3	1
CCoAOMT	0	2	2	2	1	1	1	2	1	1	2	1
CCR	3	7	5	5	2	2	2	1	2	2	7	21
F5H	4	3	3	2	2	2	3	2	2	2	4	3
COMT	0	2	1	2	1	1	1	1	1	1	2	5
CAD	2	2	3	5	1	1	1	1	1	2	1	4

PAL: Phenylalanine ammonia lyase; C4H: Cinnamate-4-hydroxylase; C3H: ρ-Coumaroyl 3′-hydroxylase/Coumaroyl 3-hydroxylase; 4CL: 4-Coumarate CoA Ligas; HCT: Hydroxycinnamoyl-CoA: shikimate/quinate hydroxycinnamoyltransferase; CCR: Cinnamoyl-CoA reductase; CCoAOMT: Trans-caffeoyl-CoA 3-O-methyltransferase; CAD: Cinnamyl alcohol dehydrogenase; F5H: Ferulate 5-hydroxylase; COMT: Caffeic acid 3-O-methyltransferase.

Akon: *Amorphophallus konjac*; Bam: *Bonia amplexicaulis*; Ped: *Phyllostachys edulis*; Ola: *Olyra latifolia*; Rgu: *Raddia guianensis*; Bdi: *Brachypodium distachyon*; Osa: *Oryza sativa*; Zma: *Zea mays*; Sbi: *Sorghum bicolor*; Ath: *Arabidopsis thaliana*; Ptr: *Populus trichocarpa*; Spir: *Spirodela polyrhiza.*

^a^Data from Guo et al.^70^.

^b^data from Wang et al.^20^.

### Tuber formation and development genes

**Figure 3:**
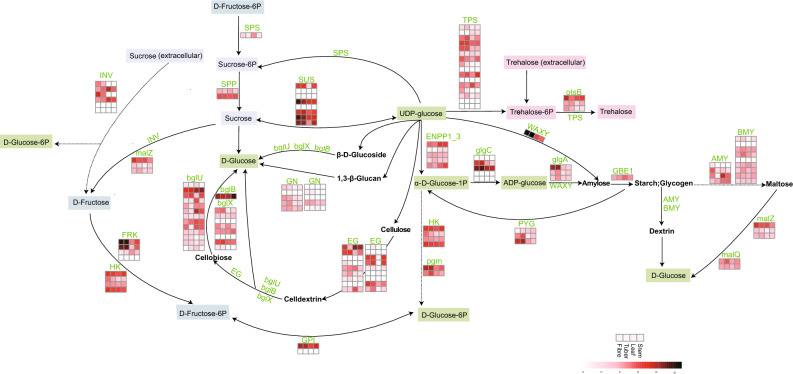
The expression profiles in FPKM (fragments per kilobase per million reads mapped) of genes involved in the starch and sucrose metabolism pathway in the four tissues (tuber, fibre, stem and leaf) from 7-month-old plant of *A. konjac*. Data are plotted as log10 values. PYG: glycogen phosphorylase; SUS: sucrose synthase; GBE1: 1,4-alpha-glucan branching enzyme; glgA: starch synthase; malQ: 4-alpha-glucanotransferase; HK: hexokinase; FRK: fructokinase; glgC: glucose-1-phosphate adenylyltransferase; otsB: trehalose 6-phosphate phosphatase; AMY: alpha-amylase; BMY: beta-amylase; EG: endoglucanase; malZ: alpha-glucosidase; bglU: beta-glucosidase; INV: beta-fructofuranosidase; ENPP1_3: ectonucleotide pyrophosphatase/phosphodiesterase family member 1/3; GPI: glucose-6-phosphate isomerase; pgm: phosphoglucomutase; bglX: beta-glucosidase; bglB: beta-glucosidase; SPP: sucrose-6-phosphatase; WAXY: granule-bound starch synthase; TPS: trehalose 6-phosphate synthase/phosphatase; GN: included GN1_2_3 (glucan endo-1,3-beta-glucosidase 1/2/3), GN4 (glucan endo-1,3-beta-glucosidase 4) and GN5_6 (glucan endo-1,3-beta-glucosidase 5/6).

Sucrose metabolism is considered important for the development of a plant sink organ. In most plants, assimilated carbon in source leaves is transported as sucrose into sink organs, including roots, tubers, fruit, and seeds^30^. The present study investigated the genes related to starch and sucrose metabolism pathway and found that the expressed profile of most genes in fibre and tuber showed distinct difference against the leaf and stem, which were consistently high expression (Fig. 3, Table S14). To utilise sucrose, this bond should be cleaved to generate the two monosaccharides. Sucrose synthase (SUS) is the key enzyme that catalyzes both the synthesis and the cleavage of sucrose^30^. SUS is a glycosyl transferase, which converts sucrose into UDP-glucose and fructose in the presence of uridine diphosphate (UDP). *SUS* shows consistently high expression patterns in fibre and tuber, whereas low expression was observed in leaf and stem (Fig. 3). On the other hand, SPS plays a major role in photosynthetic sucrose synthesis by catalysing the rate-limiting step of sucrose biosynthesis from UDP-glucose and fructose-6-phosphate. The expression of sucrose-phosphate synthase (*SPS*) gene was higher in leaf (Fig. 3), which was consistent with the role played as a limiting factor in the export of photoassimilates out of the leaf. These results suggest that sucrose synthase specifically facilitates the storage and maturation of sinks.

**Figure 4:**
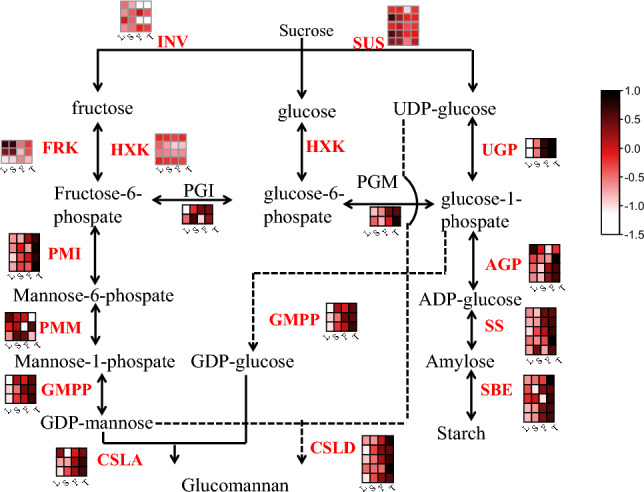
Putative biosynthetic pathway of KGM. SUS: Sucrose synthase, INV: invertase, PGI: phosphoglucose isomerase, PGM: phosphoglucomutase\, PMI: phosphomannose isomerase, PMM: phosphomannomutase, SS: starch synthase, GMPP: GDP-mannose pyrophosphorylase, UGP: UDP-glucose pyrophosphorylase, AGP: ADP-glucose pyrophosphorylase, FRK: fructokinase, HXK: hexokinase, SBE: starch branching enzyme, CSLA: cellulose synthase-like A, CSLD: Cellulose synthase-like D. L: Leaf, S: Stem, F: Fibre, T: Tuber.

Sucrose generated from photosynthates in source organs is transported to sink organs and is then converted into starch. Plants store sugar as polymerised starch, enabling the storage of a larger amount of sugar without problems caused by osmotic pressure^30^. In *A. konjac*, the starch synthase (*glgA*), granule-bound starch synthase (*WAXY*), and glucose-1-phosphate adenylyltransferase (*glgC*) showed high expression patterns in fibre and tuber (Fig. 3), which catalyse precursor substances to synthesise starch. Specially, the expression of 1,4-alpha-glucan branching enzyme (*GBE1*) gene was slightly higher in leaf when comparing the three tissues. GBE catalyzes the formation of α-1,6 branching points in starch and plays a key role in synthesis^31^. In general, starch synthesized and accumulated directly from the products of photosynthesis in the leaf during the daytime, and is then degraded into sugars as an energy source for the following night^32^. Therefore, the high expression of *GBE1* in leaf may be related to the synthesis of starch through photosynthesis.In addition, 59 putative genes involved in the pathway wrere identified (Fig. 4) according the previous studies on glucomannan biosynthesis^22,33^, and most of them also were highly expressed in fibre and tubers.

## Discussion

As a major provider of KGM, *A. konjac* is abundant in southern China and Japan. The different species of genus *Amorphophallus* show high genetic diversity. *A. konjac* is classified as a species with high KGM content. Its tubers contain between 40 and 70% KGM^33^. In the natural habitat, fruiting efficiency of *A. konjac* is less than 1% through sexual reproduction. Although breeding strategies for *A. konjac* comprise asexual and sexual reproduction, sexual reproduction happens on the condition of cross-pollination. Increasingly agricultural studies reported that special structure of inflorescence in *A. konjac* can facilitate the cross-pollination process and possibly increase diversity of KGM-biosynthetic gene pool. However, genomic background of many traits of *A. konjac* is little known.

Here, we report the earliest sequenced *A. konjac* genome, which was sequenced by our research team in 2018 and uploaded to the ncbi database. The genome assembly of *A. konjac* exhibited a total size of 4.58 Gb, which was smaller than the another genome of *A. konjac* (5.60 Gb) was assembled by Gao et al. using a combination of Illumina, PacBio, and Hi-C technology^22^. Meanwhile, Gao et al. also identified 80.6% of the assembled sequences as repetitive sequences, and 75.6% were transposable elements (TEs)^22^. Among the various TEs, long terminal repeats (LTRs, 74.04%), especially Gypsy (40.28%) and Copia (9.58%) type, were remarkably prevalent in the genome^22^. Nevertheless, we found that *A. konjac* genome comprised of 71.75% repeat sequences and 69.16% were TEs, including 31.42% Gypsy LTRs and 11.6% Copia LTRs. A potential reason for the smaller genome size and fewer repetitive sequences may be related to the second-generation sequencing data used in the present study. The second-generation sequencing technologies are difficult to get the large repetitive sequences and lead to incomplete assemblies^34,35^. Strong correlation between genome size and the proportion of TEs (especially LTR-Copia and LTR-Gypsy) has been reported in many studies^34,36, 37^. In addition, previous studiep also found that the *A. konjac* and the *S. polyrhiza* shared a recent WGD event, which is consistent with the results of this study^21^. This study employed the genome analysis to characterise genetic traits of *A. konjac*. The results implied that *A. konjac* possesses 3001 unique families and 4509 single-copy orthologs in a total of 13,190 identified genes in comparison with the other four species (*Z. marina*, *O. sativa*, *S. polyrhiza* and *Z. mays*). In addition, time-tree based on phylogenetic analysis showed that a more closely genetic relationship was found between *S. polyrhiza* and *A. Konjac* (divergent time, 86.2 million years) than another three species (divergent time, over 100 million years between *A. konjac* and *Z. marina, O. sativa* and *Z. mays*). Moreover, the data of this study further illustrated that some contracted genes in *A. konjac* genome are involve in pollination, pollen-pistil interaction and reproductive process, which may offer genomic hints for sexual reproduction of *A. konjac*.

Positive selection was proposed to contribute to fitness. The ratio of non-synonymous to synonymous substitutions (Ka/Ks), is widely used for the estimation of positive selection at the amino-acid site^38^. Analysis of the ratios of Ka/Ks between *Chrysanthemum morifolium* and *C. boreale* two Chrysanthemum species, indicating that 107 genes experienced positive selection, with Ka/Ks more than one, which may have been crucial for the adaptation, domestication, and speciation of *Chrysanthemum*^39^. In current study, we identified 625 and 111 genes in *A. konjac* were detected under positive selection compared to *S. polyrhiza* and *Z. marina*, respectively. Enrichment analysis suggested that those genes under positive selection are involved in biosynthetic process of RNA and other organic substances, regulatory process of biogenesis, cellular organization and cell growth. These results support the fact that diverse genes were under positive selection in *A. konjac*, which might influence the adaptation and evolution of *A. konjac*. Some genes under positive selection can be used as potential biomarkers for breeding outcrossing species. So far, asexual reproduction of tubers is widely used for breeding *A. konjac* in traditional agriculture. However, many problems are related to asexual breeding process, such as low breeding efficiency, long cultivation cycle, high risk of infectious diseases, and breeding degeneration. Genome analysis in the present study partially demonstrates evolutionary scenario of *A. konjac* undergoing artificial breeding, and helps to screen outcrossing populations with high KGM content.

Additionally, the analysis of the data collected in the present study suggested that a total of 20 genes were observed to act in biosynthetic pathways of lignin, which might help cells of *A. konjac* adapt in habitats suitable for fast-growing.

Over a few decades, purified KGM from tubers of *A. konjac,* a dietary fibre composed of hydro-colloidal polysaccharide, was used widely as food additive as well as dietary supplement in many countries. Results from nutritional studies indicated that KGM can decrease the levels of triglycerides, glucose, cholesterol, and blood pressure, and prevent many chronic diseases through wide-ranging regulation of metabolism^40^. Other studies suggested that KGM content over 50% dry matter should be used to obtain high-purity glucomannan for development of additives and supplements since high-purity glucomannan can easily form transparent and odourless gel with high viscosity. The cultivated *A. konjac* was reported to be major source of high KGM content material (KGM content over 45% dry matter). Apart from environmental factors and cultivation conditions, genetic factors are presumed to contribute to productive efficiency of high KGM content. However, it is still not clear which genes of *A. konjac* genome are involved in regulatory process of KGM biosynthesis in tubers. In this study, genomic and transcriptomic analysis has been applied to characterise the metabolic process of starch and sucrose in *A. konjac*. Previous studies have demonstrated that polysaccharide metabolism is essential both for formation of tuber sink and biosynthetic source of KGM in *A. konjac*. Transcriptomic analysis of *A. konjac* in the present study suggested that expression patterns of starch and sucrose metabolism differed between tubers and leaf or stem, and sucrose metabolism related genes maintained consistently higher expression level in tubers than in leaf and stem. For example, starch synthase (*glgA*), granule-bound starch synthase (*WAXY*), and glucose-1-phosphate adenylyltransferase (*glgC*) are more expressed in tubers and fibres than in leaf and stem. Previously, some physiological tests suggested the role of sucrose-phosphate synthase (SPS) as exporting factor of photoassimilates out ofthe leaf. Down regulation of *SPS* can specifically help *A. konjac* facilitate storage and maturation of polysaccharides in tubers. The findings in the present study partially clarify versatile functions of polysaccharide metabolism specific to tubers of *A. konjac*, and thus potentially help to study biosynthetic mechanism of formation of KGM.

## Conclusions

In this study, we sequenced, assembled, annotated, and analysed the genome of the *A. konjac*, which belongs to the genus *Amorphophallus* of the family Araceae. The 4.58 Gb *A. konjac* genome encoded 39,421 protein-coding genes and 3,289,511,160 bp repetitive sequences, accounting for 71.75% of the genome sequences. Whole-genome duplication event has been observed within the *A. konjac* genome. In addition, the sequencing of *A. konjac* genome revealed the evolution and the gene expressed difference in tuber formation and provided a genomic resource for further study of *Amorphophallus* genus. Comparative genomics analyses identified the contraction of gene families associated with reproduction and also genes related with cellulose and lignification synthesis. The knowledge of the genomic sequences may help in improvement of *A. konjac* germplasm and facilitate further studies on KGM synthesis.

## Methods

### DNA isolation and sequencing

*Amorphophallus konjac* was obtained from the Daguan county (one of the main plantation areas of *A.konjac* in Yunnan), and cultivated in the glasshouse of Kunming University in Yunnan. Fresh leaves were collected from mature *A. konjac* plants and frozen in liquid nitrogen. Then genomic DNA was extracted from leaves using the cetyltrimethylammonium bromide (CTAB) method^41^. The integrity of the extracted DNA was checked by 0.75% agarose gel electrophoresis. The quantity and quality of the DNA were detected using a NanoDrop ND-2000 (NanoDrop products, Wilmington, DE, USA) and Qubit 2.0 Fluorometer (Invitrogen Ltd, Paisley, UK). Paired-end libraries with insert sizes of 325 bp, 434 bp, 529 bp, and 647 bp were constructed using NEBNext Ultra II DNA Library Prep Kit for Illumina (NEB, USA), and mate pair libraries with insert sizes of 3 kb, 7 kb, 12 kb, and 16 kb were constructed using Illumina Nextera Mate Pair Library Preparation Kit (Illumina, USA). All the constructed libraries were sequenced on a NovaSeq platform (Illumina, USA) using PE-150 module. In total, about 1119.58 Gb of data were generated on Illumina platforms.

All reads were preprocessed for quality control and filtered using the in-house Perl script. The raw data were filtered by removing reads with more than 5% N or more than 40 bp low-quality bases called below Q30. The redundant reads resulting in duplicate base calls were filtered; only one copy of any duplicated paired-end reads was retained. The yielded clean data were used for de novo assembly.

### Genome size estimation

Before genome assembly, we used Illumina short reads to estimate the genome size using a k-mer based method. An optimal k-mer value was obtained by Jellyfish^42^, and genome size was estimated using GenomeScope v2.0^43^ based on the 19-mer frequency distribution data. A 19-mer was the k-mer length recommended for use with the GenomeScope 2.0 program and was not adjusted because we had high coverage and a low error rate. The genome size was also estimated by flow cytometry using *Z*. *mays* as internal standard and propidium iodide as the stain.

### Assembly

The filtered reads were used to perform assembly with SOAPdenovo2^23^ developed by BGI. First, the contigs were constructed with *k*-mer = 47 using pair-end data, and the scaffolds were assembled with* k*-mer = 33 using both mate-pair and pair-end data. The final assembly was generated after gap filling with Gapcloser v1.12 in SOAPdenovo package^23^.

### Repeats annotation

First, the research team searched for tandem repeats across the genome using the program Tandem Repeat Finder (TRF)^44^. The transposable elements (TEs) in the genome were identified by a combination of homology-based and de novo approaches. For homolog-based prediction, known repeats were identified using RepeatMasker^45^ and RepeatProteinMask^45^ against Repbase16.10^46^. RepeatMasker was applied for DNA-level identification using a custom library. At the protein level, RepeatProteinMask was used to perform an RMBLAST search against the TE protein database. For de novo prediction, RepeatModeler (http://repeatmasker.org/) and LTR FINDER^47^ were used to identify de novo evolved repeats inferred from the assembled genome.

### Gene prediction and functional annotation

The research team employed EVidence Modeler (EVM)^48^ to consolidate RNA-seq, protein alignments with ab initio gene predictions and homologous method annotation into a final gene set. For transcriptome, reads were cleaned with Trimmomatic Version 0.32^49^. This step removed reads containing adapter, reads containing poly-N and low-quality reads from the raw data and yielded clean data for downstream analysis. Then, the reads were aligned to the genome with HISAT2 Version: 2.1.0^50^. Alignments were then assembled independently with StringTie Version: v1.3.3b^51^. Protein sequences of five plant species: *Arabidopsis thaliana*^52^, *Oryza sativa*^53^, *Zea mays*^54^, *Zostera marina*^21^ and *Spirodela polyrhiza*^20^ were used for the homology-based method. First, the tblastn was performed with e-value cutoff 1e-5, blast hits with low quality in the genome were discarded. Then predicted regions were extended by 2000 bp both upstream and downstream, and aligned against protein sequence using GeneWise^55^ to identify gene structure. The software AUGUSTUS^56^, GenScan^57^, GlimmerHMM^58^ and SNAP^59^ were used for ab initio gene prediction, AUGUSTUS and GenScan prediction used the gene model parameters trained on maize, but GlimmerHMM and SNAP prediction used gene model parameters trained on rice. All lines of evidence were then fed into EVM using intuitive weighting (RNAseq > cDNA/protein > ab initio gene predictions).

Gene functions were assigned according to the best match alignment using Blastp against Swiss-Prot, TrEMBL and KEGG databases. InterProScan functional analysis and Gene Ontology IDs were obtained using InterProScan^60^.

The GO enrichment was done with Ontologizer 2.0^61^ by using one-sided Fisher’s exact test, the Parent–Child-Union method, with a p-value cut-off of 0.05.

Genes related to cellulose synthase (*CesA*), cellulose synthase-like (*Csl*) were identified according to the InterProScan annotation, and the genes related to phenylpropanoid-lignin biosynthesis and starch and sucrose metabolism pathway were identified according to the KEGG annotation. Furthermore, the genes with alignment hits covering over 200 amino acids and at least 50% protein sequence identity were considered to be candidate genes.

### Non-coding gene annotation

Software tRNAscan-SE^62^ is specified for Eukaryotic tRNA and was deployed for tRNA annotation. The research team used homologous method to identify rRNA. The rRNA sequence data downloaded from Rfam database^63^ was used as a reference. INFERNAL^64^ was used to identify snRNA.

### Gene family cluster

To identify different sets of gene clusters, protein-coding genes sequences of *O. sativa*^53^, *Z. mays*^54^, *Z. marina*^21^and *S. polyrhiza*^20^ were used to locate gene clusters. After pairwise aligning using Blastp with an e-value cutoff of 1e-5 had been conducted, OrthoMCL package^65^ was performed to identify the gene family clusters using the Blastp output with default parameters, final paralogous and orthologous genes were defined using MCL software in OrthoMCL.

### Phylogenetic tree construction

Single-copy orthologous genes defined by OrthoMCL^65^ were formed, and then multiple single-copy genes were aligned using Muscle^66^ and the aligned sequences were extracted to feed to MrBayes (http://mrbayes.sourceforge.net) to infer the species phylogeny using a maximum likelihood (ML) approach under the best-fit model GTR + G from ModelFinder. *Z. mays* and *O. sativa* were used as outgroups. To estimate the divergence time of each species, the information about the already known divergence time data between these species from http://www.timetree.org/ were collected. The topology of the ML tree was fed to MCMCTREE in paml version 4.4^67^ for constructing a divergence time tree and calculate the divergence time. Based on the calculated phylogeny and the divergence time, CAFÉ (Computational Analysis of Gene Family Evolution, version 2.1)^68^, a tool based on the stochastic birth and death model for the statistical analysis of the evolution of gene family size, was applied to identify gene families that had undergone expansion and/or contraction.

### Detection of positively selected genes

To detect genes under positive selection, Blastn was performed to align the coding sequence (CDS) libraries of *Z. marina*^21^and *S. polyrhiza*^20^ against the *A. konjac* CDS library, respectively, in order to find the gene pairs with the best alignments. The resulting orthologous gene pairs were aligned again with the default parameters as a preparation for KaKs_Calculator 1.2^69^ which finally yielded a dataset of each gene pair’s Ka/Ks ratio, and the Ka/Ks ratio > 1 was defined as a positively selected gene (significance, P-value < 0.05).

### RNA-seq

Four tissues (namely tubers, fibres, stems and leaves) of *A. konjac* were harvested from the same 7-month-old plant, and three biological replicates for each tissue of living plants were collected. Total RNA was extracted from these tissues using the RNAprep pure plant kit (Tiangen). 3 μg of total RNA per sample were used as input material for the RNA sample preparation. Beads with oligo (dT) were used to isolate poly (A) mRNA from total RNA. RNA sequencing libraries were constructed from these mRNA using the TruSeq RNA Sample Preparation Kit (Illumina, San Diego, USA). Briefly, the Elution 2-Frag-Prime (94 °C for 8 min, 4 °C hold) was used to elute, fragment and prime the mRNA with Elute, Prime, Fragment Mix (Illumina). First strand cDNA synthesis was performed with First Strand Master Mix and SuperScript II mix (ratio: 1 μl SuperScript II/7 μl First Strand Master Mix) (Invitrogen). The second strand was synthesized with Second Strand Master Mix (Illumina) and Ampure XP beads (Illumina) were used to separate the double-stranded (ds) cDNA from the 2nd strand reaction mix. After end repair and the addition of a 3’-dA overhang, the cDNA was ligated to Illumina PE adapter oligo mix (Illumina), and size-selected for 350 ± 20 bp fragments by gel purification. After 15 cycles of PCR amplification, the 350 bp paired-end libraries were sequenced using the paired-end sequencing module (150 bp at each end) of the Illumina HiSeq 4000 platform.

The corresponding trimmed clean reads were aligned to the related reference genome employing TopHat2^24^ software with default settings. Calculation of gene expression level was conducted using Cufflinks v2.2.1^25^. Fragments per kilobase of exon per million fragments mapped (FPKM) were used to normalize RNA-seq fragment counts and estimate the relative abundance of each gene. The DEGs were decided based on a P-value < 0.05 and at least a twofold change between the two FPKMs.

### Ethical approval

We confirm that all the experimental research and field studies on plants (either cultivated or wild), including the collection of plant material, complied with relevant institutional, national, and international guidelines and legislation. The tuber of *A. konjac* was collected from Daguan county, and was cultured in the green house. All the material is owned by the authors and/or no permissions are required.

### Supplementary Information

Supplementary Tables.

Supplementary Figures.

## Data Availability

Accession numbers: The genome sequence of *A. konjac* has been deposited in DDBJ/EMBL/GenBank nucleotide core database under accession code SUB7124908 (https://www.ncbi.nlm.nih.gov/sra/PRJNA608095). The sequencing reads of Illumina sequencing libraries have been deposited under NCBI Sequence Read Archive with Project ID PRJNA608095. The Project ID of all the RNA-seq data is SRP251185.
