# Supplementary material for: Characteristics of Amorphophallus konjac as indicated by its genome
Source: Sci Rep. 2023 Dec 19;13:22684. doi: 10.1038/s41598-023-49963-9 (PMC10730839; doi:10.1038/s41598-023-49963-9)
Supplement: Supplementary file 1 — Supplementary Tables. [file 41598_2023_49963_MOESM1_ESM.docx]

Supplementary Tables

**Table S1.** Statistics of Illumina HiSeq raw data.

| Lib-ID | Read Length(bp) | Insert Size(bp) | Raw Data (Gb) | Clean Data (Gb) | Q20(%) | GC Content (%) |
| --- | --- | --- | --- | --- | --- | --- |
| DMY1 | 150 | 325 | 110.52 | 106.79 | 96.43 | 49.05 |
| DMY2 | 150 | 434 | 127.16 | 123.84 | 96.03 | 49.13 |
| DMY3 | 150 | 529 | 119.21 | 116.88 | 95.03 | 48.39 |
| DMY4 | 150 | 647 | 118.22 | 115.01 | 95.14 | 47.89 |
| MMY1 | 150 | 3000 | 136.02 | 133.79 | 94.61 | 46.80 |
| MMY2 | 150 | 7000 | 226.93 | 222.21 | 94.23 | 46.38 |
| MMY3 | 150 | 12000 | 131.52 | 130.48 | 95.14 | 45.58 |
| MMY4 | 150 | 16000 | 172.02 | 170.56 | 94.88 | 44.95 |
| Total | - | - | 1,141.61 | 1,119.58 | - | - |

**Table S2.** Statistics for the assembled genome using SOAPdenovo2.

|  | Contig (k=47) | | Scaffold (k=33) | |
| --- | --- | --- | --- | --- |
|  | Size (bp) | Number | Size (bp) | Number |
| N90 | 110 | 12,500,616 | 144 | 3,495,698 |
| N80 | 123 | 9,927,526 | 472 | 1,447,682 |
| N70 | 139 | 7,638,334 | 1,020 | 796,207 |
| N60 | 159 | 5,619,036 | 1,796 | 453,608 |
| N50 | 189 | 3,886,294 | 3,212 | 260,787 |
| Longest | 18827 | | 85,347 | |
| Total Size | 2,992,250,072 | | 4,584,988,971 | |
| Total Number | 15,362,644 | | 7,423,768 | |
| Total Number (>=2kb) | 44,100 | | 4,09,330 | |
| Total Number (>=5kb) | 4,004 | | 175,774 | |

**Table S3.** Statistics of the completeness of assembled genome based on 248 CEGs.

|  | Prots | %Completeness | Total | Average | %Ortho |
| --- | --- | --- | --- | --- | --- |
| Partial | 100 | 40.32 | 199 | 1.99 | 51.00 |
| Group 1 | 19 | 28.79 | 38 | 2 | 57.89 |
| Group 2 | 19 | 33.93 | 29 | 1.53 | 31.58 |
| Group 3 | 22 | 36.07 | 48 | 2.18 | 59.09 |
| Group 4 | 40 | 61.54 | 84 | 2.1 | 52.5 |
| Complete | 188 | 75.81 | 509 | 2.71 | 71.81 |
| Group 1 | 38 | 57.58 | 91 | 2.39 | 63.16 |
| Group 2 | 42 | 75 | 104 | 2.48 | 66.67 |
| Group 3 | 51 | 83.61 | 126 | 2.47 | 70.59 |
| Group 4 | 57 | 87.69 | 188 | 3.3 | 82.46 |

Prots = number of 248 ultra-conserved CEGs present in genome;

%Completeness = percentage of 248 ultra-conserved CEGs present;

Total = total number of CEGs present including putative orthologs;

Average = average number of orthologs per CEG;

%Ortho = percentage of detected CEGs that have more than 1 ortholog

**Table S4.** Statistics of BUSCOs.

|  | Gene numbers | Percentage |
| --- | --- | --- |
| Complete BUSCOs | 624 | 43.4% |
| Complete and single-copy BUSCOs | 558 | 38.8% |
| Complete and duplicated BUSCOs | 66 | 4.6% |
| Fragmented BUSCOs | 212 | 14.7% |
| Missing BUSCOs | 604 | 41.9% |
| Total BUSCO groups searched | 1440 | - |

BUSCO lineage data: embryophyta_odb9

**Table S5.** Statistics of repeats in *A. konjac* genome.

| Type | Repeat Size (bp) | Percent of genome (%) |
| --- | --- | --- |
| Trf | 278,335,607 | 6.07 |
| Repeatmasker | 264,872,553 | 5.78 |
| Proteinmask | 503,822,143 | 10.99 |
| *De novo* | 3,254,732,320 | 70.98 |
| Total | 3,289,511,160 | 71.75 |

**Table S6**. TEs content in the assembled *A. konjac* genome

| **Type** | **Length (bp)** | **% in genome** |
| --- | --- | --- |
| DNA | 591,083,695 | 12.89 |
| LINE | 265,731,983 | 5.8 |
| SINE | 10,222,276 | 0.22 |
| Total LTR | 2,386,877,537 | 52.06 |
| Ty3-gypsy | 1,440,562,663 | 31.42 |
| Ty1-copia | 511,670,252 | 11.16 |
| other LTR | 434,644,622 | 9.48 |
| Other | 17,814 | 0 |
| Unknown | 298,267,384 | 6.51 |
| Total | 3,170,823,759 | 69.16 |

**Table S7.** Overview of *A. konjac* gene annotation.

| Method | Type | No. of genes | average gene length | No. of exon | average exon length | average cds length | average exon number | total intron length |
| --- | --- | --- | --- | --- | --- | --- | --- | --- |
| Denovo | augustus | 19,269 | 1,257.53 | 41,163 | 276.91 | 591.54 | 2.14 | 12,832,884 |
|  | glimmerHMM | 137,953 | 1,569.29 | 308,729 | 160.03 | 358.13 | 2.24 | 167,082,495 |
|  | genscan | 14,782 | 2,155.04 | 36,418 | 182.73 | 450.19 | 2.46 | 25,200,990 |
|  | snap | 75,640 | 1,012.25 | 141,246 | 211.19 | 394.37 | 1.87 | 46,736,220 |
| Homolog | *A. thaliana* | 31,710 | 1,380.84 | 78,461 | 236.67 | 585.61 | 2.47 | 25,216,757 |
|  | *O. sativa* | 40,457 | 1,140.58 | 88,898 | 234.70 | 515.73 | 2.20 | 25,279,645 |
|  | *Z. mays* | 44,585 | 1,142.56 | 100,218 | 222.36 | 499.82 | 2.25 | 28,656,603 |
|  | *S. polyrhiza* | 36,598 | 1,372.51 | 89,685 | 238.54 | 584.56 | 2.45 | 28,837,435 |
|  | *Z. marina* | 30,885 | 1,415.72 | 77,323 | 234.67 | 587.50 | 2.50 | 25,579,307 |
| Transcriptome | transcript | 97,311 | 1,721.80 | 216,212 | 327.62 | 727.93 | 2.22 | 96,713,980 |
| Merge | EVM | 39,241 | 1,372.75 | 90,416 | 257.08 | 589.65 | 2.29 | 30,870,726 |

**Supplementary Table S8.** Statistics of gene functional annotation.

|  | Number | Percent (%) |
| --- | --- | --- |
| Total | 39,421 | 100.00 |
| InterProScan | 25,797 | 65.44 |
| KEGG | 26,512 | 67.25 |
| Swiss-Prot | 26,456 | 67.11 |
| Trembl | 33,715 | 85.53 |
| Annotated | 34,126 | 86.57 |
| Unannotated | 5,295 | 13.43 |

**Table S9.** Statistics of predicted non-coding RNA.

| Type |  | Copy(w) | | Average length(bp) | Total length (bp) | % of genome |
| --- | --- | --- | --- | --- | --- | --- |
| miRNA |  | | 1,078 | 99.21 | 106,947 | 0.002333 |
| tRNA |  | | 761 | 74.97 | 57,053 | 0.001244 |
| rRNA | rRNA | | 2,894 | 65.99 | 190,977 | 0.004165 |
|  | 18S | | 296 | 101.97 | 30,184 | 0.000658 |
|  | 28S | | 112 | 80.71 | 9,039 | 0.000197 |
|  | 5.8S | | 27 | 88.44 | 2,388 | 0.000052 |
|  | 5S | | 2,459 | 60.74 | 149,366 | 0.003258 |
| snRNA | snRNA | | 1,553 | 109.23 | 169,629 | 0.0037 |
|  | CD-box | | 1,313 | 106.06 | 139,252 | 0.003037 |
|  | HACA-box | | 47 | 134.81 | 6,336 | 0.000138 |
|  | splicing | | 192 | 124.51 | 23,905 | 0.000521 |

**Table S10**. Statistics of gene family cluster.

| Species | Genes number | Genes in families | Unclustered genes | Family number | Unique families | Average genes per family |
| --- | --- | --- | --- | --- | --- | --- |
| *Amorphophallus konjac* | 39,421 | 22,730 | 16,691 | 13,190 | 3,001 | 2 |
| *Zosma marina* | 25,000 | 18,782 | 6,218 | 11,474 | 1,075 | 2 |
| *Oryza sativa* | 23,639 | 17,492 | 6,147 | 12,719 | 432 | 1 |
| *Spirodela polyrhiza* | 19,620 | 16,138 | 3,482 | 11,580 | 364 | 1 |
| *Zea mays* | 39,254 | 31,060 | 8,194 | 15,436 | 2,018 | 2 |

**Table S11.** The positively selected genes of *A. konjac* against *S. polyrhiza*.

| Sequence | Ka | Ks | Ka/Ks | P-Value(Fisher) | Length | S-Sites | N-Sites |
| --- | --- | --- | --- | --- | --- | --- | --- |
| C423421426.1_Spipo13G0014100 | 0.36 | 0.08 | 4.32 | 7.88E-05 | 201 | 73.48 | 127.52 |
| C423876172.1_Spipo24G0016500 | 0.41 | 0.11 | 3.61 | 0.0002374 | 183 | 70.63 | 112.37 |
| C424806696.1_Spipo20G0037100 | 0.36 | 0.08 | 4.72 | 0.0001769 | 186 | 62.27 | 123.73 |
| C425334002.1_Spipo6G0075100 | 0.61 | 0.26 | 2.35 | 1.39E-05 | 288 | 124.58 | 163.42 |
| C425485632.1_Spipo24G0002900 | 0.20 | 0.04 | 4.41 | 0.0033959 | 213 | 87.72 | 125.28 |
| C425658710.1_Spipo2G0103600 | 0.23 | 0.01 | 20.51 | 3.26E-05 | 183 | 76.68 | 106.32 |
| C425669356.1_Spipo12G0033500 | 0.20 | 0.03 | 6.64 | 0.0001692 | 225 | 104.92 | 120.08 |
| C425826784.1_Spipo0G0052100 | 0.47 | 0.21 | 2.23 | 0.0016787 | 198 | 91.44 | 106.56 |
| C425859202.1_Spipo22G0026300 | 0.24 | 0.06 | 3.75 | 0.0050831 | 171 | 54.66 | 116.34 |
| C425891523.1_Spipo0G0005700 | 0.24 | 0.05 | 4.66 | 1.32E-05 | 369 | 132.77 | 236.23 |
| C425901541.1_Spipo0G0076400 | 0.26 | 0.13 | 2.00 | 0.0066892 | 414 | 163.60 | 250.40 |
| C425985195.1_Spipo3G0053800 | 0.29 | 0.06 | 4.89 | 1.78E-06 | 321 | 118.19 | 202.81 |
| C426089147.1_Spipo23G0029800 | 0.54 | 0.05 | 11.64 | 2.15E-09 | 171 | 81.70 | 89.30 |
| C426146759.1_Spipo2G0039900 | 0.44 | 0.05 | 8.40 | 4.76E-15 | 438 | 213.64 | 224.36 |
| C426233109.1_Spipo0G0035800 | 0.35 | 0.14 | 2.50 | 1.70E-05 | 456 | 166.80 | 289.21 |
| C426308339.1_Spipo0G0035000 | 0.24 | 0.05 | 4.74 | 6.45E-06 | 357 | 131.90 | 225.10 |
| C426316759.1_Spipo9G0025200 | 0.45 | 0.14 | 3.35 | 7.30E-05 | 249 | 78.62 | 170.39 |
| C426318703.1_Spipo17G0019500 | 0.22 | 0.02 | 14.37 | 4.86E-09 | 483 | 165.88 | 317.12 |
| C426401145.1_Spipo1G0018200 | 0.44 | 0.15 | 2.91 | 3.51E-07 | 456 | 155.83 | 300.17 |
| C426445755.1_Spipo23G0020600 | 0.59 | 0.04 | 15.30 | 1.19E-23 | 438 | 220.95 | 217.05 |
| C426467787.1_Spipo8G0019600 | 0.47 | 0.28 | 1.71 | 0.0013231 | 474 | 165.35 | 308.65 |
| C426564017.1_Spipo0G0052100 | 0.41 | 0.17 | 2.38 | 0.0026942 | 186 | 87.08 | 98.92 |
| C426564089.1_Spipo3G0030400 | 0.55 | 0.32 | 1.71 | 0.0015656 | 426 | 127.71 | 298.29 |
| C426581535.1_Spipo9G0044200 | 0.40 | 0.08 | 5.32 | 3.56E-15 | 567 | 238.84 | 328.16 |
| C426585465.1_Spipo3G0047300 | 0.29 | 0.12 | 2.43 | 0.0001569 | 465 | 161.94 | 303.06 |
| C426633457.1_Spipo2G0032500 | 0.56 | 0.37 | 1.52 | 0.0031249 | 513 | 180.21 | 332.79 |
| C426656669.1_Spipo9G0009800 | 0.58 | 0.37 | 1.57 | 0.0012211 | 513 | 194.41 | 318.59 |
| C426674371.1_Spipo15G0005100 | 0.22 | 0.02 | 10.41 | 1.49E-08 | 429 | 160.60 | 268.40 |
| C426674371.1_Spipo20G0033000 | 0.21 | 0.01 | 18.99 | 2.39E-10 | 429 | 183.12 | 245.88 |
| C426721707.1_Spipo1G0101800 | 0.23 | 0.04 | 5.18 | 8.97E-08 | 570 | 204.71 | 365.29 |
| C426734419.1_Spipo0G0078000 | 0.38 | 0.06 | 6.36 | 1.60E-15 | 627 | 252.71 | 374.29 |
| C426848137.1_Spipo3G0061200 | 0.24 | 0.04 | 5.52 | 4.46E-09 | 540 | 224.76 | 315.24 |
| C426848137.1_Spipo8G0015300 | 0.45 | 0.10 | 4.49 | 0.0001008 | 150 | 70.22 | 79.78 |
| C426848637.1_Spipo0G0182400 | 0.28 | 0.06 | 4.62 | 0.0001588 | 255 | 82.78 | 172.22 |
| C426848637.1_Spipo22G0034400 | 0.97 | 0.31 | 3.11 | 0.0001417 | 165 | 63.30 | 101.70 |
| C426869763.1_Spipo1G0036600 | 0.43 | 0.14 | 3.03 | 7.44E-05 | 267 | 101.16 | 165.84 |
| C426873349.1_Spipo2G0116400 | 0.25 | 0.08 | 3.04 | 7.19E-06 | 516 | 209.83 | 306.17 |
| C426892993.1_Spipo3G0034400 | 0.52 | 0.29 | 1.78 | 7.72E-06 | 684 | 253.38 | 430.62 |
| C426894041.1_Spipo1G0080900 | 0.36 | 0.08 | 4.63 | 3.62E-11 | 585 | 197.05 | 387.95 |
| C426905909.1_Spipo0G0068300 | 0.74 | 0.37 | 2.02 | 0.0009832 | 204 | 83.71 | 120.29 |
| C426906755.1_Spipo16G0001700 | 0.43 | 0.27 | 1.55 | 0.0068462 | 534 | 174.54 | 359.46 |
| C426924557.1_Spipo4G0001000 | 0.36 | 0.15 | 2.32 | 1.09E-06 | 678 | 247.41 | 430.59 |
| C426933497.1_Spipo0G0056100 | 0.45 | 0.20 | 2.23 | 3.83E-05 | 456 | 145.68 | 310.32 |
| C426951457.1_Spipo0G0090200 | 0.30 | 0.12 | 2.47 | 7.26E-06 | 594 | 226.53 | 367.47 |
| C426955743.1_Spipo0G0126300 | 0.76 | 0.10 | 7.37 | 9.15E-14 | 270 | 105.97 | 164.03 |
| C426958767.1_Spipo2G0119700 | 0.44 | 0.28 | 1.54 | 0.0016194 | 687 | 233.83 | 453.17 |
| C426979597.1_Spipo23G0043000 | 0.31 | 0.06 | 5.15 | 1.87E-07 | 357 | 147.01 | 209.99 |
| C427010647.1_Spipo22G0034400 | 0.65 | 0.28 | 2.34 | 0.0015609 | 195 | 62.15 | 132.85 |
| C427021347.1_Spipo5G0052300 | 0.54 | 0.23 | 2.30 | 1.72E-08 | 627 | 223.28 | 403.72 |
| C427026343.1_Spipo13G0030500 | 0.38 | 0.23 | 1.68 | 0.0002222 | 765 | 271.95 | 493.05 |
| C427044041.1_Spipo0G0080900 | 0.39 | 0.18 | 2.17 | 1.52E-07 | 786 | 298.90 | 487.10 |
| C427060067.1_Spipo13G0015600 | 0.55 | 0.29 | 1.92 | 3.29E-06 | 627 | 219.72 | 407.28 |
| C427064219.1_Spipo29G0025700 | 0.43 | 0.18 | 2.38 | 1.26E-10 | 861 | 373.46 | 487.54 |
| C427065493.1_Spipo2G0044900 | 0.78 | 0.20 | 3.97 | 1.19E-07 | 201 | 66.64 | 134.36 |
| C427070827.1_Spipo16G0001500 | 0.43 | 0.20 | 2.15 | 5.40E-05 | 429 | 175.71 | 253.29 |
| C427076827.1_Spipo17G0047000 | 0.37 | 0.10 | 3.83 | 3.83E-09 | 570 | 193.08 | 376.92 |
| C427082025.1_Spipo28G0017900 | 0.33 | 0.14 | 2.31 | 1.13E-06 | 705 | 261.72 | 443.28 |
| C427084997.1_Spipo13G0029800 | 0.50 | 0.29 | 1.76 | 0.0007793 | 444 | 158.94 | 285.06 |
| C427087091.1_Spipo17G0016600 | 0.27 | 0.01 | 50.00 | 1.61E-05 | 165 | 55.17 | 109.83 |
| C427088147.1_Spipo12G0063100 | 0.58 | 0.13 | 4.62 | 3.32E-14 | 516 | 181.44 | 334.56 |
| C427098455.1_Spipo13G0048900 | 0.17 | 0.02 | 7.56 | 0.0006224 | 297 | 84.82 | 212.18 |
| C427109009.1_Spipo17G0045500 | 0.21 | 0.03 | 7.50 | 7.87E-05 | 309 | 106.21 | 202.79 |
| C427114817.1_Spipo3G0030400 | 0.51 | 0.31 | 1.65 | 0.0002629 | 627 | 228.73 | 398.27 |
| C427152375.1_Spipo11G0044100 | 0.24 | 0.06 | 4.12 | 8.12E-13 | 1017 | 390.79 | 626.21 |
| C427152375.1_Spipo16G0022000 | 0.24 | 0.07 | 3.58 | 1.21E-11 | 1020 | 409.45 | 610.55 |
| C427174201.1_Spipo23G0002400 | 0.25 | 0.09 | 2.68 | 2.37E-08 | 960 | 388.62 | 571.38 |
| C427194585.1_Spipo15G0000400 | 0.47 | 0.15 | 3.19 | 1.00E-06 | 381 | 133.44 | 247.56 |
| C427203430.1_Spipo3G0061600 | 0.28 | 0.09 | 3.32 | 3.09E-12 | 1050 | 378.95 | 671.05 |
| C427209536.1_Spipo4G0108800 | 0.48 | 0.17 | 2.82 | 2.55E-11 | 747 | 258.12 | 488.88 |
| C427219334.1_Spipo17G0011900 | 0.47 | 0.04 | 13.45 | 1.49E-31 | 750 | 299.99 | 450.01 |
| C427222954.1_Spipo18G0029400 | 0.36 | 0.11 | 3.14 | 4.36E-13 | 1158 | 336.91 | 821.09 |
| C427242632.1_Spipo2G0105300 | 0.35 | 0.12 | 2.86 | 7.13E-10 | 753 | 299.17 | 453.83 |
| C427250466.1_Spipo5G0018400 | 0.23 | 0.09 | 2.71 | 1.83E-07 | 1296 | 300.58 | 995.42 |
| C427264124.1_Spipo22G0003900 | 0.26 | 0.06 | 4.09 | 3.59E-07 | 522 | 185.94 | 336.06 |
| C427264226.1_Spipo1G0089500 | 0.30 | 0.17 | 1.71 | 0.0002665 | 957 | 324.40 | 632.60 |
| C427270956.1_Spipo1G0039600 | 0.23 | 0.09 | 2.64 | 7.10E-08 | 996 | 369.63 | 626.37 |
| C427280928.1_Spipo19G0018700 | 0.69 | 0.48 | 1.43 | 5.80E-05 | 1086 | 322.31 | 763.69 |
| C427284196.1_Spipo2G0044300 | 0.36 | 0.10 | 3.46 | 2.66E-13 | 831 | 316.87 | 514.13 |
| C427287220.1_Spipo0G0033500 | 0.43 | 0.22 | 1.97 | 4.73E-07 | 909 | 281.09 | 627.92 |
| C427288350.1_Spipo9G0048600 | 0.56 | 0.22 | 2.54 | 6.58E-13 | 864 | 268.92 | 595.09 |
| C427298574.1_Spipo0G0099500 | 0.36 | 0.07 | 5.13 | 1.80E-15 | 717 | 274.64 | 442.36 |
| C427306278.1_Spipo5G0068600 | 0.24 | 0.06 | 3.81 | 2.77E-10 | 828 | 323.69 | 504.31 |
| C427312310.1_Spipo16G0042600 | 0.30 | 0.06 | 5.29 | 4.52E-17 | 954 | 340.80 | 613.21 |
| C427314142.1_Spipo12G0018900 | 0.65 | 0.38 | 1.71 | 1.39E-07 | 897 | 321.82 | 575.18 |
| C427314142.1_Spipo16G0045600 | 0.73 | 0.54 | 1.34 | 0.0071018 | 738 | 234.95 | 503.06 |
| C427332016.1_Spipo4G0091700 | 0.48 | 0.06 | 7.69 | 7.62E-23 | 825 | 257.47 | 567.53 |
| C427332016.1_Spipo4G0091800 | 0.49 | 0.06 | 7.55 | 2.22E-22 | 822 | 251.56 | 570.44 |
| C427332016.1_Spipo4G0092200 | 0.43 | 0.06 | 6.69 | 3.87E-22 | 831 | 283.87 | 547.13 |
| C427332016.1_Spipo4G0092400 | 0.42 | 0.08 | 5.45 | 9.59E-18 | 822 | 251.14 | 570.86 |
| C427332016.1_Spipo4G0092500 | 0.42 | 0.07 | 6.40 | 1.12E-20 | 828 | 272.37 | 555.63 |
| C427341948.1_Spipo10G0022300 | 0.64 | 0.25 | 2.54 | 1.74E-14 | 855 | 297.49 | 557.51 |
| C427346454.1_Spipo1G0063600 | 0.31 | 0.14 | 2.19 | 2.11E-05 | 594 | 244.93 | 349.07 |
| C427354852.1_Spipo13G0039000 | 0.32 | 0.09 | 3.56 | 3.12E-13 | 960 | 339.05 | 620.95 |
| C427357010.1_Spipo3G0089500 | 0.35 | 0.03 | 12.09 | 4.77E-30 | 1074 | 393.92 | 680.08 |
| C427357010.1_Spipo3G0089600 | 0.36 | 0.03 | 13.86 | 1.28E-31 | 1083 | 396.69 | 686.32 |
| C427358838.1_Spipo6G0068900 | 0.29 | 0.13 | 2.17 | 0.0001312 | 642 | 188.78 | 453.22 |
| C427360570.1_Spipo19G0014800 | 0.23 | 0.08 | 2.95 | 1.67E-05 | 531 | 207.17 | 323.83 |
| C427366420.1_Spipo3G0035900 | 0.39 | 0.13 | 3.02 | 7.03E-26 | 1995 | 679.76 | 1315.25 |
| C427374694.1_Spipo0G0080900 | 0.37 | 0.20 | 1.85 | 2.54E-08 | 1395 | 484.71 | 910.29 |
| C427376218.1_Spipo1G0003400 | 0.48 | 0.26 | 1.89 | 0.0082632 | 330 | 93.16 | 236.84 |
| C427395144.1_Spipo14G0046300 | 0.45 | 0.29 | 1.56 | 9.70E-09 | 2145 | 720.41 | 1424.59 |
| C427395144.1_Spipo14G0046300 | 1.06 | 0.41 | 2.57 | 4.75E-14 | 531 | 202.82 | 328.18 |
| C427395144.1_Spipo14G0046300 | 0.76 | 0.56 | 1.37 | 0.006439 | 507 | 183.51 | 323.50 |
| C427397392.1_Spipo17G0013300 | 0.20 | 0.03 | 6.40 | 0.0010048 | 204 | 71.23 | 132.78 |
| C427401444.1_Spipo0G0105200 | 0.41 | 0.28 | 1.48 | 5.12E-07 | 2298 | 690.78 | 1607.22 |
| C427401444.1_Spipo24G0014100 | 0.50 | 0.18 | 2.78 | 1.12E-31 | 2235 | 717.90 | 1517.10 |
| C427401444.1_Spipo27G0020300 | 0.41 | 0.20 | 2.05 | 5.69E-17 | 2340 | 722.89 | 1617.11 |
| C427403522.1_Spipo4G0087000 | 0.38 | 0.15 | 2.59 | 1.78E-13 | 1425 | 428.45 | 996.55 |
| scaffold1000061.1_Spipo13G0031500 | 0.24 | 0.00 | 50.00 | 0.0003009 | 195 | 44.54 | 150.46 |
| scaffold1006481.1_Spipo1G0068000 | 0.38 | 0.13 | 2.91 | 2.31E-10 | 870 | 271.90 | 598.10 |
| scaffold1006566.1_Spipo0G0181800 | 0.30 | 0.06 | 4.91 | 8.58E-07 | 390 | 125.64 | 264.36 |
| scaffold1006566.1_Spipo1G0051000 | 0.37 | 0.09 | 3.92 | 6.75E-09 | 504 | 165.58 | 338.42 |
| scaffold1006566.1_Spipo23G0035700 | 0.24 | 0.10 | 2.40 | 0.0013172 | 447 | 138.86 | 308.14 |
| scaffold1009646.1_Spipo0G0004300 | 0.35 | 0.11 | 3.34 | 5.46E-20 | 1626 | 535.67 | 1090.33 |
| scaffold1012606.1_Spipo32G0009100 | 0.44 | 0.30 | 1.49 | 0.0009609 | 879 | 332.85 | 546.15 |
| scaffold1017408.1_Spipo19G0009400 | 0.31 | 0.11 | 2.80 | 4.89E-05 | 444 | 132.90 | 311.10 |
| scaffold1021781.1_Spipo5G0072400 | 0.24 | 0.08 | 2.84 | 1.58E-06 | 906 | 244.04 | 661.96 |
| scaffold1029861.1_Spipo11G0056700 | 0.34 | 0.11 | 3.24 | 2.32E-20 | 1545 | 572.12 | 972.88 |
| scaffold1032172.1_Spipo4G0012200 | 0.24 | 0.10 | 2.45 | 0.0002725 | 597 | 162.10 | 434.90 |
| scaffold1033275.1_Spipo6G0038200 | 0.34 | 0.16 | 2.16 | 5.37E-07 | 1104 | 265.88 | 838.12 |
| scaffold1034639.1_Spipo3G0047700 | 0.26 | 0.04 | 7.25 | 1.43E-11 | 828 | 226.17 | 601.83 |
| scaffold1038368.1_Spipo23G0010300 | 0.22 | 0.09 | 2.37 | 0.0002111 | 678 | 194.43 | 483.57 |
| scaffold1043483.1_Spipo0G0125200 | 0.37 | 0.05 | 7.09 | 6.13E-08 | 294 | 107.03 | 186.97 |
| scaffold1043483.1_Spipo0G0180300 | 0.37 | 0.23 | 1.63 | 0.0015312 | 708 | 232.16 | 475.85 |
| scaffold1053117.1_Spipo7G0018400 | 0.27 | 0.08 | 3.26 | 3.48E-13 | 1293 | 408.22 | 884.78 |
| scaffold1060700.1_Spipo12G0020300 | 1.45 | 0.11 | 13.56 | 3.75E-14 | 276 | 96.89 | 179.11 |
| scaffold1064522.1_Spipo0G0126700 | 0.37 | 0.15 | 2.47 | 6.32E-07 | 633 | 215.46 | 417.55 |
| scaffold1070225.1_Spipo4G0013600 | 0.36 | 0.12 | 2.91 | 1.33E-06 | 678 | 166.21 | 511.79 |
| scaffold1070319.1_Spipo15G0026600 | 1.21 | 0.36 | 3.39 | 0.0099087 | 117 | 29.00 | 88.00 |
| scaffold1073187.2_Spipo0G0053900 | 0.40 | 0.15 | 2.61 | 1.08E-20 | 1875 | 648.79 | 1226.21 |
| scaffold107371.1_Spipo25G0006100 | 0.38 | 0.15 | 2.48 | 1.03E-14 | 1476 | 545.12 | 930.88 |
| scaffold107536.1_Spipo23G0012800 | 0.46 | 0.13 | 3.44 | 1.12E-18 | 912 | 397.52 | 514.48 |
| scaffold1075823.1_Spipo10G0042300 | 0.50 | 0.17 | 3.04 | 1.08E-14 | 867 | 295.26 | 571.74 |
| scaffold1081586.1_Spipo1G0038400 | 0.21 | 0.06 | 3.40 | 5.20E-05 | 564 | 150.85 | 413.15 |
| scaffold1082039.1_Spipo1G0098400 | 0.31 | 0.16 | 1.93 | 0.0001544 | 711 | 237.16 | 473.84 |
| scaffold1083932.1_Spipo13G0029800 | 0.75 | 0.19 | 3.93 | 7.47E-08 | 282 | 96.77 | 185.23 |
| scaffold1087725.1_Spipo17G0043000 | 0.48 | 0.11 | 4.40 | 1.02E-40 | 1770 | 683.02 | 1086.98 |
| scaffold1087725.1_Spipo22G0037100 | 0.48 | 0.26 | 1.83 | 1.81E-11 | 1659 | 576.74 | 1082.26 |
| scaffold1094790.1_Spipo0G0033500 | 0.45 | 0.30 | 1.47 | 4.29E-07 | 2148 | 682.37 | 1465.63 |
| scaffold1097254.1_Spipo2G0019200 | 0.42 | 0.16 | 2.62 | 1.76E-06 | 471 | 174.08 | 296.92 |
| scaffold1097724.1_Spipo32G0010300 | 0.28 | 0.08 | 3.34 | 7.10E-08 | 801 | 222.12 | 578.89 |
| scaffold1098471.1_Spipo1G0002200 | 0.18 | 0.07 | 2.50 | 0.0068932 | 459 | 114.68 | 344.32 |
| scaffold1100306.1_Spipo9G0066900 | 0.30 | 0.13 | 2.27 | 0.0029346 | 381 | 118.85 | 262.15 |
| scaffold1107565.1_Spipo26G0018200 | 0.26 | 0.08 | 3.16 | 9.46E-06 | 519 | 158.82 | 360.18 |
| scaffold1108740.1_Spipo1G0003500 | 0.18 | 0.06 | 3.07 | 0.0004302 | 444 | 162.49 | 281.51 |
| scaffold1110248.1_Spipo11G0050100 | 0.18 | 0.00 | 50.00 | 0.0018602 | 192 | 45.17 | 146.83 |
| scaffold1116779.1_Spipo4G0059200 | 0.15 | 0.00 | 50.00 | 0.0022179 | 174 | 60.73 | 113.27 |
| scaffold1120399.1_Spipo24G0006300 | 0.44 | 0.27 | 1.63 | 0.0006624 | 648 | 230.16 | 417.84 |
| scaffold1121273.1_Spipo6G0033600 | 0.24 | 0.10 | 2.50 | 0.0010266 | 411 | 153.57 | 257.43 |
| scaffold1122392.1_Spipo18G0036900 | 0.35 | 0.14 | 2.55 | 2.05E-06 | 597 | 246.77 | 350.23 |
| scaffold1123077.2_Spipo23G0006700 | 0.26 | 0.08 | 3.11 | 1.40E-05 | 543 | 165.03 | 377.97 |
| scaffold1126692.1_Spipo22G0014900 | 0.47 | 0.10 | 4.56 | 6.85E-27 | 1296 | 434.10 | 861.91 |
| scaffold1127208.1_Spipo3G0023400 | 0.30 | 0.11 | 2.64 | 6.64E-06 | 582 | 197.71 | 384.29 |
| scaffold1132681.1_Spipo3G0089300 | 0.46 | 0.27 | 1.68 | 0.0001406 | 825 | 236.89 | 588.12 |
| scaffold1135681.1_Spipo1G0111200 | 0.47 | 0.12 | 3.80 | 0.0001261 | 219 | 76.97 | 142.03 |
| scaffold1136263.1_Spipo0G0052100 | 0.53 | 0.22 | 2.38 | 0.0009728 | 237 | 73.98 | 163.02 |
| scaffold1136263.1_Spipo0G0121500 | 0.41 | 0.19 | 2.18 | 0.0042873 | 279 | 88.10 | 190.90 |
| scaffold1136263.1_Spipo0G0126300 | 0.42 | 0.21 | 2.01 | 0.0018638 | 315 | 116.06 | 198.94 |
| scaffold1136263.1_Spipo14G0034500 | 0.50 | 0.07 | 7.43 | 5.65E-07 | 207 | 71.56 | 135.44 |
| scaffold1140537.2_Spipo7G0024400 | 0.59 | 0.22 | 2.72 | 1.61E-05 | 249 | 91.75 | 157.25 |
| scaffold1149930.1_Spipo29G0020500 | 0.73 | 0.39 | 1.87 | 0.0030977 | 189 | 77.66 | 111.34 |
| scaffold1152633.1_Spipo0G0133200 | 0.36 | 0.10 | 3.46 | 0.0032475 | 249 | 53.38 | 195.62 |
| scaffold116079.1_Spipo16G0045900 | 0.46 | 0.13 | 3.46 | 3.94E-22 | 1140 | 441.39 | 698.61 |
| scaffold1169180.1_Spipo24G0011300 | 0.24 | 0.07 | 3.57 | 1.10E-10 | 1032 | 343.21 | 688.79 |
| scaffold1169782.1_Spipo6G0053700 | 0.28 | 0.18 | 1.59 | 0.0082693 | 927 | 214.17 | 712.83 |
| scaffold1177285.1_Spipo17G0029800 | 0.42 | 0.08 | 5.35 | 1.25E-11 | 441 | 189.00 | 252.00 |
| scaffold1177385.1_Spipo16G0024400 | 0.29 | 0.15 | 1.90 | 0.0043863 | 495 | 134.77 | 360.23 |
| scaffold1177639.1_Spipo3G0101200 | 0.38 | 0.11 | 3.37 | 3.58E-10 | 801 | 225.64 | 575.36 |
| scaffold1178662.1_Spipo14G0034500 | 0.49 | 0.20 | 2.47 | 0.0005085 | 246 | 89.04 | 156.97 |
| scaffold1182770.1_Spipo2G0059900 | 0.40 | 0.12 | 3.24 | 4.51E-29 | 2163 | 695.95 | 1467.05 |
| scaffold1182825.2_Spipo1G0088600 | 0.58 | 0.35 | 1.68 | 0.00178 | 450 | 169.21 | 280.79 |
| scaffold1183574.1_Spipo2G0111100 | 0.29 | 0.09 | 3.21 | 2.10E-20 | 1794 | 681.62 | 1112.38 |
| scaffold1186477.3_Spipo13G0026200 | 0.26 | 0.05 | 5.00 | 1.64E-09 | 534 | 221.53 | 312.47 |
| scaffold1187185.1_Spipo3G0038700 | 0.32 | 0.17 | 1.93 | 1.91E-06 | 1257 | 347.74 | 909.26 |
| scaffold1188029.1_Spipo23G0005700 | 0.37 | 0.09 | 4.03 | 1.92E-19 | 1323 | 436.98 | 886.02 |
| scaffold1189610.1_Spipo14G0020700 | 0.38 | 0.15 | 2.60 | 4.19E-12 | 1179 | 378.30 | 800.70 |
| scaffold1190414.1_Spipo8G0029500 | 0.29 | 0.10 | 2.88 | 9.40E-09 | 903 | 280.26 | 622.74 |
| scaffold1190965.1_Spipo14G0012000 | 0.31 | 0.10 | 3.28 | 2.51E-05 | 348 | 129.49 | 218.51 |
| scaffold1191517.1_Spipo21G0023200 | 0.41 | 0.18 | 2.31 | 1.83E-09 | 864 | 334.88 | 529.13 |
| scaffold1193842.1_Spipo8G0034100 | 0.53 | 0.04 | 13.11 | 8.39E-26 | 684 | 300.70 | 383.30 |
| scaffold1193986.1_Spipo15G0043400 | 0.17 | 0.04 | 4.39 | 5.72E-06 | 654 | 182.89 | 471.11 |
| scaffold1194826.1_Spipo9G0067400 | 0.92 | 0.32 | 2.87 | 0.0073943 | 141 | 27.40 | 113.60 |
| scaffold119590.1_Spipo29G0017300 | 0.32 | 0.03 | 9.56 | 1.47E-13 | 771 | 206.15 | 564.85 |
| scaffold1198205.1_Spipo6G0009500 | 0.59 | 0.23 | 2.55 | 8.12E-16 | 1119 | 351.35 | 767.65 |
| scaffold1198849.1_Spipo12G0008200 | 0.50 | 0.31 | 1.60 | 0.0001005 | 828 | 297.89 | 530.12 |
| scaffold120383.1_Spipo0G0155000 | 0.57 | 0.20 | 2.82 | 5.62E-26 | 1482 | 552.69 | 929.31 |
| scaffold1207288.1_Spipo4G0092400 | 0.43 | 0.08 | 5.29 | 3.82E-18 | 849 | 257.59 | 591.42 |
| scaffold1207288.1_Spipo4G0092500 | 0.38 | 0.09 | 4.31 | 3.50E-16 | 1029 | 289.65 | 739.35 |
| scaffold1210159.1_Spipo23G0004200 | 0.39 | 0.20 | 1.99 | 2.84E-06 | 762 | 277.50 | 484.50 |
| scaffold1217442.1_Spipo9G0043600 | 1.36 | 0.61 | 2.22 | 9.29E-07 | 252 | 120.26 | 131.74 |
| scaffold1217442.1_Spipo9G0043600 | 0.88 | 0.27 | 3.29 | 5.67E-05 | 204 | 59.40 | 144.60 |
| scaffold1220093.2_Spipo17G0028000 | 0.27 | 0.15 | 1.81 | 0.003671 | 564 | 180.03 | 383.97 |
| scaffold1220340.1_Spipo5G0018400 | 0.31 | 0.04 | 7.13 | 1.23E-25 | 1434 | 507.00 | 927.00 |
| scaffold1224935.1_Spipo26G0001900 | 0.42 | 0.23 | 1.78 | 0.0003113 | 531 | 193.95 | 337.05 |
| scaffold1226099.1_Spipo28G0004300 | 0.33 | 0.05 | 6.13 | 1.00E-10 | 453 | 182.02 | 270.98 |
| scaffold1227806.1_Spipo13G0011400 | 0.47 | 0.16 | 3.00 | 5.73E-14 | 999 | 279.66 | 719.34 |
| scaffold1229028.2_Spipo22G0026600 | 0.30 | 0.13 | 2.31 | 0.0003091 | 498 | 154.36 | 343.65 |
| scaffold1229458.1_Spipo2G0070200 | 0.46 | 0.14 | 3.21 | 7.06E-09 | 567 | 181.18 | 385.82 |
| scaffold1230588.1_Spipo1G0106300 | 0.36 | 0.21 | 1.70 | 0.0099853 | 396 | 146.51 | 249.49 |
| scaffold1238326.1_Spipo20G0014500 | 0.29 | 0.03 | 10.32 | 2.53E-14 | 582 | 214.94 | 367.07 |
| scaffold1243830.1_Spipo0G0009900 | 0.23 | 0.09 | 2.61 | 0.0048952 | 264 | 116.25 | 147.75 |
| scaffold1245346.1_Spipo0G0020100 | 0.23 | 0.08 | 2.78 | 1.65E-12 | 1575 | 596.25 | 978.75 |
| scaffold1254404.1_Spipo9G0066900 | 0.29 | 0.14 | 2.11 | 0.0066086 | 381 | 118.22 | 262.78 |
| scaffold1260868.1_Spipo3G0031200 | 0.48 | 0.19 | 2.46 | 3.42E-16 | 1314 | 452.80 | 861.20 |
| scaffold1262372.1_Spipo5G0073300 | 0.38 | 0.17 | 2.18 | 4.96E-08 | 924 | 350.13 | 573.87 |
| scaffold1264929.1_Spipo23G0035700 | 0.39 | 0.11 | 3.58 | 2.75E-08 | 441 | 168.46 | 272.54 |
| scaffold1267646.1_Spipo0G0020100 | 0.23 | 0.08 | 2.99 | 2.95E-09 | 960 | 392.94 | 567.06 |
| scaffold1268036.1_Spipo3G0061500 | 0.33 | 0.17 | 1.96 | 0.0015084 | 405 | 151.48 | 253.52 |
| scaffold1268840.1_Spipo15G0040900 | 0.38 | 0.13 | 3.00 | 3.51E-05 | 327 | 108.80 | 218.20 |
| scaffold1275612.1_Spipo11G0063800 | 0.35 | 0.14 | 2.52 | 5.95E-07 | 855 | 200.30 | 654.70 |
| scaffold1275785.1_Spipo26G0025100 | 0.96 | 0.26 | 3.74 | 0.0017307 | 132 | 29.70 | 102.30 |
| scaffold127730.1_Spipo2G0084400 | 0.28 | 0.12 | 2.24 | 0.0037016 | 477 | 116.85 | 360.16 |
| scaffold1277718.1_Spipo7G0037100 | 0.23 | 0.09 | 2.58 | 1.16E-05 | 912 | 231.16 | 680.84 |
| scaffold1279702.1_Spipo21G0006000 | 0.38 | 0.13 | 3.00 | 1.37E-08 | 558 | 229.79 | 328.21 |
| scaffold1280205.1_Spipo8G0052400 | 0.41 | 0.22 | 1.84 | 2.52E-14 | 2442 | 818.84 | 1623.16 |
| scaffold1282171.1_Spipo1G0044700 | 0.72 | 0.31 | 2.33 | 0.0001314 | 204 | 73.87 | 130.13 |
| scaffold1282474.2_Spipo17G0000100 | 0.33 | 0.18 | 1.83 | 0.0006656 | 687 | 222.70 | 464.31 |
| scaffold1286395.2_Spipo0G0031000 | 0.40 | 0.11 | 3.64 | 4.68E-14 | 768 | 302.12 | 465.88 |
| scaffold128684.2_Spipo5G0014400 | 0.56 | 0.09 | 6.60 | 0.0023283 | 114 | 29.96 | 84.04 |
| scaffold1287452.1_Spipo4G0052400 | 0.37 | 0.20 | 1.84 | 7.86E-05 | 807 | 256.27 | 550.73 |
| scaffold1288192.1_Spipo3G0036700 | 0.22 | 0.07 | 3.32 | 0.0012137 | 393 | 92.65 | 300.35 |
| scaffold1288871.1_Spipo10G0009400 | 0.27 | 0.10 | 2.59 | 0.0010682 | 321 | 113.17 | 207.83 |
| scaffold1289403.1_Spipo9G0022200 | 0.59 | 0.15 | 3.96 | 1.12E-11 | 516 | 150.53 | 365.47 |
| scaffold1289634.1_Spipo22G0043600 | 0.49 | 0.15 | 3.23 | 6.85E-12 | 612 | 225.40 | 386.60 |
| scaffold1289634.1_Spipo23G0004200 | 0.45 | 0.18 | 2.45 | 1.17E-07 | 570 | 206.65 | 363.35 |
| scaffold1292096.1_Spipo4G0052400 | 0.36 | 0.20 | 1.83 | 7.80E-05 | 807 | 257.03 | 549.97 |
| scaffold1292949.1_Spipo0G0052100 | 0.41 | 0.17 | 2.38 | 0.0026942 | 186 | 87.08 | 98.92 |
| scaffold1292949.1_Spipo24G0021600 | 0.42 | 0.18 | 2.37 | 1.17E-06 | 609 | 195.79 | 413.21 |
| scaffold1296490.1_Spipo9G0027000 | 0.64 | 0.29 | 2.23 | 0.0002042 | 237 | 91.48 | 145.52 |
| scaffold1297408.1_Spipo20G0037900 | 0.55 | 0.16 | 3.41 | 6.18E-11 | 594 | 150.46 | 443.54 |
| scaffold1299216.1_Spipo9G0017000 | 0.44 | 0.15 | 2.99 | 5.77E-28 | 2271 | 646.86 | 1624.14 |
| scaffold1299216.1_Spipo9G0017000 | 0.46 | 0.08 | 5.96 | 6.15E-05 | 162 | 56.92 | 105.08 |
| scaffold1300882.1_Spipo0G0093300 | 0.33 | 0.16 | 2.11 | 0.003989 | 363 | 97.22 | 265.78 |
| scaffold1301559.1_Spipo8G0034100 | 0.34 | 0.03 | 11.17 | 7.25E-08 | 312 | 103.52 | 208.48 |
| scaffold1302096.1_Spipo16G0029700 | 0.54 | 0.16 | 3.40 | 7.76E-06 | 330 | 99.92 | 230.08 |
| scaffold1302886.1_Spipo1G0013100 | 0.46 | 0.19 | 2.48 | 3.18E-07 | 471 | 210.34 | 260.66 |
| scaffold1303658.1_Spipo9G0040300 | 0.27 | 0.07 | 3.96 | 0.0002384 | 318 | 93.32 | 224.68 |
| scaffold1304698.1_Spipo23G0023300 | 0.31 | 0.15 | 2.13 | 0.0001888 | 501 | 176.35 | 324.65 |
| scaffold1305205.1_Spipo16G0034300 | 0.31 | 0.09 | 3.54 | 8.34E-05 | 273 | 114.15 | 158.85 |
| scaffold1306079.1_Spipo7G0000600 | 0.34 | 0.10 | 3.30 | 6.12E-08 | 555 | 194.76 | 360.24 |
| scaffold130858.1_Spipo10G0014000 | 0.84 | 0.52 | 1.61 | 0.0002882 | 432 | 115.19 | 316.81 |
| scaffold1309041.1_Spipo9G0041500 | 0.45 | 0.12 | 3.67 | 0.0007428 | 171 | 57.45 | 113.55 |
| scaffold1309554.1_Spipo1G0098400 | 0.31 | 0.12 | 2.55 | 1.49E-08 | 921 | 318.15 | 602.86 |
| scaffold1309734.1_Spipo9G0052400 | 0.37 | 0.13 | 2.77 | 1.23E-22 | 2151 | 700.23 | 1450.77 |
| scaffold131029.1_Spipo1G0108000 | 0.66 | 0.10 | 6.54 | 0.0014831 | 132 | 34.07 | 97.93 |
| scaffold131029.1_Spipo1G0108000 | 0.66 | 0.10 | 6.54 | 0.0014831 | 132 | 34.07 | 97.93 |
| scaffold131029.1_Spipo1G0108000 | 0.66 | 0.10 | 6.54 | 0.0014831 | 132 | 34.07 | 97.93 |
| scaffold131029.2_Spipo1G0108000 | 0.22 | 0.05 | 4.13 | 0.0002491 | 366 | 107.80 | 258.20 |
| scaffold1310995.1_Spipo25G0016000 | 0.51 | 0.19 | 2.62 | 2.01E-24 | 1806 | 654.97 | 1151.03 |
| scaffold1311149.2_Spipo9G0010800 | 0.43 | 0.12 | 3.51 | 1.80E-13 | 747 | 265.05 | 481.95 |
| scaffold1311149.2_Spipo9G0011000 | 0.43 | 0.12 | 3.49 | 2.68E-13 | 753 | 270.84 | 482.16 |
| scaffold1311205.1_Spipo6G0077300 | 0.32 | 0.18 | 1.77 | 0.0012901 | 711 | 210.99 | 500.01 |
| scaffold1315833.2_Spipo5G0052300 | 0.50 | 0.23 | 2.18 | 9.76E-16 | 1458 | 523.22 | 934.78 |
| scaffold1320272.1_Spipo22G0043600 | 0.48 | 0.12 | 4.13 | 8.23E-13 | 522 | 194.38 | 327.62 |
| scaffold1320272.1_Spipo23G0004200 | 0.42 | 0.19 | 2.14 | 0.0004964 | 345 | 131.87 | 213.13 |
| scaffold1321802.1_Spipo24G0014800 | 0.36 | 0.10 | 3.68 | 2.72E-10 | 789 | 230.29 | 558.71 |
| scaffold1324923.1_Spipo1G0036100 | 0.41 | 0.26 | 1.59 | 0.0003814 | 933 | 296.85 | 636.15 |
| scaffold1325783.1_Spipo2G0106600 | 0.27 | 0.10 | 2.69 | 8.96E-06 | 720 | 189.28 | 530.73 |
| scaffold1326362.1_Spipo12G0009200 | 0.27 | 0.08 | 3.48 | 0.0002604 | 399 | 102.90 | 296.10 |
| scaffold1327229.1_Spipo8G0072000 | 0.59 | 0.30 | 2.00 | 2.93E-13 | 1359 | 411.23 | 947.77 |
| scaffold1327966.1_Spipo27G0017500 | 0.62 | 0.23 | 2.69 | 9.02E-08 | 375 | 148.47 | 226.53 |
| scaffold1330901.1_Spipo22G0030600 | 0.38 | 0.13 | 2.87 | 6.38E-23 | 2142 | 662.99 | 1479.01 |
| scaffold1332154.1_Spipo6G0075100 | 0.27 | 0.07 | 3.99 | 1.61E-19 | 1416 | 567.42 | 848.58 |
| scaffold1332348.1_Spipo31G0004500 | 0.51 | 0.24 | 2.16 | 5.05E-16 | 1656 | 511.64 | 1144.36 |
| scaffold1336620.2_Spipo11G0042100 | 0.50 | 0.32 | 1.56 | 0.0001794 | 768 | 281.26 | 486.74 |
| scaffold1336620.2_Spipo11G0042100 | 0.48 | 0.15 | 3.13 | 0.0001079 | 183 | 79.19 | 103.81 |
| scaffold1337493.1_Spipo2G0065000 | 1.00 | 0.45 | 2.23 | 3.39E-07 | 369 | 152.20 | 216.80 |
| scaffold1338289.2_Spipo5G0012100 | 0.28 | 0.07 | 3.75 | 4.81E-10 | 795 | 270.29 | 524.72 |
| scaffold1339015.1_Spipo22G0043600 | 0.60 | 0.07 | 8.45 | 1.07E-12 | 303 | 110.26 | 192.74 |
| scaffold1341631.1_Spipo14G0034500 | 0.58 | 0.23 | 2.50 | 0.0044472 | 159 | 49.10 | 109.90 |
| scaffold1345008.1_Spipo7G0013600 | 0.45 | 0.13 | 3.45 | 0.0088402 | 72 | 31.77 | 40.23 |
| scaffold1350395.1_Spipo13G0049000 | 0.29 | 0.02 | 16.53 | 1.07E-15 | 468 | 237.19 | 230.82 |
| scaffold1351691.1_Spipo6G0070600 | 0.19 | 0.04 | 4.49 | 9.55E-05 | 372 | 140.71 | 231.30 |
| scaffold1352371.1_Spipo14G0013600 | 0.23 | 0.02 | 9.31 | 0.0003336 | 264 | 64.80 | 199.21 |
| scaffold1353000.1_Spipo3G0102600 | 0.27 | 0.06 | 4.74 | 1.15E-08 | 537 | 183.38 | 353.62 |
| scaffold1357560.1_Spipo3G0109100 | 0.34 | 0.16 | 2.15 | 0.0007501 | 399 | 139.73 | 259.27 |
| scaffold1358543.1_Spipo7G0040200 | 0.37 | 0.14 | 2.64 | 0.0005402 | 315 | 91.13 | 223.87 |
| scaffold1358646.1_Spipo0G0079300 | 0.34 | 0.09 | 3.77 | 5.15E-06 | 339 | 127.34 | 211.67 |
| scaffold1358668.1_Spipo4G0053000 | 0.46 | 0.18 | 2.48 | 1.14E-05 | 366 | 145.51 | 220.49 |
| scaffold1358906.1_Spipo16G0023000 | 0.29 | 0.02 | 15.50 | 2.62E-17 | 774 | 226.18 | 547.82 |
| scaffold1359248.1_Spipo3G0098500 | 0.27 | 0.06 | 4.15 | 3.87E-12 | 1041 | 325.90 | 715.10 |
| scaffold1360601.2_Spipo9G0055800 | 0.38 | 0.13 | 2.92 | 8.81E-06 | 405 | 118.84 | 286.16 |
| scaffold136079.1_Spipo21G0025400 | 0.35 | 0.17 | 2.07 | 3.37E-10 | 1839 | 484.31 | 1354.69 |
| scaffold1364135.1_Spipo2G0081800 | 0.39 | 0.13 | 3.15 | 1.77E-14 | 1341 | 314.21 | 1026.79 |
| scaffold1364138.1_Spipo12G0014100 | 0.31 | 0.19 | 1.63 | 5.22E-05 | 1356 | 460.52 | 895.48 |
| scaffold1365587.1_Spipo1G0110800 | 0.25 | 0.11 | 2.36 | 0.0006713 | 510 | 152.26 | 357.74 |
| scaffold1368166.1_Spipo1G0021400 | 0.31 | 0.13 | 2.34 | 0.003579 | 297 | 105.18 | 191.82 |
| scaffold1368568.1_Spipo10G0020900 | 0.49 | 0.19 | 2.64 | 0.0004392 | 219 | 74.44 | 144.56 |
| scaffold1369529.1_Spipo15G0034400 | 0.64 | 0.29 | 2.19 | 0.0081396 | 123 | 44.18 | 78.82 |
| scaffold137179.1_Spipo20G0010700 | 0.54 | 0.12 | 4.42 | 1.22E-17 | 639 | 257.96 | 381.04 |
| scaffold137179.1_Spipo9G0026100 | 0.52 | 0.11 | 4.59 | 1.17E-18 | 636 | 273.56 | 362.44 |
| scaffold1373724.1_Spipo24G0030900 | 0.17 | 0.04 | 4.12 | 0.0001335 | 378 | 161.52 | 216.48 |
| scaffold1375487.1_Spipo3G0076300 | 0.25 | 0.06 | 3.98 | 7.23E-11 | 936 | 299.42 | 636.58 |
| scaffold1380456.1_Spipo10G0036300 | 0.37 | 0.17 | 2.19 | 2.06E-07 | 1035 | 266.38 | 768.62 |
| scaffold1385078.1_Spipo22G0028400 | 0.42 | 0.12 | 3.49 | 5.71E-17 | 969 | 347.06 | 621.94 |
| scaffold1385093.1_Spipo11G0040100 | 1.37 | 0.04 | 38.19 | 1.62E-09 | 114 | 47.13 | 66.87 |
| scaffold1387915.1_Spipo0G0179300 | 0.40 | 0.24 | 1.66 | 0.0006237 | 624 | 227.32 | 396.68 |
| scaffold1388651.1_Spipo10G0046000 | 0.36 | 0.19 | 1.90 | 4.15E-05 | 792 | 250.22 | 541.78 |
| scaffold1388963.1_Spipo2G0057800 | 0.24 | 0.15 | 1.62 | 0.0006247 | 1227 | 424.94 | 802.06 |
| scaffold1389695.1_Spipo13G0038700 | 0.23 | 0.09 | 2.50 | 2.25E-05 | 762 | 236.18 | 525.83 |
| scaffold1390649.1_Spipo1G0014600 | 0.32 | 0.12 | 2.70 | 3.48E-14 | 1527 | 497.54 | 1029.46 |
| scaffold1391417.1_Spipo22G0037100 | 0.33 | 0.04 | 7.84 | 4.13E-08 | 315 | 121.47 | 193.53 |
| scaffold139199.1_Spipo9G0069300 | 0.26 | 0.11 | 2.22 | 0.002534 | 426 | 137.68 | 288.32 |
| scaffold1392830.2_Spipo10G0053600 | 0.39 | 0.16 | 2.49 | 2.49E-10 | 1164 | 327.74 | 836.26 |
| scaffold1393387.1_Spipo21G0039100 | 0.39 | 0.10 | 3.72 | 5.94E-06 | 300 | 121.72 | 178.28 |
| scaffold1393564.1_Spipo29G0000700 | 0.26 | 0.13 | 2.06 | 0.0001704 | 690 | 250.71 | 439.29 |
| scaffold1396014.1_Spipo0G0008600 | 0.39 | 0.14 | 2.70 | 6.71E-13 | 1122 | 365.47 | 756.53 |
| scaffold1397734.1_Spipo7G0015100 | 0.48 | 0.24 | 2.00 | 4.50E-10 | 1236 | 382.70 | 853.30 |
| scaffold1398610.1_Spipo1G0036400 | 0.26 | 0.04 | 6.55 | 9.93E-13 | 1131 | 278.11 | 852.89 |
| scaffold1402012.1_Spipo4G0071200 | 0.42 | 0.17 | 2.42 | 0.0072354 | 207 | 63.21 | 143.79 |
| scaffold1402660.1_Spipo2G0124500 | 0.34 | 0.09 | 3.63 | 0.0043536 | 183 | 57.19 | 125.81 |
| scaffold1405313.1_Spipo11G0051500 | 0.26 | 0.15 | 1.72 | 8.64E-05 | 1164 | 477.21 | 686.79 |
| scaffold1405620.2_Spipo7G0053900 | 0.45 | 0.15 | 2.96 | 9.21E-08 | 471 | 161.35 | 309.65 |
| scaffold1405981.1_Spipo20G0023400 | 0.24 | 0.00 | 50.00 | 5.13E-05 | 222 | 57.17 | 164.83 |
| scaffold1406395.3_Spipo1G0046300 | 0.19 | 0.05 | 3.62 | 0.0005773 | 321 | 124.30 | 196.70 |
| scaffold1406706.1_Spipo0G0123000 | 0.26 | 0.06 | 4.06 | 5.49E-09 | 774 | 232.20 | 541.80 |
| scaffold1406706.1_Spipo2G0061200 | 0.44 | 0.08 | 5.81 | 4.32E-18 | 771 | 276.56 | 494.45 |
| scaffold1406859.1_Spipo0G0172000 | 0.65 | 0.44 | 1.50 | 3.55E-05 | 921 | 307.73 | 613.27 |
| scaffold1407003.1_Spipo21G0002200 | 0.29 | 0.07 | 4.14 | 1.54E-05 | 384 | 109.82 | 274.18 |
| scaffold1407958.1_Spipo19G0005300 | 0.39 | 0.12 | 3.16 | 4.84E-31 | 2373 | 785.68 | 1587.32 |
| scaffold1408634.1_Spipo22G0026300 | 0.47 | 0.15 | 3.06 | 1.19E-06 | 339 | 120.64 | 218.36 |
| scaffold1409464.1_Spipo27G0017500 | 0.36 | 0.19 | 1.86 | 2.37E-07 | 1218 | 424.41 | 793.59 |
| scaffold1409464.1_Spipo2G0021800 | 0.69 | 0.34 | 2.01 | 2.23E-14 | 1125 | 424.04 | 700.96 |
| scaffold1411155.1_Spipo0G0003500 | 0.34 | 0.19 | 1.79 | 0.000103 | 780 | 297.14 | 482.87 |
| scaffold1412037.1_Spipo17G0013900 | 0.37 | 0.09 | 4.32 | 0.0004505 | 162 | 68.03 | 93.97 |
| scaffold1412132.1_Spipo6G0034300 | 0.72 | 0.38 | 1.89 | 7.22E-05 | 528 | 140.05 | 387.95 |
| scaffold1412661.1_Spipo0G0167100 | 0.55 | 0.31 | 1.77 | 6.81E-08 | 1116 | 364.36 | 751.64 |
| scaffold1413514.1_Spipo22G0015000 | 0.43 | 0.06 | 7.58 | 1.86E-07 | 291 | 89.89 | 201.11 |
| scaffold1413515.1_Spipo22G0015000 | 0.43 | 0.06 | 7.58 | 1.86E-07 | 291 | 89.89 | 201.11 |
| scaffold1415622.1_Spipo17G0019200 | 0.38 | 0.16 | 2.47 | 1.21E-11 | 1143 | 390.12 | 752.89 |
| scaffold1416963.1_Spipo27G0020300 | 0.39 | 0.18 | 2.20 | 7.30E-23 | 2934 | 904.42 | 2029.58 |
| scaffold1417095.1_Spipo0G0065600 | 0.51 | 0.23 | 2.18 | 0.0013555 | 231 | 85.38 | 145.62 |
| scaffold1418164.1_Spipo23G0029800 | 0.62 | 0.10 | 6.20 | 1.31E-08 | 204 | 87.75 | 116.25 |
| scaffold1420752.1_Spipo14G0017200 | 0.40 | 0.29 | 1.36 | 0.0046008 | 1179 | 361.59 | 817.41 |
| scaffold1423620.1_Spipo13G0019000 | 0.21 | 0.05 | 4.01 | 1.26E-05 | 567 | 171.13 | 395.87 |
| scaffold1423963.1_Spipo12G0015600 | 0.63 | 0.47 | 1.35 | 0.0051757 | 777 | 247.62 | 529.38 |
| scaffold1423963.1_Spipo16G0043900 | 0.69 | 0.45 | 1.56 | 1.61E-06 | 966 | 346.70 | 619.30 |
| scaffold1423963.1_Spipo16G0044100 | 0.65 | 0.48 | 1.36 | 0.0012933 | 957 | 314.42 | 642.58 |
| scaffold1434368.1_Spipo21G0029700 | 0.28 | 0.15 | 1.91 | 0.0034291 | 498 | 164.45 | 333.56 |
| scaffold1434780.1_Spipo10G0001200 | 0.30 | 0.17 | 1.71 | 7.26E-05 | 1161 | 381.18 | 779.82 |
| scaffold1435237.1_Spipo3G0005600 | 0.40 | 0.11 | 3.54 | 3.10E-32 | 2280 | 718.96 | 1561.04 |
| scaffold1436318.1_Spipo12G0015200 | 0.34 | 0.18 | 1.93 | 6.46E-06 | 924 | 297.72 | 626.28 |
| scaffold1440664.1_Spipo28G0003700 | 0.53 | 0.07 | 7.88 | 4.41E-23 | 657 | 233.12 | 423.88 |
| scaffold1441064.1_Spipo7G0015100 | 0.31 | 0.11 | 2.73 | 3.98E-14 | 1347 | 527.09 | 819.91 |
| scaffold1443119.1_Spipo12G0059000 | 0.23 | 0.12 | 1.97 | 0.0003988 | 810 | 289.09 | 520.91 |
| scaffold1444076.1_Spipo11G0061700 | 0.27 | 0.11 | 2.50 | 4.74E-07 | 867 | 295.49 | 571.51 |
| scaffold1445344.1_Spipo21G0028000 | 0.44 | 0.22 | 2.00 | 1.02E-07 | 930 | 297.07 | 632.93 |
| scaffold1447045.1_Spipo3G0031400 | 0.60 | 0.26 | 2.28 | 0.0002466 | 306 | 92.34 | 213.66 |
| scaffold1451279.1_Spipo26G0022000 | 0.34 | 0.10 | 3.44 | 5.47E-12 | 885 | 291.29 | 593.71 |
| scaffold1453988.1_Spipo26G0001200 | 0.53 | 0.30 | 1.76 | 6.76E-05 | 705 | 249.82 | 455.18 |
| scaffold1455200.1_Spipo29G0027800 | 0.35 | 0.15 | 2.38 | 3.19E-12 | 1674 | 443.79 | 1230.21 |
| scaffold1455547.1_Spipo8G0070000 | 0.49 | 0.22 | 2.20 | 4.52E-11 | 996 | 369.07 | 626.93 |
| scaffold1455747.1_Spipo29G0009700 | 0.21 | 0.04 | 5.15 | 0.0085891 | 159 | 47.87 | 111.13 |
| scaffold1456750.2_Spipo9G0043300 | 0.47 | 0.35 | 1.35 | 1.86E-06 | 2790 | 1019.23 | 1770.77 |
| scaffold145798.1_Spipo19G0004600 | 0.43 | 0.14 | 3.19 | 0.0084835 | 132 | 41.45 | 90.55 |
| scaffold1458807.1_Spipo4G0073300 | 0.22 | 0.06 | 3.80 | 9.86E-05 | 471 | 131.53 | 339.47 |
| scaffold1460365.1_Spipo14G0025100 | 0.40 | 0.02 | 22.01 | 1.50E-14 | 423 | 144.41 | 278.59 |
| scaffold1460773.1_Spipo13G0015200 | 0.31 | 0.03 | 10.85 | 8.88E-06 | 321 | 90.96 | 230.04 |
| scaffold146211.1_Spipo5G0000600 | 0.18 | 0.06 | 3.04 | 0.0005953 | 564 | 158.91 | 405.09 |
| scaffold1463472.1_Spipo4G0067400 | 1.17 | 0.41 | 2.88 | 2.40E-13 | 432 | 159.20 | 272.80 |
| scaffold1463899.1_Spipo0G0188200 | 0.39 | 0.21 | 1.89 | 1.40E-05 | 804 | 287.46 | 516.54 |
| scaffold1463899.1_Spipo22G0027500 | 0.38 | 0.21 | 1.80 | 5.93E-05 | 795 | 281.83 | 513.17 |
| scaffold1470298.1_Spipo1G0094000 | 0.27 | 0.04 | 6.68 | 7.88E-08 | 318 | 138.16 | 179.84 |
| scaffold1473121.1_Spipo1G0012300 | 0.48 | 0.13 | 3.69 | 2.68E-14 | 714 | 252.61 | 461.39 |
| scaffold1475338.1_Spipo0G0080100 | 0.37 | 0.09 | 3.98 | 2.05E-14 | 816 | 315.92 | 500.08 |
| scaffold1475612.1_Spipo16G0001500 | 0.44 | 0.16 | 2.75 | 2.62E-09 | 621 | 233.57 | 387.43 |
| scaffold1476416.1_Spipo1G0110000 | 0.43 | 0.21 | 2.09 | 3.67E-15 | 1683 | 642.73 | 1040.27 |
| scaffold1478536.1_Spipo1G0063400 | 0.59 | 0.23 | 2.59 | 0.0012004 | 177 | 53.48 | 123.52 |
| scaffold1480541.1_Spipo17G0045500 | 0.24 | 0.02 | 11.78 | 6.47E-08 | 462 | 165.62 | 296.38 |
| scaffold1480577.1_Spipo2G0108000 | 0.40 | 0.10 | 4.04 | 3.11E-08 | 369 | 149.55 | 219.45 |
| scaffold1483149.1_Spipo3G0027600 | 0.29 | 0.13 | 2.30 | 0.0079398 | 300 | 79.03 | 220.97 |
| scaffold1485581.1_Spipo9G0041300 | 0.34 | 0.15 | 2.34 | 5.69E-10 | 1209 | 381.93 | 827.07 |
| scaffold1485641.1_Spipo29G0004400 | 0.43 | 0.17 | 2.59 | 1.07E-14 | 1323 | 395.04 | 927.96 |
| scaffold1488116.1_Spipo4G0027800 | 0.42 | 0.28 | 1.47 | 0.0022669 | 882 | 267.12 | 614.88 |
| scaffold1490302.1_Spipo19G0029900 | 0.18 | 0.02 | 9.47 | 0.0016729 | 135 | 51.09 | 83.91 |
| scaffold1491527.1_Spipo26G0005100 | 0.19 | 0.03 | 5.75 | 1.49E-07 | 723 | 191.11 | 531.89 |
| scaffold1491664.1_Spipo4G0011500 | 0.30 | 0.07 | 4.25 | 0.0018632 | 189 | 57.19 | 131.81 |
| scaffold1495429.1_Spipo3G0025800 | 0.23 | 0.04 | 5.69 | 0.0005236 | 207 | 76.30 | 130.70 |
| scaffold1497036.1_Spipo15G0019600 | 0.28 | 0.03 | 8.40 | 2.23E-05 | 234 | 72.02 | 161.98 |
| scaffold1497111.1_Spipo6G0076600 | 0.32 | 0.16 | 2.01 | 0.0006218 | 516 | 185.35 | 330.65 |
| scaffold1497587.2_Spipo16G0049100 | 0.26 | 0.05 | 5.70 | 0.0013832 | 150 | 48.48 | 101.52 |
| scaffold1500896.1_Spipo13G0034300 | 0.86 | 0.02 | 50.00 | 3.59E-23 | 291 | 133.24 | 157.76 |
| scaffold1501154.1_Spipo28G0008300 | 0.55 | 0.23 | 2.41 | 2.61E-15 | 1182 | 398.60 | 783.40 |
| scaffold1501177.1_Spipo3G0108500 | 0.26 | 0.14 | 1.88 | 3.57E-06 | 1380 | 476.24 | 903.76 |
| scaffold1501208.1_Spipo0G0102300 | 0.30 | 0.10 | 3.06 | 5.47E-15 | 1287 | 539.89 | 747.11 |
| scaffold1501560.2_Spipo23G0028300 | 0.33 | 0.18 | 1.88 | 4.15E-11 | 2007 | 771.10 | 1235.90 |
| scaffold1501862.1_Spipo9G0025800 | 0.39 | 0.20 | 1.92 | 0.0001441 | 618 | 206.51 | 411.49 |
| scaffold1502037.1_Spipo0G0126300 | 0.35 | 0.10 | 3.68 | 0.0002378 | 240 | 73.55 | 166.45 |
| scaffold1502037.1_Spipo2G0118200 | 0.34 | 0.08 | 4.30 | 0.0027203 | 138 | 52.94 | 85.06 |
| scaffold152979.1_Spipo22G0011600 | 0.36 | 0.16 | 2.26 | 2.61E-07 | 897 | 253.98 | 643.02 |
| scaffold153097.1_Spipo14G0001700 | 0.52 | 0.24 | 2.14 | 3.16E-14 | 1419 | 464.13 | 954.87 |
| scaffold153097.1_Spipo2G0058800 | 0.45 | 0.33 | 1.35 | 0.0009321 | 1401 | 517.98 | 883.02 |
| scaffold153226.2_Spipo24G0014100 | 0.28 | 0.02 | 18.62 | 4.03E-05 | 177 | 54.78 | 122.22 |
| scaffold156252.1_Spipo3G0035900 | 0.38 | 0.08 | 4.69 | 5.56E-31 | 1938 | 557.79 | 1380.21 |
| scaffold169769.1_Spipo8G0008800 | 0.22 | 0.05 | 4.70 | 6.35E-13 | 1230 | 356.97 | 873.03 |
| scaffold172327.1_Spipo2G0032500 | 0.55 | 0.36 | 1.53 | 0.0022496 | 516 | 180.40 | 335.60 |
| scaffold175368.1_Spipo18G0017800 | 0.31 | 0.14 | 2.30 | 5.12E-14 | 1992 | 642.57 | 1349.43 |
| scaffold177408.2_Spipo2G0058100 | 0.24 | 0.07 | 3.21 | 5.33E-08 | 900 | 275.92 | 624.08 |
| scaffold178116.1_Spipo13G0014100 | 0.52 | 0.28 | 1.85 | 0.0044815 | 252 | 103.76 | 148.24 |
| scaffold183146.1_Spipo8G0024200 | 0.29 | 0.12 | 2.43 | 2.75E-10 | 1416 | 452.21 | 963.79 |
| scaffold183598.1_Spipo8G0048400 | 0.33 | 0.28 | 1.18 | 0.0086334 | 4521 | 1088.72 | 3432.28 |
| scaffold18783.1_Spipo0G0152800 | 0.12 | 0.04 | 2.70 | 0.0082392 | 573 | 169.53 | 403.47 |
| scaffold191426.1_Spipo0G0098700 | 0.46 | 0.05 | 8.96 | 6.70E-12 | 387 | 120.48 | 266.52 |
| scaffold194233.1_Spipo8G0022900 | 0.26 | 0.09 | 2.86 | 4.80E-06 | 744 | 201.30 | 542.70 |
| scaffold197083.1_Spipo23G0029300 | 0.13 | 0.02 | 5.91 | 0.004533 | 294 | 81.64 | 212.36 |
| scaffold206068.1_Spipo23G0002400 | 0.48 | 0.29 | 1.65 | 4.09E-05 | 897 | 285.87 | 611.13 |
| scaffold206813.1_Spipo15G0005500 | 0.21 | 0.07 | 2.85 | 8.68E-08 | 1425 | 324.30 | 1100.70 |
| scaffold210001.1_Spipo4G0051500 | 0.23 | 0.07 | 3.13 | 4.16E-06 | 639 | 207.84 | 431.16 |
| scaffold213470.1_Spipo5G0032400 | 0.18 | 0.02 | 9.05 | 6.28E-20 | 1875 | 485.17 | 1389.83 |
| scaffold214613.1_Spipo9G0018000 | 0.38 | 0.03 | 12.41 | 3.10E-11 | 456 | 123.70 | 332.30 |
| scaffold219375.1_Spipo21G0039100 | 0.38 | 0.09 | 4.10 | 2.01E-06 | 300 | 120.20 | 179.80 |
| scaffold223079.1_Spipo2G0041200 | 0.24 | 0.05 | 5.13 | 8.08E-05 | 303 | 91.49 | 211.52 |
| scaffold223460.1_Spipo13G0022900 | 0.60 | 0.38 | 1.56 | 1.09E-07 | 1584 | 442.12 | 1141.88 |
| scaffold224231.1_Spipo9G0005800 | 0.31 | 0.13 | 2.34 | 2.52E-05 | 534 | 225.58 | 308.42 |
| scaffold224445.2_Spipo2G0083000 | 0.52 | 0.15 | 3.58 | 3.18E-22 | 1140 | 357.71 | 782.29 |
| scaffold225310.1_Spipo3G0018400 | 0.36 | 0.14 | 2.56 | 1.10E-05 | 438 | 167.54 | 270.46 |
| scaffold226899.1_Spipo4G0031600 | 0.41 | 0.12 | 3.38 | 4.24E-15 | 888 | 340.41 | 547.59 |
| scaffold230443.1_Spipo3G0082600 | 0.21 | 0.02 | 9.10 | 1.24E-05 | 390 | 102.30 | 287.71 |
| scaffold230443.1_Spipo3G0082600 | 0.78 | 0.39 | 2.00 | 0.0005737 | 237 | 63.51 | 173.49 |
| scaffold231104.1_Spipo12G0049200 | 0.27 | 0.10 | 2.82 | 5.64E-17 | 2445 | 613.91 | 1831.09 |
| scaffold231281.1_Spipo18G0038300 | 0.24 | 0.05 | 4.46 | 2.38E-10 | 912 | 262.29 | 649.71 |
| scaffold241171.1_Spipo2G0066800 | 0.63 | 0.13 | 4.82 | 2.56E-18 | 714 | 206.03 | 507.97 |
| scaffold243573.1_Spipo0G0018900 | 0.37 | 0.17 | 2.13 | 0.0005803 | 405 | 125.22 | 279.78 |
| scaffold243573.1_Spipo4G0018900 | 0.38 | 0.11 | 3.35 | 4.24E-08 | 522 | 175.26 | 346.74 |
| scaffold24410.1_Spipo1G0036100 | 0.39 | 0.16 | 2.39 | 0.0025877 | 255 | 81.53 | 173.47 |
| scaffold244995.1_Spipo14G0015200 | 0.28 | 0.06 | 4.97 | 2.38E-10 | 795 | 235.35 | 559.65 |
| scaffold248456.1_Spipo19G0025000 | 0.22 | 0.05 | 4.20 | 8.28E-14 | 1350 | 425.78 | 924.22 |
| scaffold248761.1_Spipo0G0170100 | 0.40 | 0.18 | 2.28 | 0.0003088 | 375 | 128.22 | 246.78 |
| scaffold250433.1_Spipo11G0061900 | 0.50 | 0.16 | 3.17 | 4.89E-05 | 246 | 71.70 | 174.30 |
| scaffold252026.1_Spipo29G0024500 | 0.45 | 0.14 | 3.16 | 0.0003948 | 246 | 58.96 | 187.04 |
| scaffold263327.1_Spipo11G0008800 | 0.41 | 0.14 | 2.85 | 4.81E-09 | 669 | 208.97 | 460.03 |
| scaffold273561.1_Spipo19G0004600 | 0.42 | 0.15 | 2.89 | 0.0096287 | 132 | 42.70 | 89.30 |
| scaffold276761.1_Spipo26G0005400 | 0.25 | 0.09 | 2.61 | 8.03E-06 | 906 | 228.61 | 677.39 |
| scaffold277887.1_Spipo28G0023000 | 0.29 | 0.02 | 16.16 | 3.74E-05 | 195 | 51.94 | 143.06 |
| scaffold283309.1_Spipo12G0000500 | 0.21 | 0.05 | 4.03 | 7.80E-10 | 1173 | 294.70 | 878.30 |
| scaffold283828.1_Spipo1G0046300 | 0.33 | 0.13 | 2.45 | 3.39E-12 | 1743 | 465.30 | 1277.70 |
| scaffold285920.1_Spipo14G0003200 | 0.94 | 0.51 | 1.84 | 1.16E-06 | 474 | 173.30 | 300.70 |
| scaffold289082.1_Spipo4G0065100 | 0.25 | 0.10 | 2.39 | 1.98E-07 | 1017 | 372.57 | 644.43 |
| scaffold296763.1_Spipo12G0023700 | 0.25 | 0.13 | 1.94 | 0.0048609 | 576 | 166.02 | 409.98 |
| scaffold296763.1_Spipo5G0018800 | 0.30 | 0.09 | 3.45 | 8.98E-09 | 747 | 222.53 | 524.47 |
| scaffold300736.2_Spipo18G0037100 | 0.31 | 0.11 | 2.68 | 1.06E-17 | 2442 | 606.68 | 1835.32 |
| scaffold305608.1_Spipo12G0049000 | 0.25 | 0.07 | 3.50 | 2.45E-09 | 921 | 282.33 | 638.67 |
| scaffold316273.1_Spipo0G0103300 | 0.61 | 0.40 | 1.51 | 1.55E-12 | 3027 | 833.06 | 2193.94 |
| scaffold316634.1_Spipo19G0013700 | 0.20 | 0.07 | 2.88 | 2.72E-06 | 915 | 274.66 | 640.34 |
| scaffold317170.1_Spipo29G0003100 | 0.32 | 0.11 | 2.95 | 0.0006498 | 345 | 81.58 | 263.42 |
| scaffold320326.2_Spipo15G0034100 | 0.61 | 0.47 | 1.29 | 0.0042146 | 1074 | 344.13 | 729.87 |
| scaffold320688.1_Spipo17G0016800 | 0.29 | 0.06 | 4.85 | 4.00E-13 | 879 | 271.89 | 607.11 |
| scaffold322581.1_Spipo21G0014000 | 0.50 | 0.19 | 2.70 | 0.0010413 | 204 | 59.51 | 144.49 |
| scaffold329089.1_Spipo0G0081500 | 0.44 | 0.11 | 3.88 | 9.23E-11 | 564 | 180.76 | 383.24 |
| scaffold330584.1_Spipo16G0045600 | 0.73 | 0.54 | 1.34 | 0.0071018 | 738 | 234.95 | 503.06 |
| scaffold331865.1_Spipo3G0037000 | 0.27 | 0.02 | 11.42 | 0.0001025 | 258 | 82.77 | 175.23 |
| scaffold332158.1_Spipo23G0023300 | 0.49 | 0.22 | 2.20 | 8.69E-06 | 597 | 158.37 | 438.63 |
| scaffold332199.2_Spipo22G0002100 | 0.24 | 0.06 | 4.11 | 4.26E-06 | 627 | 159.91 | 467.10 |
| scaffold332994.1_Spipo11G0028500 | 0.45 | 0.11 | 3.92 | 2.12E-14 | 876 | 236.51 | 639.49 |
| scaffold333116.1_Spipo10G0048100 | 0.42 | 0.12 | 3.41 | 2.50E-19 | 1278 | 424.96 | 853.04 |
| scaffold333116.1_Spipo4G0036900 | 0.54 | 0.34 | 1.56 | 4.18E-05 | 930 | 283.21 | 646.79 |
| scaffold334658.1_Spipo12G0052800 | 1.51 | 0.18 | 8.45 | 4.52E-09 | 129 | 52.17 | 76.83 |
| scaffold335878.1_Spipo1G0087600 | 0.39 | 0.10 | 3.83 | 9.52E-26 | 1437 | 550.00 | 887.00 |
| scaffold338012.2_Spipo21G0004200 | 0.30 | 0.18 | 1.68 | 0.0005396 | 1038 | 314.44 | 723.56 |
| scaffold338012.5_Spipo21G0004200 | 0.30 | 0.18 | 1.68 | 0.0005396 | 1038 | 314.44 | 723.56 |
| scaffold343498.1_Spipo3G0003100 | 0.21 | 0.05 | 4.06 | 0.0003763 | 378 | 106.25 | 271.75 |
| scaffold351444.1_Spipo4G0078300 | 0.23 | 0.06 | 3.85 | 1.23E-08 | 843 | 250.45 | 592.56 |
| scaffold353409.1_Spipo32G0003600 | 0.41 | 0.14 | 2.83 | 4.99E-19 | 1419 | 524.88 | 894.12 |
| scaffold355842.1_Spipo15G0029600 | 0.25 | 0.16 | 1.59 | 0.0002653 | 1407 | 546.82 | 860.18 |
| scaffold355863.1_Spipo4G0047000 | 0.38 | 0.17 | 2.18 | 0.0002363 | 453 | 122.62 | 330.38 |
| scaffold356239.1_Spipo23G0025000 | 0.51 | 0.28 | 1.81 | 5.04E-05 | 666 | 251.11 | 414.89 |
| scaffold35870.1_Spipo0G0102100 | 0.41 | 0.17 | 2.39 | 4.12E-17 | 1908 | 543.03 | 1364.98 |
| scaffold360098.1_Spipo6G0041900 | 0.41 | 0.23 | 1.81 | 5.00E-10 | 1893 | 576.18 | 1316.82 |
| scaffold364012.1_Spipo0G0014900 | 0.59 | 0.25 | 2.33 | 0.0068574 | 138 | 48.17 | 89.83 |
| scaffold364046.1_Spipo27G0020200 | 0.42 | 0.06 | 7.15 | 7.25E-08 | 243 | 86.24 | 156.76 |
| scaffold368747.1_Spipo3G0031200 | 0.61 | 0.28 | 2.18 | 0.0002431 | 240 | 101.73 | 138.27 |
| scaffold369558.1_Spipo6G0010300 | 0.55 | 0.28 | 1.95 | 5.87E-09 | 903 | 316.09 | 586.91 |
| scaffold372752.1_Spipo0G0066600 | 0.46 | 0.15 | 2.98 | 1.09E-10 | 630 | 236.30 | 393.70 |
| scaffold372752.1_Spipo0G0066800 | 0.39 | 0.12 | 3.34 | 1.48E-21 | 1374 | 527.99 | 846.01 |
| scaffold373656.1_Spipo13G0030000 | 0.84 | 0.32 | 2.60 | 1.54E-07 | 312 | 101.94 | 210.06 |
| scaffold375640.1_Spipo13G0001400 | 0.44 | 0.08 | 5.42 | 5.03E-14 | 501 | 207.39 | 293.61 |
| scaffold376520.1_Spipo26G0021900 | 0.15 | 0.00 | 50.00 | 0.0010711 | 183 | 58.52 | 124.48 |
| scaffold376766.1_Spipo5G0034900 | 0.34 | 0.20 | 1.75 | 0.001018 | 804 | 201.88 | 602.12 |
| scaffold377219.1_Spipo23G0002400 | 0.50 | 0.34 | 1.47 | 0.000254 | 1047 | 333.97 | 713.03 |
| scaffold381068.1_Spipo16G0016600 | 0.24 | 0.09 | 2.68 | 5.67E-25 | 4533 | 1131.55 | 3401.45 |
| scaffold388101.1_Spipo1G0066700 | 0.23 | 0.04 | 6.34 | 9.55E-08 | 657 | 153.38 | 503.62 |
| scaffold389718.1_Spipo28G0026000 | 0.42 | 0.12 | 3.51 | 9.92E-10 | 573 | 194.52 | 378.49 |
| scaffold392992.1_Spipo0G0072800 | 0.46 | 0.15 | 3.13 | 2.15E-17 | 1245 | 367.88 | 877.12 |
| scaffold398405.1_Spipo2G0017100 | 0.38 | 0.07 | 5.59 | 6.52E-08 | 387 | 110.57 | 276.43 |
| scaffold401286.1_Spipo2G0011700 | 0.18 | 0.04 | 4.03 | 4.50E-09 | 1056 | 342.77 | 713.23 |
| scaffold403838.1_Spipo17G0034000 | 0.25 | 0.01 | 16.83 | 0.0003348 | 174 | 51.19 | 122.81 |
| scaffold409993.1_Spipo21G0035400 | 0.24 | 0.07 | 3.44 | 0.0079078 | 222 | 62.23 | 159.77 |
| scaffold414224.1_Spipo21G0029600 | 0.21 | 0.06 | 3.40 | 0.0049623 | 189 | 100.74 | 88.26 |
| scaffold416266.1_Spipo15G0025200 | 0.56 | 0.31 | 1.80 | 0.0003493 | 480 | 134.99 | 345.01 |
| scaffold416732.1_Spipo2G0086200 | 0.24 | 0.06 | 4.15 | 9.41E-09 | 801 | 234.31 | 566.69 |
| scaffold419617.1_Spipo0G0125200 | 0.39 | 0.16 | 2.43 | 0.0030971 | 201 | 85.99 | 115.01 |
| scaffold419617.1_Spipo6G0056200 | 0.55 | 0.32 | 1.72 | 3.07E-05 | 678 | 241.42 | 436.58 |
| scaffold421370.1_Spipo2G0108000 | 0.59 | 0.19 | 3.02 | 6.51E-32 | 1770 | 589.33 | 1180.67 |
| scaffold429147.1_Spipo4G0012600 | 0.24 | 0.08 | 2.87 | 0.0006228 | 450 | 114.67 | 335.33 |
| scaffold431662.1_Spipo0G0089400 | 0.39 | 0.06 | 7.04 | 1.38E-23 | 846 | 361.04 | 484.96 |
| scaffold440051.2_Spipo7G0046700 | 0.44 | 0.19 | 2.34 | 4.32E-05 | 402 | 123.56 | 278.45 |
| scaffold441434.1_Spipo20G0023700 | 0.40 | 0.24 | 1.70 | 2.37E-06 | 1182 | 392.13 | 789.87 |
| scaffold442512.2_Spipo17G0007800 | 0.50 | 0.21 | 2.43 | 2.73E-13 | 1164 | 322.55 | 841.45 |
| scaffold444157.2_Spipo16G0016700 | 0.35 | 0.14 | 2.49 | 1.68E-05 | 501 | 157.70 | 343.30 |
| scaffold446685.1_Spipo16G0034700 | 0.87 | 0.19 | 4.52 | 1.07E-08 | 267 | 73.88 | 193.12 |
| scaffold446685.1_Spipo1G0036700 | 0.76 | 0.17 | 4.61 | 6.96E-06 | 210 | 44.71 | 165.29 |
| scaffold449603.1_Spipo5G0021300 | 0.43 | 0.12 | 3.68 | 5.22E-11 | 687 | 208.79 | 478.21 |
| scaffold452064.1_Spipo0G0072300 | 0.29 | 0.08 | 3.82 | 4.11E-06 | 429 | 144.37 | 284.63 |
| scaffold455866.1_Spipo12G0036700 | 0.45 | 0.20 | 2.28 | 2.14E-12 | 1224 | 410.61 | 813.39 |
| scaffold456637.1_Spipo5G0034600 | 0.34 | 0.07 | 4.86 | 1.44E-14 | 891 | 298.35 | 592.65 |
| scaffold456912.1_Spipo22G0006800 | 0.36 | 0.19 | 1.91 | 0.0021501 | 387 | 129.15 | 257.85 |
| scaffold457974.1_Spipo14G0049300 | 0.24 | 0.08 | 2.87 | 3.17E-13 | 1755 | 538.54 | 1216.46 |
| scaffold459618.1_Spipo16G0033200 | 0.54 | 0.27 | 2.01 | 2.58E-14 | 1638 | 534.38 | 1103.62 |
| scaffold461444.1_Spipo8G0039700 | 0.22 | 0.06 | 3.92 | 3.73E-10 | 1119 | 298.42 | 820.58 |
| scaffold462087.1_Spipo8G0072000 | 0.28 | 0.08 | 3.68 | 2.15E-15 | 1368 | 428.58 | 939.43 |
| scaffold463399.1_Spipo4G0030300 | 0.33 | 0.15 | 2.15 | 2.11E-05 | 753 | 223.51 | 529.49 |
| scaffold463987.1_Spipo0G0099100 | 0.20 | 0.07 | 2.95 | 0.0085937 | 318 | 80.24 | 237.76 |
| scaffold46845.1_Spipo1G0085300 | 0.29 | 0.08 | 3.54 | 1.24E-17 | 1443 | 513.89 | 929.11 |
| scaffold469586.1_Spipo23G0038900 | 0.27 | 0.10 | 2.79 | 1.42E-06 | 633 | 239.98 | 393.02 |
| scaffold480113.1_Spipo21G0015900 | 0.23 | 0.09 | 2.61 | 0.0023508 | 333 | 121.09 | 211.91 |
| scaffold481615.1_Spipo9G0066900 | 0.30 | 0.13 | 2.27 | 0.0029346 | 381 | 118.85 | 262.15 |
| scaffold487300.1_Spipo12G0028800 | 0.23 | 0.01 | 18.69 | 1.45E-05 | 225 | 68.68 | 156.32 |
| scaffold490075.1_Spipo4G0036400 | 0.28 | 0.10 | 2.93 | 1.41E-09 | 981 | 320.76 | 660.24 |
| scaffold491888.1_Spipo21G0020400 | 0.50 | 0.32 | 1.57 | 0.003039 | 492 | 173.42 | 318.58 |
| scaffold507620.1_Spipo5G0034600 | 0.34 | 0.08 | 4.16 | 1.63E-10 | 813 | 233.56 | 579.44 |
| scaffold507719.1_Spipo11G0042400 | 0.51 | 0.13 | 4.01 | 5.87E-08 | 318 | 128.28 | 189.72 |
| scaffold50876.2_Spipo6G0056200 | 0.53 | 0.18 | 2.98 | 0.0005599 | 204 | 62.21 | 141.79 |
| scaffold510526.1_Spipo2G0019200 | 0.41 | 0.23 | 1.77 | 0.0026966 | 498 | 128.55 | 369.45 |
| scaffold513290.1_Spipo9G0056500 | 0.43 | 0.16 | 2.72 | 3.51E-14 | 1323 | 358.24 | 964.76 |
| scaffold513608.1_Spipo19G0013700 | 0.22 | 0.03 | 7.85 | 3.12E-13 | 960 | 282.27 | 677.73 |
| scaffold51484.1_Spipo1G0092400 | 0.46 | 0.25 | 1.83 | 7.27E-07 | 945 | 322.60 | 622.40 |
| scaffold518966.1_Spipo9G0062300 | 0.22 | 0.09 | 2.42 | 0.0002332 | 654 | 189.09 | 464.91 |
| scaffold526980.1_Spipo15G0010100 | 0.43 | 0.23 | 1.88 | 0.0003208 | 477 | 179.45 | 297.55 |
| scaffold53059.1_Spipo22G0016800 | 0.32 | 0.06 | 5.30 | 2.87E-12 | 840 | 223.72 | 616.28 |
| scaffold533916.1_Spipo8G0010700 | 1.34 | 0.43 | 3.10 | 3.82E-06 | 186 | 70.30 | 115.70 |
| scaffold53410.1_Spipo25G0006500 | 0.40 | 0.05 | 7.49 | 9.03E-07 | 288 | 81.87 | 206.13 |
| scaffold53924.1_Spipo11G0033300 | 0.19 | 0.07 | 2.66 | 0.0005018 | 615 | 171.29 | 443.71 |
| scaffold539340.1_Spipo19G0026700 | 0.35 | 0.10 | 3.56 | 1.46E-07 | 654 | 155.58 | 498.42 |
| scaffold542888.1_Spipo20G0006500 | 0.25 | 0.07 | 3.38 | 2.64E-09 | 1038 | 265.75 | 772.25 |
| scaffold545752.1_Spipo13G0051300 | 0.53 | 0.27 | 1.98 | 1.67E-17 | 2091 | 638.26 | 1452.74 |
| scaffold550580.1_Spipo4G0016800 | 0.32 | 0.08 | 3.80 | 6.25E-08 | 603 | 162.60 | 440.40 |
| scaffold554352.1_Spipo6G0045500 | 0.18 | 0.04 | 5.14 | 2.66E-06 | 669 | 172.87 | 496.13 |
| scaffold560090.1_Spipo0G0009900 | 0.29 | 0.14 | 2.14 | 0.0012368 | 435 | 185.73 | 249.27 |
| scaffold56225.1_Spipo4G0078200 | 0.21 | 0.04 | 5.20 | 3.88E-13 | 1035 | 387.83 | 647.17 |
| scaffold563756.1_Spipo12G0036700 | 0.31 | 0.13 | 2.28 | 0.0078401 | 279 | 80.47 | 198.53 |
| scaffold573328.1_Spipo7G0053500 | 0.22 | 0.07 | 3.00 | 0.0002562 | 519 | 134.38 | 384.62 |
| scaffold576388.1_Spipo2G0070100 | 0.25 | 0.07 | 3.64 | 7.10E-17 | 1479 | 561.69 | 917.31 |
| scaffold576849.1_Spipo16G0004300 | 0.41 | 0.09 | 4.59 | 8.11E-05 | 153 | 69.15 | 83.85 |
| scaffold577538.1_Spipo14G0001800 | 0.45 | 0.09 | 5.02 | 1.32E-13 | 612 | 187.18 | 424.82 |
| scaffold580409.1_Spipo4G0012100 | 0.25 | 0.05 | 4.83 | 1.20E-05 | 426 | 123.96 | 302.04 |
| scaffold587783.1_Spipo0G0042200 | 0.36 | 0.15 | 2.46 | 6.51E-08 | 927 | 239.08 | 687.92 |
| scaffold589215.1_Spipo3G0028300 | 0.40 | 0.08 | 5.29 | 6.24E-05 | 174 | 63.13 | 110.87 |
| scaffold590339.2_Spipo21G0017500 | 0.70 | 0.51 | 1.38 | 0.0032546 | 765 | 227.39 | 537.62 |
| scaffold590339.2_Spipo21G0017500 | 0.85 | 0.46 | 1.82 | 1.73E-10 | 843 | 325.31 | 517.69 |
| scaffold601390.1_Spipo15G0037800 | 0.20 | 0.08 | 2.46 | 0.0035246 | 417 | 140.28 | 276.72 |
| scaffold601390.1_Spipo5G0065600 | 0.48 | 0.07 | 7.15 | 9.71E-14 | 417 | 158.24 | 258.76 |
| scaffold603754.1_Spipo6G0025400 | 0.29 | 0.11 | 2.60 | 0.0084356 | 312 | 70.10 | 241.90 |
| scaffold618883.1_Spipo2G0040400 | 0.31 | 0.19 | 1.65 | 9.40E-05 | 1296 | 404.30 | 891.70 |
| scaffold626802.1_Spipo9G0019300 | 0.30 | 0.08 | 3.50 | 0.0056319 | 177 | 55.52 | 121.48 |
| scaffold63459.1_Spipo2G0057000 | 0.35 | 0.06 | 5.52 | 1.96E-10 | 483 | 157.69 | 325.31 |
| scaffold637018.1_Spipo0G0126300 | 0.45 | 0.24 | 1.89 | 0.0047301 | 273 | 107.70 | 165.30 |
| scaffold637039.2_Spipo12G0030800 | 0.31 | 0.14 | 2.30 | 0.0039284 | 354 | 88.11 | 265.89 |
| scaffold638915.1_Spipo3G0089500 | 0.35 | 0.03 | 12.09 | 4.77E-30 | 1074 | 393.92 | 680.08 |
| scaffold638915.1_Spipo3G0089600 | 0.36 | 0.03 | 13.86 | 1.28E-31 | 1083 | 396.69 | 686.32 |
| scaffold642497.1_Spipo11G0042500 | 0.43 | 0.16 | 2.63 | 6.80E-11 | 834 | 300.20 | 533.80 |
| scaffold642497.1_Spipo11G0042500 | 0.88 | 0.30 | 2.91 | 0.0017262 | 141 | 45.22 | 95.78 |
| scaffold643573.2_Spipo0G0056100 | 0.47 | 0.29 | 1.62 | 3.11E-05 | 1041 | 311.47 | 729.53 |
| scaffold64567.1_Spipo5G0001800 | 0.30 | 0.14 | 2.11 | 0.0004907 | 537 | 194.28 | 342.72 |
| scaffold647423.1_Spipo9G0002400 | 0.44 | 0.25 | 1.76 | 0.0023879 | 411 | 151.97 | 259.03 |
| scaffold651603.1_Spipo28G0013700 | 0.29 | 0.07 | 4.25 | 4.64E-13 | 1089 | 293.00 | 796.00 |
| scaffold655399.2_Spipo14G0023000 | 0.36 | 0.13 | 2.69 | 0.0008997 | 270 | 92.11 | 177.89 |
| scaffold657781.1_Spipo5G0009400 | 0.15 | 0.00 | 50.00 | 0.0063266 | 138 | 44.29 | 93.71 |
| scaffold663509.1_Spipo13G0051500 | 0.32 | 0.07 | 4.34 | 1.10E-21 | 1251 | 529.30 | 721.70 |
| scaffold666038.1_Spipo26G0001900 | 0.42 | 0.23 | 1.78 | 0.0003113 | 531 | 193.95 | 337.05 |
| scaffold671042.3_Spipo0G0014900 | 0.87 | 0.57 | 1.53 | 0.0041172 | 408 | 104.98 | 303.02 |
| scaffold671923.1_Spipo9G0033400 | 0.55 | 0.01 | 40.57 | 0.0019501 | 57 | 30.56 | 26.44 |
| scaffold683507.1_Spipo13G0005500 | 0.31 | 0.12 | 2.60 | 3.76E-05 | 504 | 163.05 | 340.95 |
| scaffold686008.1_Spipo13G0039600 | 0.28 | 0.09 | 3.27 | 4.99E-08 | 792 | 212.13 | 579.87 |
| scaffold686423.1_Spipo28G0017100 | 0.59 | 0.08 | 7.11 | 0.0001133 | 126 | 38.86 | 87.14 |
| scaffold686766.1_Spipo0G0052100 | 0.41 | 0.17 | 2.38 | 0.0026942 | 186 | 87.08 | 98.92 |
| scaffold689856.1_Spipo0G0031000 | 0.41 | 0.11 | 3.79 | 1.56E-14 | 759 | 296.95 | 462.05 |
| scaffold694573.1_Spipo4G0005100 | 0.30 | 0.08 | 3.89 | 3.20E-08 | 681 | 179.30 | 501.70 |
| scaffold699753.1_Spipo3G0077900 | 0.24 | 0.06 | 4.05 | 4.03E-10 | 1089 | 267.96 | 821.05 |
| scaffold701390.1_Spipo26G0003700 | 0.34 | 0.15 | 2.36 | 5.44E-08 | 1044 | 286.41 | 757.59 |
| scaffold704093.1_Spipo16G0007500 | 0.44 | 0.23 | 1.91 | 0.0005819 | 450 | 140.62 | 309.38 |
| scaffold70526.1_Spipo0G0034300 | 0.33 | 0.13 | 2.56 | 0.0010442 | 288 | 119.11 | 168.89 |
| scaffold706244.1_Spipo7G0053500 | 0.21 | 0.07 | 3.05 | 0.0009008 | 480 | 117.32 | 362.68 |
| scaffold711726.1_Spipo23G0036900 | 0.28 | 0.15 | 1.84 | 0.0096883 | 603 | 136.84 | 466.16 |
| scaffold71402.1_Spipo3G0048100 | 0.32 | 0.11 | 2.86 | 1.97E-09 | 768 | 307.18 | 460.82 |
| scaffold715312.1_Spipo12G0029600 | 0.18 | 0.00 | 50.00 | 0.0033294 | 141 | 52.21 | 88.79 |
| scaffold71636.1_Spipo7G0062500 | 0.37 | 0.03 | 14.43 | 3.18E-17 | 537 | 209.42 | 327.59 |
| scaffold721207.1_Spipo12G0014200 | 0.44 | 0.05 | 8.98 | 3.61E-25 | 792 | 316.76 | 475.24 |
| scaffold7264.1_Spipo20G0012700 | 0.66 | 0.22 | 3.02 | 3.74E-05 | 168 | 67.72 | 100.28 |
| scaffold729129.1_Spipo0G0041100 | 0.29 | 0.06 | 4.90 | 5.00E-05 | 273 | 97.04 | 175.96 |
| scaffold731310.1_Spipo12G0053900 | 0.22 | 0.12 | 1.89 | 6.87E-06 | 1455 | 496.41 | 958.60 |
| scaffold731389.1_Spipo6G0016000 | 0.40 | 0.12 | 3.26 | 4.61E-13 | 963 | 286.54 | 676.46 |
| scaffold732218.2_Spipo8G0051800 | 0.17 | 0.08 | 2.24 | 0.0092562 | 522 | 139.73 | 382.27 |
| scaffold738620.1_Spipo4G0073200 | 0.36 | 0.12 | 2.92 | 1.23E-22 | 1905 | 712.46 | 1192.54 |
| scaffold746411.1_Spipo11G0061300 | 0.36 | 0.11 | 3.39 | 1.98E-06 | 384 | 149.35 | 234.66 |
| scaffold747210.1_Spipo4G0046200 | 0.71 | 0.35 | 2.03 | 9.27E-07 | 468 | 154.30 | 313.70 |
| scaffold753063.1_Spipo3G0060300 | 0.23 | 0.10 | 2.22 | 0.0027117 | 417 | 159.46 | 257.54 |
| scaffold757470.1_Spipo8G0064600 | 0.43 | 0.10 | 4.23 | 1.32E-23 | 1602 | 396.23 | 1205.77 |
| scaffold759829.1_Spipo17G0029800 | 0.46 | 0.10 | 4.73 | 2.65E-13 | 513 | 213.24 | 299.76 |
| scaffold760683.1_Spipo19G0005100 | 0.58 | 0.20 | 2.96 | 1.38E-09 | 531 | 163.28 | 367.73 |
| scaffold764755.1_Spipo2G0055600 | 0.43 | 0.04 | 11.26 | 6.43E-15 | 480 | 159.69 | 320.32 |
| scaffold768808.2_Spipo4G0114700 | 0.18 | 0.01 | 29.26 | 3.51E-08 | 339 | 183.23 | 155.77 |
| scaffold771889.1_Spipo16G0042000 | 0.25 | 0.08 | 3.26 | 3.38E-11 | 1065 | 392.20 | 672.80 |
| scaffold771889.1_Spipo1G0018200 | 0.21 | 0.07 | 3.07 | 3.66E-09 | 1095 | 404.94 | 690.07 |
| scaffold774392.1_Spipo0G0146600 | 0.23 | 0.04 | 5.49 | 5.91E-07 | 462 | 155.13 | 306.87 |
| scaffold777816.1_Spipo6G0062600 | 0.56 | 0.14 | 4.08 | 2.73E-14 | 525 | 231.43 | 293.57 |
| scaffold779770.1_Spipo9G0066900 | 0.30 | 0.13 | 2.27 | 0.0029346 | 381 | 118.85 | 262.15 |
| scaffold781854.1_Spipo12G0057500 | 0.22 | 0.07 | 3.27 | 1.64E-07 | 1011 | 248.18 | 762.82 |
| scaffold78356.1_Spipo22G0012000 | 0.30 | 0.14 | 2.17 | 1.88E-09 | 1515 | 471.24 | 1043.76 |
| scaffold785747.1_Spipo11G0042100 | 0.46 | 0.16 | 2.93 | 1.57E-20 | 1221 | 512.88 | 708.12 |
| scaffold788052.1_Spipo0G0039500 | 0.21 | 0.03 | 7.59 | 3.80E-08 | 597 | 161.29 | 435.71 |
| scaffold788828.1_Spipo23G0017300 | 0.34 | 0.20 | 1.73 | 1.61E-06 | 1800 | 428.93 | 1371.07 |
| scaffold789764.1_Spipo10G0017900 | 0.69 | 0.23 | 3.00 | 4.69E-15 | 846 | 226.77 | 619.23 |
| scaffold789764.1_Spipo12G0031500 | 0.35 | 0.10 | 3.41 | 3.23E-22 | 2016 | 522.88 | 1493.12 |
| scaffold790805.2_Spipo3G0098400 | 0.51 | 0.27 | 1.86 | 0.0021765 | 354 | 103.98 | 250.02 |
| scaffold792602.1_Spipo5G0024800 | 0.49 | 0.31 | 1.60 | 1.54E-10 | 2931 | 684.66 | 2246.34 |
| scaffold793834.3_Spipo1G0045500 | 0.24 | 0.12 | 1.98 | 0.0097671 | 393 | 135.94 | 257.06 |
| scaffold796194.1_Spipo4G0115200 | 0.29 | 0.16 | 1.84 | 0.0006963 | 738 | 227.33 | 510.67 |
| scaffold797300.1_Spipo4G0114700 | 0.16 | 0.04 | 4.24 | 0.0007068 | 336 | 133.72 | 202.28 |
| scaffold797414.1_Spipo19G0026200 | 0.29 | 0.08 | 3.68 | 1.48E-06 | 444 | 163.62 | 280.38 |
| scaffold799326.1_Spipo0G0083000 | 0.61 | 0.29 | 2.08 | 3.44E-09 | 726 | 248.70 | 477.30 |
| scaffold799622.1_Spipo12G0065900 | 0.19 | 0.08 | 2.23 | 0.0047184 | 636 | 155.76 | 480.24 |
| scaffold800567.2_Spipo12G0022900 | 0.33 | 0.06 | 5.30 | 0.0002 | 231 | 57.61 | 173.39 |
| scaffold80964.1_Spipo14G0033700 | 0.28 | 0.11 | 2.54 | 9.04E-07 | 891 | 271.81 | 619.19 |
| scaffold80964.4_Spipo14G0033700 | 0.28 | 0.11 | 2.54 | 9.04E-07 | 891 | 271.81 | 619.19 |
| scaffold810324.1_Spipo3G0087900 | 0.25 | 0.08 | 3.21 | 0.0067126 | 255 | 68.01 | 186.99 |
| scaffold816655.1_Spipo21G0020400 | 0.49 | 0.32 | 1.53 | 0.0040961 | 492 | 173.70 | 318.30 |
| scaffold826810.1_Spipo15G0031400 | 0.25 | 0.10 | 2.60 | 1.21E-12 | 1722 | 605.29 | 1116.71 |
| scaffold826810.1_Spipo22G0043000 | 0.57 | 0.32 | 1.78 | 1.71E-10 | 1377 | 477.32 | 899.68 |
| scaffold829486.1_Spipo17G0021100 | 0.21 | 0.03 | 6.42 | 0.0010755 | 234 | 72.51 | 161.49 |
| scaffold829759.1_Spipo22G0023700 | 0.29 | 0.09 | 3.18 | 0.0005127 | 369 | 98.43 | 270.57 |
| scaffold832371.1_Spipo2G0040100 | 0.33 | 0.14 | 2.42 | 4.44E-06 | 552 | 227.75 | 324.26 |
| scaffold83812.1_Spipo2G0029200 | 0.38 | 0.10 | 3.78 | 0.0014877 | 234 | 58.92 | 175.08 |
| scaffold841212.1_Spipo4G0064200 | 0.23 | 0.08 | 2.75 | 0.0040739 | 399 | 105.11 | 293.89 |
| scaffold844015.1_Spipo2G0068200 | 0.61 | 0.37 | 1.68 | 1.17E-06 | 873 | 317.77 | 555.23 |
| scaffold845830.1_Spipo2G0049700 | 0.31 | 0.10 | 3.15 | 1.99E-09 | 948 | 257.83 | 690.17 |
| scaffold848204.1_Spipo0G0056200 | 0.22 | 0.04 | 5.95 | 5.22E-12 | 933 | 297.61 | 635.39 |
| scaffold85109.1_Spipo5G0024800 | 0.44 | 0.28 | 1.55 | 3.72E-10 | 3162 | 844.16 | 2317.85 |
| scaffold85109.1_Spipo5G0024800 | 0.52 | 0.05 | 10.13 | 0.0064668 | 54 | 18.47 | 35.53 |
| scaffold852794.1_Spipo23G0033600 | 0.29 | 0.15 | 1.89 | 1.59E-06 | 1260 | 438.68 | 821.32 |
| scaffold854867.1_Spipo12G0047200 | 0.25 | 0.04 | 6.60 | 0.0015771 | 177 | 56.93 | 120.07 |
| scaffold856897.1_Spipo3G0037000 | 0.25 | 0.02 | 10.35 | 0.0003818 | 255 | 78.47 | 176.53 |
| scaffold857186.3_Spipo3G0064300 | 0.57 | 0.24 | 2.42 | 1.41E-10 | 771 | 246.94 | 524.06 |
| scaffold858726.1_Spipo4G0011400 | 0.22 | 0.05 | 4.49 | 1.54E-08 | 927 | 229.70 | 697.30 |
| scaffold865571.1_Spipo0G0043500 | 1.03 | 0.03 | 34.65 | 0.0035762 | 102 | 18.57 | 83.43 |
| scaffold866160.1_Spipo9G0066900 | 0.29 | 0.10 | 3.02 | 3.26E-05 | 381 | 151.92 | 229.08 |
| scaffold86792.1_Spipo15G0001600 | 0.46 | 0.24 | 1.90 | 0.0005051 | 375 | 140.06 | 234.95 |
| scaffold87401.1_Spipo9G0051600 | 0.42 | 0.23 | 1.81 | 5.16E-08 | 1431 | 400.18 | 1030.82 |
| scaffold874313.1_Spipo12G0044300 | 0.30 | 0.17 | 1.80 | 0.0009239 | 714 | 235.71 | 478.29 |
| scaffold874313.1_Spipo28G0011400 | 0.46 | 0.34 | 1.37 | 0.0002301 | 1839 | 482.37 | 1356.63 |
| scaffold876348.1_Spipo2G0064800 | 0.54 | 0.15 | 3.68 | 6.16E-16 | 663 | 261.87 | 401.13 |
| scaffold877141.2_Spipo13G0022100 | 0.52 | 0.26 | 1.99 | 0.00131 | 264 | 102.68 | 161.32 |
| scaffold877978.1_Spipo0G0174700 | 0.46 | 0.07 | 6.20 | 6.83E-15 | 516 | 200.08 | 315.92 |
| scaffold879964.2_Spipo21G0018900 | 0.25 | 0.07 | 3.52 | 1.89E-07 | 657 | 210.84 | 446.16 |
| scaffold881862.1_Spipo28G0019000 | 0.18 | 0.02 | 10.52 | 7.68E-05 | 234 | 100.76 | 133.25 |
| scaffold881862.1_Spipo5G0005800 | 0.23 | 0.02 | 13.71 | 2.15E-06 | 231 | 111.99 | 119.01 |
| scaffold884795.1_Spipo18G0015600 | 0.65 | 0.29 | 2.28 | 0.0002222 | 225 | 84.79 | 140.21 |
| scaffold886326.1_Spipo17G0029600 | 0.50 | 0.12 | 4.22 | 1.44E-24 | 1176 | 363.68 | 812.32 |
| scaffold897242.1_Spipo6G0040400 | 0.30 | 0.11 | 2.88 | 4.37E-08 | 891 | 244.23 | 646.78 |
| scaffold913563.1_Spipo0G0049100 | 0.28 | 0.12 | 2.25 | 1.22E-08 | 1305 | 434.69 | 870.31 |
| scaffold913563.1_Spipo0G0049100 | 0.94 | 0.56 | 1.68 | 4.59E-06 | 783 | 229.97 | 553.03 |
| scaffold913563.1_Spipo0G0049100 | 1.81 | 0.57 | 3.17 | 5.85E-12 | 249 | 162.82 | 86.18 |
| scaffold913563.1_Spipo0G0049100 | 2.82 | 0.50 | 5.58 | 3.06E-19 | 210 | 165.00 | 45.00 |
| scaffold913563.1_Spipo0G0049100 | 1.78 | 0.30 | 5.86 | 2.11E-16 | 222 | 127.36 | 94.64 |
| scaffold913563.1_Spipo0G0049100 | 3.29 | 0.45 | 7.38 | 0 | 261 | 210.15 | 50.85 |
| scaffold913563.1_Spipo0G0049100 | 2.48 | 0.46 | 5.41 | 2.92E-21 | 258 | 188.79 | 69.21 |
| scaffold915865.1_Spipo15G0042500 | 0.38 | 0.15 | 2.53 | 9.73E-07 | 558 | 191.35 | 366.65 |
| scaffold916357.1_Spipo6G0067200 | 0.26 | 0.06 | 4.34 | 8.78E-10 | 864 | 235.59 | 628.41 |
| scaffold924115.1_Spipo9G0044400 | 0.33 | 0.20 | 1.62 | 0.0018341 | 879 | 227.18 | 651.82 |
| scaffold927372.3_Spipo1G0063400 | 0.59 | 0.23 | 2.59 | 0.0012004 | 177 | 53.48 | 123.52 |
| scaffold932489.1_Spipo3G0052800 | 0.17 | 0.04 | 4.44 | 0.0045631 | 258 | 75.58 | 182.42 |
| scaffold943052.1_Spipo26G0022200 | 0.56 | 0.19 | 2.88 | 0.0003753 | 198 | 64.71 | 133.29 |
| scaffold944490.1_Spipo1G0124500 | 0.36 | 0.20 | 1.80 | 0.0018514 | 471 | 163.64 | 307.36 |
| scaffold945969.1_Spipo14G0052800 | 0.27 | 0.08 | 3.23 | 3.97E-05 | 480 | 129.27 | 350.73 |
| scaffold947878.1_Spipo7G0015700 | 0.35 | 0.15 | 2.36 | 1.04E-08 | 930 | 327.84 | 602.16 |
| scaffold948265.1_Spipo28G0008700 | 0.61 | 0.22 | 2.77 | 2.82E-14 | 792 | 273.78 | 518.22 |
| scaffold953531.1_Spipo22G0022500 | 0.54 | 0.20 | 2.65 | 8.00E-05 | 360 | 94.52 | 265.48 |
| scaffold959560.1_Spipo29G0001100 | 0.25 | 0.02 | 12.44 | 9.43E-05 | 273 | 71.58 | 201.42 |
| scaffold96230.1_Spipo6G0075500 | 0.56 | 0.38 | 1.48 | 6.10E-06 | 1314 | 476.88 | 837.12 |
| scaffold965180.2_Spipo9G0042100 | 0.40 | 0.19 | 2.15 | 0.0003356 | 405 | 127.39 | 277.62 |
| scaffold966986.1_Spipo30G0016000 | 0.30 | 0.12 | 2.58 | 9.60E-05 | 600 | 141.39 | 458.61 |
| scaffold976741.1_Spipo9G0022500 | 0.42 | 0.16 | 2.65 | 6.43E-23 | 2091 | 663.29 | 1427.71 |
| scaffold976753.1_Spipo15G0030700 | 0.30 | 0.08 | 3.69 | 1.09E-15 | 1239 | 416.72 | 822.28 |
| scaffold978133.1_Spipo6G0061400 | 0.24 | 0.05 | 4.57 | 0.0009473 | 297 | 67.13 | 229.87 |
| scaffold982642.1_Spipo12G0025800 | 0.25 | 0.15 | 1.71 | 0.0031459 | 798 | 227.20 | 570.80 |
| scaffold990705.1_Spipo31G0004700 | 0.30 | 0.09 | 3.18 | 2.47E-18 | 1626 | 558.23 | 1067.77 |
| scaffold993192.1_Spipo6G0053800 | 0.34 | 0.18 | 1.89 | 1.49E-05 | 1038 | 291.52 | 746.48 |

**Table S12.** The positively selected genes of *A. konjac* against *Z. marina*.

| Sequence | Ka | Ks | Ka/Ks | P-Value | Length | S-Sites | N-Sites |
| --- | --- | --- | --- | --- | --- | --- | --- |
| C424683633.1_Zosma401g00050 | 0.82 | 0.20 | 4.20 | 4.04E-05 | 135 | 45.08 | 89.92 |
| C425826784.1_Zosma154g00170 | 0.54 | 0.12 | 4.60 | 0.0001706 | 156 | 53.81 | 102.20 |
| C426475223.1_Zosma196g00110 | 0.58 | 0.17 | 3.34 | 1.05E-08 | 345 | 124.47 | 220.53 |
| C426677459.1_Zosma122g00290 | 0.45 | 0.12 | 3.76 | 1.56E-05 | 225 | 78.38 | 146.62 |
| C426894041.1_Zosma109g00270 | 0.60 | 0.36 | 1.67 | 0.0002653 | 498 | 159.37 | 338.64 |
| C426955743.1_Zosma49g00690 | 0.52 | 0.10 | 4.96 | 4.50E-05 | 153 | 65.85 | 87.15 |
| C427104929.1_Zosma72g00750 | 0.34 | 0.03 | 10.34 | 8.68E-10 | 453 | 114.53 | 338.47 |
| C427147617.1_Zosma27g01330 | 0.56 | 0.12 | 4.62 | 0.0055324 | 96 | 22.02 | 73.98 |
| C427316452.1_Zosma26g00330 | 0.64 | 0.20 | 3.18 | 4.99E-32 | 1551 | 542.24 | 1008.77 |
| C427332934.1_Zosma43g00020 | 0.40 | 0.25 | 1.56 | 0.0019032 | 777 | 247.92 | 529.08 |
| C427332934.1_Zosma89g00590 | 0.53 | 0.27 | 2.00 | 4.22E-08 | 966 | 274.55 | 691.46 |
| C427346272.1_Zosma29g01140 | 0.44 | 0.15 | 2.89 | 4.34E-22 | 1704 | 529.42 | 1174.58 |
| C427401824.1_Zosma2g03440 | 0.55 | 0.17 | 3.19 | 1.27E-30 | 2208 | 518.31 | 1689.69 |
| C427402514.1_Zosma182g00280 | 0.43 | 0.12 | 3.59 | 1.18E-15 | 981 | 290.47 | 690.53 |
| C427404640.1_Zosma93g00850 | 0.55 | 0.18 | 3.12 | 1.60E-17 | 1056 | 305.27 | 750.73 |
| C427407770.1_Zosma11g00030 | 0.36 | 0.18 | 1.99 | 0.0019851 | 501 | 121.82 | 379.18 |
| scaffold1002908.2_Zosma248g00480 | 0.38 | 0.15 | 2.50 | 0.0006393 | 306 | 88.91 | 217.09 |
| scaffold1027169.1_Zosma120g00260 | 0.41 | 0.07 | 5.93 | 1.49E-11 | 522 | 155.35 | 366.65 |
| scaffold1033849.1_Zosma41g01050 | 0.51 | 0.11 | 4.81 | 3.11E-08 | 282 | 109.31 | 172.69 |
| scaffold1055544.1_Zosma77g00150 | 0.40 | 0.03 | 13.04 | 1.01E-14 | 609 | 154.31 | 454.70 |
| scaffold1057455.1_Zosma29g00640 | 0.28 | 0.05 | 5.34 | 0.0009552 | 222 | 55.77 | 166.23 |
| scaffold1057463.1_Zosma37g01450 | 0.62 | 0.23 | 2.73 | 6.72E-25 | 1575 | 451.89 | 1123.11 |
| scaffold1061366.1_Zosma31g00270 | 0.39 | 0.20 | 1.92 | 1.69E-07 | 1185 | 362.31 | 822.69 |
| scaffold1076817.1_Zosma159g00230 | 0.40 | 0.08 | 4.98 | 1.97E-43 | 2193 | 758.45 | 1434.55 |
| scaffold1087256.1_Zosma28g00590 | 0.32 | 0.06 | 5.36 | 1.28E-05 | 315 | 85.75 | 229.25 |
| scaffold1101637.2_Zosma150g00010 | 0.35 | 0.05 | 7.31 | 3.66E-11 | 486 | 143.99 | 342.01 |
| scaffold1104947.1_ZosmaM1g00630 | 0.55 | 0.32 | 1.71 | 0.0001703 | 669 | 170.77 | 498.23 |
| scaffold1128397.1_Zosma81g00570 | 0.42 | 0.27 | 1.57 | 0.0071508 | 585 | 166.52 | 418.48 |
| scaffold1136263.1_Zosma154g00170 | 0.71 | 0.07 | 10.51 | 2.80E-07 | 159 | 53.26 | 105.74 |
| scaffold1163397.2_Zosma47g00200 | 0.47 | 0.14 | 3.40 | 7.12E-12 | 708 | 200.54 | 507.46 |
| scaffold1169782.1_Zosma179g00170 | 0.53 | 0.18 | 2.91 | 4.21E-11 | 855 | 192.13 | 662.87 |
| scaffold1177869.1_Zosma54g01110 | 0.70 | 0.27 | 2.61 | 1.07E-12 | 651 | 220.75 | 430.25 |
| scaffold1186773.1_Zosma41g00780 | 0.37 | 0.10 | 3.75 | 5.82E-08 | 564 | 145.03 | 418.97 |
| scaffold1188029.1_Zosma164g00190 | 0.42 | 0.09 | 4.59 | 8.10E-25 | 1266 | 481.43 | 784.57 |
| scaffold1205512.1_Zosma279g00040 | 0.42 | 0.12 | 3.35 | 0.0003439 | 237 | 73.01 | 163.99 |
| scaffold1209880.1_Zosma69g00630 | 0.48 | 0.09 | 5.57 | 3.00E-05 | 189 | 56.38 | 132.62 |
| scaffold1211590.1_Zosma79g00550 | 0.53 | 0.16 | 3.33 | 1.54E-36 | 2709 | 608.65 | 2100.35 |
| scaffold121248.1_Zosma64g00930 | 0.86 | 0.14 | 6.17 | 6.68E-05 | 123 | 35.33 | 87.67 |
| scaffold1288005.1_Zosma186g00050 | 0.36 | 0.14 | 2.63 | 2.49E-23 | 2604 | 750.07 | 1853.93 |
| scaffold1288352.1_Zosma75g00580 | 0.43 | 0.12 | 3.58 | 1.18E-56 | 3579 | 1182.68 | 2396.32 |
| scaffold1288880.1_Zosma214g00120 | 0.40 | 0.10 | 4.02 | 4.49E-17 | 1221 | 354.03 | 866.97 |
| scaffold1303352.1_Zosma8g01150 | 0.30 | 0.17 | 1.78 | 0.0026295 | 639 | 193.44 | 445.56 |
| scaffold1314171.1_Zosma251g00300 | 0.48 | 0.17 | 2.76 | 2.23E-12 | 942 | 290.19 | 651.81 |
| scaffold1314948.1_Zosma55g00370 | 0.32 | 0.15 | 2.16 | 0.0002229 | 582 | 151.43 | 430.57 |
| scaffold1322752.1_Zosma53g00400 | 0.30 | 0.10 | 2.96 | 0.0051675 | 234 | 64.60 | 169.40 |
| scaffold1325922.1_Zosma271g00240 | 0.88 | 0.32 | 2.75 | 2.18E-33 | 1377 | 458.51 | 918.49 |
| scaffold1339604.1_Zosma11g00220 | 0.53 | 0.36 | 1.49 | 4.92E-07 | 2133 | 544.74 | 1588.26 |
| scaffold1356350.1_Zosma40g00360 | 0.75 | 0.23 | 3.27 | 6.67E-17 | 816 | 234.18 | 581.82 |
| scaffold1359248.1_Zosma56g00900 | 0.44 | 0.11 | 3.97 | 1.18E-16 | 1026 | 288.69 | 737.31 |
| scaffold1361220.1_Zosma48g00240 | 0.52 | 0.16 | 3.17 | 7.97E-05 | 246 | 62.66 | 183.34 |
| scaffold1381012.1_Zosma5g02020 | 0.39 | 0.07 | 5.93 | 3.52E-08 | 372 | 97.09 | 274.91 |
| scaffold1385093.1_Zosma21g00990 | 0.72 | 0.12 | 6.01 | 1.70E-16 | 417 | 143.86 | 273.14 |
| scaffold1385093.1_Zosma21g00990 | 0.70 | 0.17 | 4.09 | 2.91E-12 | 429 | 153.64 | 275.36 |
| scaffold1385093.1_Zosma21g00990 | 0.94 | 0.07 | 12.86 | 1.01E-22 | 441 | 147.35 | 293.65 |
| scaffold1385093.1_Zosma21g00990 | 0.96 | 0.08 | 11.36 | 1.47E-16 | 279 | 106.89 | 172.11 |
| scaffold1390018.1_Zosma52g01130 | 0.61 | 0.03 | 23.62 | 0.0015807 | 84 | 24.11 | 59.89 |
| scaffold1408571.1_Zosma114g00430 | 0.37 | 0.15 | 2.55 | 2.84E-06 | 711 | 185.75 | 525.25 |
| scaffold1411202.1_Zosma74g01010 | 0.82 | 0.20 | 4.15 | 2.21E-11 | 294 | 95.79 | 198.21 |
| scaffold1412345.1_Zosma186g00050 | 0.36 | 0.13 | 2.65 | 2.00E-23 | 2604 | 745.22 | 1858.79 |
| scaffold1413413.1_Zosma93g00140 | 0.31 | 0.08 | 3.92 | 0.0073761 | 135 | 39.39 | 95.61 |
| scaffold1413847.1_Zosma161g00670 | 0.75 | 0.12 | 6.37 | 1.88E-41 | 1155 | 394.00 | 761.00 |
| scaffold1437629.1_Zosma11g00220 | 0.53 | 0.25 | 2.14 | 2.55E-11 | 1275 | 341.23 | 933.77 |
| scaffold1438400.1_Zosma97g00450 | 0.49 | 0.17 | 2.95 | 0.0005914 | 234 | 56.94 | 177.06 |
| scaffold1455213.1_Zosma49g01100 | 0.35 | 0.16 | 2.18 | 5.40E-06 | 705 | 264.09 | 440.91 |
| scaffold1462923.1_Zosma120g00260 | 0.28 | 0.05 | 5.68 | 8.89E-06 | 300 | 107.45 | 192.55 |
| scaffold1476758.1_Zosma93g00140 | 0.31 | 0.08 | 3.92 | 0.0073761 | 135 | 39.39 | 95.61 |
| scaffold1481135.1_Zosma56g00900 | 0.35 | 0.23 | 1.51 | 4.46E-07 | 2931 | 712.80 | 2218.21 |
| scaffold1484297.2_Zosma70g00390 | 0.29 | 0.03 | 8.45 | 1.28E-05 | 336 | 76.97 | 259.04 |
| scaffold1485581.1_Zosma60g00620 | 0.43 | 0.13 | 3.32 | 9.68E-13 | 981 | 236.79 | 744.21 |
| scaffold1488588.1_Zosma142g00200 | 0.42 | 0.14 | 2.90 | 1.62E-09 | 714 | 218.32 | 495.68 |
| scaffold1495367.2_Zosma82g00260 | 0.51 | 0.01 | 50.00 | 8.64E-05 | 135 | 31.07 | 103.93 |
| scaffold1495657.1_Zosma14g00290 | 0.58 | 0.25 | 2.29 | 1.10E-09 | 690 | 234.36 | 455.64 |
| scaffold1497154.1_Zosma78g00200 | 0.95 | 0.34 | 2.80 | 1.88E-05 | 270 | 51.26 | 218.74 |
| scaffold158536.1_Zosma44g01150 | 0.62 | 0.11 | 5.52 | 1.42E-39 | 1308 | 478.12 | 829.88 |
| scaffold173577.1_Zosma386g00080 | 0.39 | 0.14 | 2.74 | 1.10E-11 | 1092 | 304.88 | 787.12 |
| scaffold186151.1_Zosma274g00010 | 0.42 | 0.25 | 1.68 | 0.0014209 | 648 | 174.04 | 473.96 |
| scaffold18783.1_ZosmaC1g00530 | 0.19 | 0.05 | 3.91 | 6.01E-06 | 579 | 209.10 | 369.90 |
| scaffold18783.1_ZosmaCg00610 | 0.19 | 0.05 | 3.91 | 6.01E-06 | 579 | 209.10 | 369.90 |
| scaffold18783.1_ZosmaM1g00660 | 0.20 | 0.03 | 7.79 | 0.0001352 | 288 | 97.59 | 190.41 |
| scaffold256732.1_Zosma23g00970 | 0.35 | 0.11 | 3.15 | 2.21E-14 | 1434 | 357.75 | 1076.25 |
| scaffold273435.1_Zosma49g01020 | 0.20 | 0.00 | 50.00 | 0.0014766 | 180 | 45.73 | 134.27 |
| scaffold329061.1_Zosma19g00820 | 0.66 | 0.03 | 21.92 | 1.23E-12 | 207 | 89.89 | 117.11 |
| scaffold362072.3_Zosma89g00310 | 0.52 | 0.29 | 1.78 | 0.0045162 | 357 | 95.65 | 261.35 |
| scaffold398405.1_Zosma95g00100 | 0.37 | 0.05 | 8.08 | 1.28E-09 | 459 | 119.28 | 339.72 |
| scaffold403838.1_Zosma16g00810 | 0.24 | 0.02 | 10.54 | 0.0061682 | 171 | 37.04 | 133.96 |
| scaffold423412.1_Zosma10g00820 | 0.73 | 0.33 | 2.22 | 4.16E-11 | 1098 | 231.53 | 866.47 |
| scaffold44077.1_Zosma383g00120 | 0.39 | 0.10 | 3.89 | 3.59E-21 | 1350 | 456.60 | 893.40 |
| scaffold446685.1_Zosma53g00740 | 1.04 | 0.26 | 4.03 | 3.45E-07 | 255 | 61.01 | 193.99 |
| scaffold484777.1_Zosma147g00100 | 0.29 | 0.20 | 1.49 | 0.009928 | 927 | 231.67 | 695.33 |
| scaffold485976.1_Zosma166g00640 | 1.10 | 0.66 | 1.68 | 6.63E-08 | 1563 | 349.47 | 1213.53 |
| scaffold485976.1_Zosma166g00640 | 1.10 | 0.57 | 1.92 | 3.91E-14 | 1371 | 889.20 | 481.80 |
| scaffold496848.1_Zosma27g00250 | 0.32 | 0.19 | 1.70 | 0.0068272 | 573 | 159.28 | 413.72 |
| scaffold503484.1_Zosma415g00130 | 0.63 | 0.43 | 1.47 | 0.0016368 | 732 | 190.86 | 541.14 |
| scaffold53059.1_Zosma41g00230 | 0.45 | 0.16 | 2.83 | 4.06E-10 | 816 | 203.04 | 612.96 |
| scaffold53059.1_Zosma47g00010 | 0.92 | 0.15 | 5.96 | 1.46E-14 | 384 | 88.24 | 295.76 |
| scaffold550327.2_Zosma2g03310 | 0.35 | 0.16 | 2.13 | 5.00E-05 | 651 | 177.80 | 473.20 |
| scaffold564832.1_Zosma251g00350 | 0.36 | 0.10 | 3.53 | 1.17E-36 | 2697 | 876.54 | 1820.46 |
| scaffold582580.2_Zosma212g00010 | 0.42 | 0.12 | 3.53 | 3.81E-07 | 504 | 117.84 | 386.16 |
| scaffold582962.1_Zosma222g00150 | 0.47 | 0.15 | 3.11 | 1.68E-05 | 315 | 86.49 | 228.51 |
| scaffold58534.1_Zosma212g00010 | 0.40 | 0.10 | 3.89 | 1.37E-06 | 399 | 101.22 | 297.78 |
| scaffold58534.1_Zosma44g00990 | 0.38 | 0.15 | 2.50 | 0.0002676 | 405 | 105.40 | 299.60 |
| scaffold611248.1_Zosma135g00150 | 0.34 | 0.08 | 4.52 | 2.71E-05 | 300 | 88.00 | 212.00 |
| scaffold624150.1_Zosma12g00830 | 0.32 | 0.10 | 3.20 | 0.0039984 | 210 | 68.34 | 141.66 |
| scaffold635475.1_Zosma93g00140 | 0.35 | 0.07 | 4.94 | 0.0042755 | 135 | 38.48 | 96.52 |
| scaffold685858.1_Zosma49g00800 | 0.34 | 0.05 | 7.33 | 8.70E-05 | 279 | 60.14 | 218.86 |
| scaffold71065.1_Zosma192g00580 | 0.40 | 0.17 | 2.29 | 6.41E-10 | 1413 | 308.89 | 1104.11 |
| scaffold719410.1_Zosma182g00390 | 0.63 | 0.46 | 1.35 | 0.0097641 | 903 | 200.91 | 702.09 |
| scaffold731352.1_Zosma11g00780 | 0.42 | 0.14 | 2.92 | 1.46E-31 | 2652 | 809.66 | 1842.34 |
| scaffold746411.1_Zosma44g01150 | 0.56 | 0.17 | 3.20 | 1.67E-07 | 342 | 130.81 | 211.19 |
| scaffold757470.1_Zosma138g00020 | 0.48 | 0.19 | 2.54 | 2.25E-14 | 1500 | 340.63 | 1159.37 |
| scaffold779396.1_Zosma268g00080 | 0.29 | 0.13 | 2.19 | 0.0054659 | 372 | 107.53 | 264.47 |
| scaffold790805.1_Zosma56g00900 | 0.43 | 0.16 | 2.64 | 3.16E-22 | 2154 | 594.61 | 1559.39 |
| scaffold791666.1_Zosma71g00500 | 0.41 | 0.18 | 2.24 | 2.73E-09 | 1233 | 291.25 | 941.75 |
| scaffold816105.1_Zosma209g00440 | 0.59 | 0.07 | 8.76 | 2.43E-06 | 162 | 48.46 | 113.54 |
| scaffold839026.1_Zosma162g00040 | 0.83 | 0.21 | 4.04 | 1.01E-10 | 321 | 87.09 | 233.91 |
| scaffold84736.1_Zosma27g00420 | 0.41 | 0.18 | 2.30 | 0.0010128 | 327 | 92.84 | 234.17 |
| scaffold854214.2_Zosma11g00220 | 0.35 | 0.07 | 5.15 | 0.000134 | 249 | 61.04 | 187.96 |
| scaffold927015.1_Zosma215g00240 | 0.34 | 0.10 | 3.40 | 0.0005018 | 252 | 77.16 | 174.84 |
| scaffold986544.1_Zosma49g00390 | 0.82 | 0.22 | 3.82 | 6.10E-14 | 477 | 177.87 | 299.14 |
| scaffold986544.1_Zosma49g00390 | 1.04 | 0.18 | 5.72 | 0.0008335 | 318 | 27.48 | 290.52 |
| scaffold997380.1_Zosma238g00010 | 0.45 | 0.07 | 6.26 | 3.42E-18 | 891 | 214.99 | 676.01 |
| scaffold997380.1_Zosma238g00020 | 0.49 | 0.08 | 6.01 | 4.37E-23 | 1062 | 264.92 | 797.08 |

**Table S13.** Statistics of transcription factors and transcriptional regulators.

| Category | Type | *A. konjac* | *S. polyrhiza* | *Z. marina* | *Z. mays** |
| --- | --- | --- | --- | --- | --- |
|  | Alfin-like | 7 | 6 | 6 | 16 |
|  | AP2/ERF-AP2 | 9 | 11 | 14 | 26 |
|  | AP2/ERF-ERF | 123 | 68 | 3 | 194 |
|  | AP2/ERF-RAV | 0 | 2 | 84 | 3 |
|  | B3 | 19 | 21 | 32 | 54 |
| Transcription Factors | B3-ARF | 3 | 16 | 14 | 36 |
|  | BBR-BPC | 11 | 5 | 5 | 4 |
|  | BES1 | 14 | 8 | 6 | 11 |
|  | bHLH | 57 | 82 | 92 | 183 |
|  | BSD | 0 | 1 | 1 | 1 |
|  | bZIP | 46 | 48 | 47 | 121 |
|  | C2C2-YABBY | 9 | 6 | 7 | 13 |
|  | C2C2-Dof | 35 | 26 | 27 | 47 |
|  | C2C2-GATA | 12 | 20 | 24 | 42 |
|  | C2C2-CO-like | 3 | 9 | 5 | 15 |
|  | C2C2-LSD | 2 | 3 | 3 | 6 |
|  | C2H2 | 112 | 69 | 95 | 163 |
|  | C3H | 40 | 46 | 45 | 78 |
|  | CAMTA | 0 | 4 | 5 | 7 |
|  | CPP | 3 | 5 | 3 | 13 |
|  | CSD | 0 | 3 | 2 | 4 |
|  | DBB | 6 | 2 | 2 | 9 |
|  | DBP | 3 | 2 | 2 | 5 |
|  | DDT | 6 | 4 | 4 | 11 |
|  | E2F-DP | 8 | 5 | 7 | 20 |
|  | EIL | 5 | 3 | 4 | 9 |
|  | FAR1 | 0 | 14 | 119 | 19 |
|  | GARP-ARR-B | 3 | 6 | 5 | 7 |
|  | GARP-G2-like | 26 | 30 | 43 | 62 |
|  | GeBP | 5 | 8 | 7 | 28 |
|  | GRAS | 50 | 20 | 26 | 101 |
|  | GRF | 7 | 6 | 10 | 15 |
|  | HB-HD-ZIP | 29 | 18 | 24 | 56 |
|  | HB-WOX | 10 | 12 | 15 | 21 |
|  | HB-PHD | 0 | 2 | 1 | 4 |
|  | HB-BELL | 6 | 8 | 11 | 16 |
|  | HB-KNOX | 3 | 3 | 8 | 13 |
|  | HB-other | 26 | 18 | 19 | 34 |
|  | HRT | 0 | 1 | 1 | 1 |
|  | HSF | 28 | 13 | 12 | 29 |
|  | LFY | 2 | 1 | 1 | 2 |
|  | LIM | 1 | 1 | 4 | 8 |
|  | LOB | 39 | 33 | 25 | 44 |
|  | MADS-MIKC | 4 | 15 | 20 | 43 |
|  | MADS-M-type | 42 | 28 | 30 | 45 |
|  | MYB | 10 | 82 | 94 | 170 |
|  | MYB-related | 91 | 52 | 37 | 141 |
|  | NAC | 61 | 53 | 61 | 135 |
|  | NF-X1 | 2 | 2 | 2 | 4 |
|  | NF-YA | 10 | 5 | 4 | 18 |
|  | NF-YB | 19 | 11 | 24 | 19 |
|  | NF-YC | 11 | 9 | 6 | 16 |
|  | OFP | 31 | 18 | 24 | 43 |
|  | PLATZ | 18 | 13 | 20 | 15 |
|  | RWP-RK | 5 | 12 | 7 | 17 |
|  | S1Fa-like | 3 | 2 | 2 | 2 |
|  | SAP | 1 | 1 | 1 | 0 |
|  | SBP | 30 | 16 | 13 | 37 |
|  | SRS | 1 | 5 | 6 | 12 |
|  | STAT | 1 | 1 | 0 | 1 |
|  | TCP | 22 | 16 | 14 | 46 |
|  | Tify | 14 | 14 | 9 | 35 |
|  | Trihelix | 31 | 25 | 23 | 45 |
|  | TUB | 15 | 9 | 6 | 15 |
|  | ULT | 5 | 2 | 1 | 2 |
|  | VOZ | 1 | 1 | 1 | 6 |
|  | Whirly | 2 | 3 | 2 | 2 |
|  | WRKY | 65 | 43 | 43 | 130 |
|  | zf-HD | 12 | 8 | 9 | 22 |
|  | Total | 1275 | 1115 | 1329 | 2572 |
|  |  |  |  |  |  |
| Transcriptional Regulators | ARID | 3 | 4 | 15 | 11 |
|  | AUX/IAA | 34 | 18 | 15 | 50 |
|  | Coactivator p15 | 1 | 2 | 3 | 3 |
|  | GNAT | 21 | 24 | 27 | 43 |
|  | HMG | 11 | 9 | 12 | 15 |
|  | IWS1 | 8 | 11 | 6 | 25 |
|  | Jumonji | 18 | 10 | 15 | 24 |
|  | LUG | 3 | 3 | 4 | 3 |
|  | MBF1 | 4 | 2 | 1 | 3 |
|  | MED6 | 1 | 1 | 2 | 2 |
|  | MED7 | 2 | 1 | 2 | 2 |
|  | mTERF | 39 | 25 | 28 | 31 |
|  | Others | 53 | 41 | 47 | 106 |
|  | PHD | 27 | 23 | 31 | 44 |
|  | Pseudo ARR-B | 2 | 2 | 2 | 5 |
|  | RB | 1 | 1 | 2 | 5 |
|  | Rcd1-like | 1 | 2 | 2 | 13 |
|  | SET | 27 | 29 | 29 | 52 |
|  | SNF2 | 41 | 28 | 28 | 46 |
|  | SOH1 | 0 | 1 | 3 | 1 |
|  | SWI/SNF-BAF60b | 15 | 11 | 4 | 29 |
|  | SWI/SNF-SWI3 | 6 | 3 | 9 | 4 |
|  | TAZ | 4 | 4 | 4 | 6 |
|  | TRAF | 23 | 16 | 16 | 50 |
|  | Total | 345 | 271 | 307 | 573 |

* Download from iTAK website.

**Table S14.** The genes in starch and sucrose metabolism pathways.

| Gene | KO | Gene Name | KEGG Annotation |
| --- | --- | --- | --- |
| scaffold1011362.1 | K00688 | PYG | glycogen phosphorylase [EC:2.4.1.1] |
| scaffold46524.1 | K00688 | PYG | glycogen phosphorylase [EC:2.4.1.1] |
| scaffold920315.1 | K00688 | PYG | glycogen phosphorylase [EC:2.4.1.1] |
| scaffold1278416.1 | K00695 | SUS | sucrose synthase [EC:2.4.1.13] |
| scaffold1342607.1 | K00695 | SUS | sucrose synthase [EC:2.4.1.13] |
| scaffold1355376.1 | K00695 | SUS | sucrose synthase [EC:2.4.1.13] |
| scaffold1423096.1 | K00695 | SUS | sucrose synthase [EC:2.4.1.13] |
| scaffold1493037.1 | K00695 | SUS | sucrose synthase [EC:2.4.1.13] |
| scaffold212982.2 | K00695 | SUS | sucrose synthase [EC:2.4.1.13] |
| scaffold560528.1 | K00695 | SUS | sucrose synthase [EC:2.4.1.13] |
| scaffold847042.1 | K00695 | SUS | sucrose synthase [EC:2.4.1.13] |
| scaffold1020641.1 | K00696 | SPS | sucrose-phosphate synthase [EC:2.4.1.14] |
| scaffold133940.1 | K00700 | GBE1 | 1,4-alpha-glucan branching enzyme [EC:2.4.1.18] |
| scaffold1203951.1 | K00703 | glgA | starch synthase [EC:2.4.1.21] |
| scaffold332174.1 | K00703 | glgA | starch synthase [EC:2.4.1.21] |
| scaffold525882.1 | K00703 | glgA | starch synthase [EC:2.4.1.21] |
| scaffold1133930.1 | K00705 | malQ | 4-alpha-glucanotransferase [EC:2.4.1.25] |
| scaffold933272.1 | K00705 | malQ | 4-alpha-glucanotransferase [EC:2.4.1.25] |
| scaffold1455609.1 | K00844 | HK | hexokinase [EC:2.7.1.1] |
| scaffold330628.1 | K00844 | HK | hexokinase [EC:2.7.1.1] |
| scaffold629429.1 | K00844 | HK | hexokinase [EC:2.7.1.1] |
| scaffold941714.1 | K00844 | HK | hexokinase [EC:2.7.1.1] |
| scaffold1284860.1 | K00847 | FRK | fructokinase [EC:2.7.1.4] |
| scaffold1284860.2 | K00847 | FRK | fructokinase [EC:2.7.1.4] |
| scaffold246979.1 | K00847 | FRK | fructokinase [EC:2.7.1.4] |
| scaffold559784.2 | K00847 | FRK | fructokinase [EC:2.7.1.4] |
| scaffold419929.2 | K00975 | glgC | glucose-1-phosphate adenylyltransferase [EC:2.7.7.27] |
| scaffold751375.1 | K00975 | glgC | glucose-1-phosphate adenylyltransferase [EC:2.7.7.27] |
| scaffold883241.1 | K00975 | glgC | glucose-1-phosphate adenylyltransferase [EC:2.7.7.27] |
| scaffold891403.2 | K00975 | glgC | glucose-1-phosphate adenylyltransferase [EC:2.7.7.27] |
| scaffold891403.1 | K00975 | glgC | glucose-1-phosphate adenylyltransferase [EC:2.7.7.27] |
| C427414358.1 | K01087 | otsB | trehalose 6-phosphate phosphatase [EC:3.1.3.12] |
| scaffold1129441.1 | K01087 | otsB | trehalose 6-phosphate phosphatase [EC:3.1.3.12] |
| scaffold49541.1 | K01087 | otsB | trehalose 6-phosphate phosphatase [EC:3.1.3.12] |
| scaffold1095376.1 | K01176 | AMY | alpha-amylase [EC:3.2.1.1] |
| scaffold128328.1 | K01176 | AMY | alpha-amylase [EC:3.2.1.1] |
| scaffold1498724.1 | K01176 | AMY | alpha-amylase [EC:3.2.1.1] |
| scaffold337874.1 | K01176 | AMY | alpha-amylase [EC:3.2.1.1] |
| C427262504.1 | K01177 | BMY | beta-amylase [EC:3.2.1.2] |
| scaffold1074575.1 | K01177 | BMY | beta-amylase [EC:3.2.1.2] |
| scaffold1085831.1 | K01177 | BMY | beta-amylase [EC:3.2.1.2] |
| scaffold1149294.1 | K01177 | BMY | beta-amylase [EC:3.2.1.2] |
| scaffold1259038.1 | K01177 | BMY | beta-amylase [EC:3.2.1.2] |
| scaffold1367378.1 | K01177 | BMY | beta-amylase [EC:3.2.1.2] |
| scaffold1397691.2 | K01177 | BMY | beta-amylase [EC:3.2.1.2] |
| scaffold560761.2 | K01177 | BMY | beta-amylase [EC:3.2.1.2] |
| scaffold918331.1 | K01177 | BMY | beta-amylase [EC:3.2.1.2] |
| C426897363.1 | K01179 | EG | endoglucanase [EC:3.2.1.4] |
| C427268988.1 | K01179 | EG | endoglucanase [EC:3.2.1.4] |
| C427384062.1 | K01179 | EG | endoglucanase [EC:3.2.1.4] |
| C427411478.1 | K01179 | EG | endoglucanase [EC:3.2.1.4] |
| scaffold1079274.1 | K01179 | EG | endoglucanase [EC:3.2.1.4] |
| scaffold1260868.1 | K01179 | EG | endoglucanase [EC:3.2.1.4] |
| scaffold1304978.1 | K01179 | EG | endoglucanase [EC:3.2.1.4] |
| scaffold1353699.1 | K01179 | EG | endoglucanase [EC:3.2.1.4] |
| scaffold1374251.1 | K01179 | EG | endoglucanase [EC:3.2.1.4] |
| scaffold1397164.1 | K01179 | EG | endoglucanase [EC:3.2.1.4] |
| scaffold1397165.1 | K01179 | EG | endoglucanase [EC:3.2.1.4] |
| scaffold1404811.2 | K01179 | EG | endoglucanase [EC:3.2.1.4] |
| scaffold1404811.1 | K01179 | EG | endoglucanase [EC:3.2.1.4] |
| scaffold179116.1 | K01179 | EG | endoglucanase [EC:3.2.1.4] |
| scaffold401989.1 | K01179 | EG | endoglucanase [EC:3.2.1.4] |
| scaffold723.1 | K01179 | EG | endoglucanase [EC:3.2.1.4] |
| scaffold918006.1 | K01179 | EG | endoglucanase [EC:3.2.1.4] |
| scaffold978829.1 | K01179 | EG | endoglucanase [EC:3.2.1.4] |
| C427388950.1 | K01187 | malZ | alpha-glucosidase [EC:3.2.1.20] |
| scaffold1362775.1 | K01187 | malZ | alpha-glucosidase [EC:3.2.1.20] |
| scaffold570677.1 | K01187 | malZ | alpha-glucosidase [EC:3.2.1.20] |
| scaffold1031791.2 | K01188 | bglU | beta-glucosidase [EC:3.2.1.21] |
| scaffold1086995.2 | K01188 | bglU | beta-glucosidase [EC:3.2.1.21] |
| scaffold1244523.1 | K01188 | bglU | beta-glucosidase [EC:3.2.1.21] |
| scaffold1276856.1 | K01188 | bglU | beta-glucosidase [EC:3.2.1.21] |
| scaffold1386660.1 | K01188 | bglU | beta-glucosidase [EC:3.2.1.21] |
| scaffold1495048.1 | K01188 | bglU | beta-glucosidase [EC:3.2.1.21] |
| scaffold353795.1 | K01188 | bglU | beta-glucosidase [EC:3.2.1.21] |
| scaffold455866.1 | K01188 | bglU | beta-glucosidase [EC:3.2.1.21] |
| scaffold634396.1 | K01188 | bglU | beta-glucosidase [EC:3.2.1.21] |
| scaffold81774.1 | K01188 | bglU | beta-glucosidase [EC:3.2.1.21] |
| scaffold852074.1 | K01188 | bglU | beta-glucosidase [EC:3.2.1.21] |
| scaffold928709.1 | K01188 | bglU | beta-glucosidase [EC:3.2.1.21] |
| scaffold971433.1 | K01188 | bglU | beta-glucosidase [EC:3.2.1.21] |
| C427311376.1 | K01193 | INV | beta-fructofuranosidase [EC:3.2.1.26] |
| scaffold1123482.1 | K01193 | INV | beta-fructofuranosidase [EC:3.2.1.26] |
| scaffold1384031.1 | K01193 | INV | beta-fructofuranosidase [EC:3.2.1.26] |
| scaffold1502160.1 | K01193 | INV | beta-fructofuranosidase [EC:3.2.1.26] |
| scaffold828466.1 | K01193 | INV | beta-fructofuranosidase [EC:3.2.1.26] |
| scaffold963093.1 | K01193 | INV | beta-fructofuranosidase [EC:3.2.1.26] |
| scaffold1330052.1 | K01513 | ENPP1_3 | ectonucleotide pyrophosphatase/phosphodiesterase family member 1/3 [EC:3.1.4.1 3.6.1.9] |
| scaffold1428136.1 | K01513 | ENPP1_3 | ectonucleotide pyrophosphatase/phosphodiesterase family member 1/3 [EC:3.1.4.1 3.6.1.9] |
| scaffold191259.1 | K01513 | ENPP1_3 | ectonucleotide pyrophosphatase/phosphodiesterase family member 1/3 [EC:3.1.4.1 3.6.1.9] |
| scaffold556302.1 | K01513 | ENPP1_3 | ectonucleotide pyrophosphatase/phosphodiesterase family member 1/3 [EC:3.1.4.1 3.6.1.9] |
| scaffold825807.2 | K01513 | ENPP1_3 | ectonucleotide pyrophosphatase/phosphodiesterase family member 1/3 [EC:3.1.4.1 3.6.1.9] |
| scaffold1166607.1 | K01810 | GPI | glucose-6-phosphate isomerase [EC:5.3.1.9] |
| scaffold1348777.1 | K01810 | GPI | glucose-6-phosphate isomerase [EC:5.3.1.9] |
| scaffold140436.1 | K01835 | pgm | phosphoglucomutase [EC:5.4.2.2] |
| scaffold464599.1 | K01835 | pgm | phosphoglucomutase [EC:5.4.2.2] |
| C427412298.1 | K05349 | bglX | beta-glucosidase [EC:3.2.1.21] |
| scaffold1011039.1 | K05349 | bglX | beta-glucosidase [EC:3.2.1.21] |
| scaffold1304829.1 | K05349 | bglX | beta-glucosidase [EC:3.2.1.21] |
| scaffold1351191.1 | K05349 | bglX | beta-glucosidase [EC:3.2.1.21] |
| scaffold208556.2 | K05349 | bglX | beta-glucosidase [EC:3.2.1.21] |
| scaffold758530.2 | K05349 | bglX | beta-glucosidase [EC:3.2.1.21] |
| scaffold774767.1 | K05349 | bglX | beta-glucosidase [EC:3.2.1.21] |
| scaffold972373.2 | K05349 | bglX | beta-glucosidase [EC:3.2.1.21] |
| scaffold230252.2 | K05350 | bglB | beta-glucosidase [EC:3.2.1.21] |
| scaffold1407631.1 | K07024 | SPP | sucrose-6-phosphatase [EC:3.1.3.24] |
| scaffold360137.1 | K07024 | SPP | sucrose-6-phosphatase [EC:3.1.3.24] |
| scaffold835830.1 | K13679 | WAXY | granule-bound starch synthase [EC:2.4.1.242] |
| C426932587.1 | K16055 | TPS | trehalose 6-phosphate synthase/phosphatase [EC:2.4.1.15 3.1.3.12] |
| C427324402.1 | K16055 | TPS | trehalose 6-phosphate synthase/phosphatase [EC:2.4.1.15 3.1.3.12] |
| scaffold1279758.2 | K16055 | TPS | trehalose 6-phosphate synthase/phosphatase [EC:2.4.1.15 3.1.3.12] |
| scaffold1279758.1 | K16055 | TPS | trehalose 6-phosphate synthase/phosphatase [EC:2.4.1.15 3.1.3.12] |
| scaffold1280205.1 | K16055 | TPS | trehalose 6-phosphate synthase/phosphatase [EC:2.4.1.15 3.1.3.12] |
| scaffold1284582.1 | K16055 | TPS | trehalose 6-phosphate synthase/phosphatase [EC:2.4.1.15 3.1.3.12] |
| scaffold1317247.1 | K16055 | TPS | trehalose 6-phosphate synthase/phosphatase [EC:2.4.1.15 3.1.3.12] |
| scaffold1367382.1 | K16055 | TPS | trehalose 6-phosphate synthase/phosphatase [EC:2.4.1.15 3.1.3.12] |
| scaffold1398652.1 | K16055 | TPS | trehalose 6-phosphate synthase/phosphatase [EC:2.4.1.15 3.1.3.12] |
| scaffold1407958.1 | K16055 | TPS | trehalose 6-phosphate synthase/phosphatase [EC:2.4.1.15 3.1.3.12] |
| scaffold1442517.2 | K16055 | TPS | trehalose 6-phosphate synthase/phosphatase [EC:2.4.1.15 3.1.3.12] |
| scaffold1484606.1 | K16055 | TPS | trehalose 6-phosphate synthase/phosphatase [EC:2.4.1.15 3.1.3.12] |
| scaffold373430.1 | K16055 | TPS | trehalose 6-phosphate synthase/phosphatase [EC:2.4.1.15 3.1.3.12] |
| scaffold829463.1 | K16055 | TPS | trehalose 6-phosphate synthase/phosphatase [EC:2.4.1.15 3.1.3.12] |
| scaffold862882.2 | K16055 | TPS | trehalose 6-phosphate synthase/phosphatase [EC:2.4.1.15 3.1.3.12] |
| scaffold958601.2 | K16055 | TPS | trehalose 6-phosphate synthase/phosphatase [EC:2.4.1.15 3.1.3.12] |
| scaffold1178421.1 | K19891 | GN1_2_3 | glucan endo-1,3-beta-glucosidase 1/2/3 [EC:3.2.1.39] |
| scaffold1187751.1 | K19891 | GN1_2_3 | glucan endo-1,3-beta-glucosidase 1/2/3 [EC:3.2.1.39] |
| scaffold286621.1 | K19891 | GN1_2_3 | glucan endo-1,3-beta-glucosidase 1/2/3 [EC:3.2.1.39] |
| scaffold1103166.1 | K19892 | GN4 | glucan endo-1,3-beta-glucosidase 4 [EC:3.2.1.39] |
| scaffold206958.1 | K19892 | GN4 | glucan endo-1,3-beta-glucosidase 4 [EC:3.2.1.39] |
| C426843033.1 | K19893 | GN5_6 | glucan endo-1,3-beta-glucosidase 5/6 [EC:3.2.1.39] |
| scaffold1319419.1 | K19893 | GN5_6 | glucan endo-1,3-beta-glucosidase 5/6 [EC:3.2.1.39] |
| scaffold1383924.1 | K19893 | GN5_6 | glucan endo-1,3-beta-glucosidase 5/6 [EC:3.2.1.39] |
| scaffold669092.1 | K19893 | GN5_6 | glucan endo-1,3-beta-glucosidase 5/6 [EC:3.2.1.39] |
| scaffold726708.1 | K19893 | GN5_6 | glucan endo-1,3-beta-glucosidase 5/6 [EC:3.2.1.39] |
