# Supplementary material for: Characteristics of Amorphophallus konjac as indicated by its genome
Source: Sci Rep. 2023 Dec 19;13:22684. doi: 10.1038/s41598-023-49963-9 (PMC10730839; doi:10.1038/s41598-023-49963-9)
Supplement: Supplementary file 2 — Supplementary Figures. [file 41598_2023_49963_MOESM2_ESM.docx]

Supplementary Figures


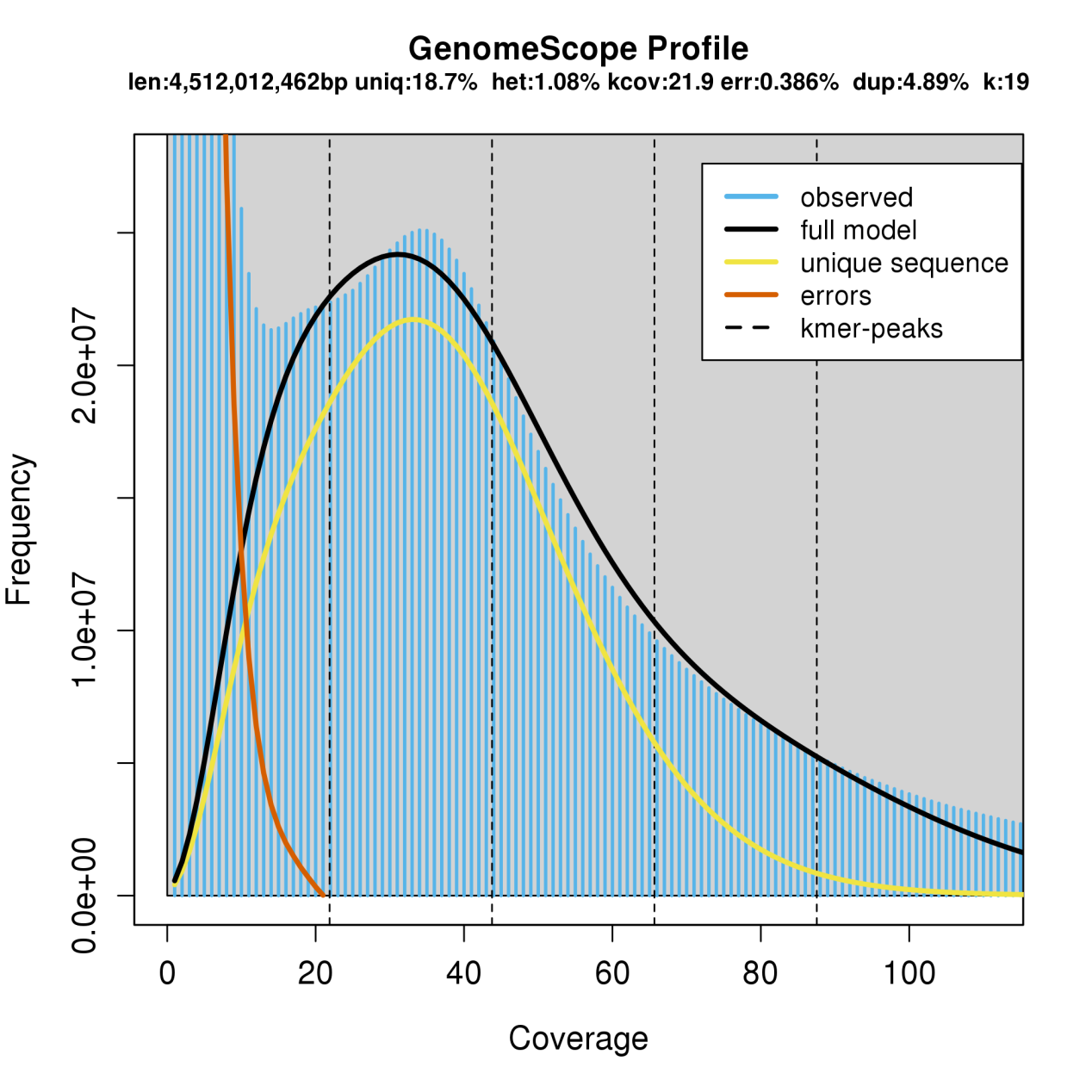


**Fig. S1.** Frequency distribution of the 19-mer graph.

**Fig. S2.** The estimated genome size of *A. konjac* by flow cytometry.


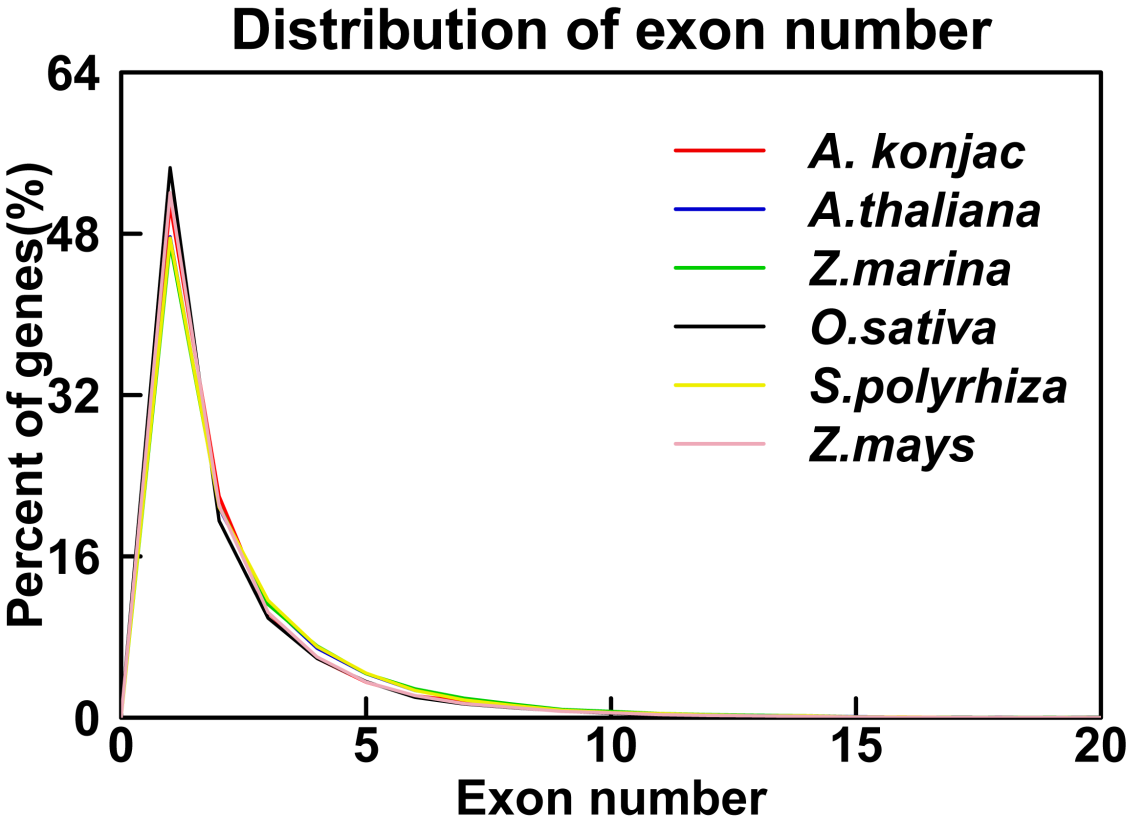


**Fig. S3.** The distribution of exon numbers in annotated mRNA. Annotation data of *A. thaliana, O. sativa, Z. mays, Z. marina, S. polyrhiza* were used in parallel with *A. konjac* annotation data.


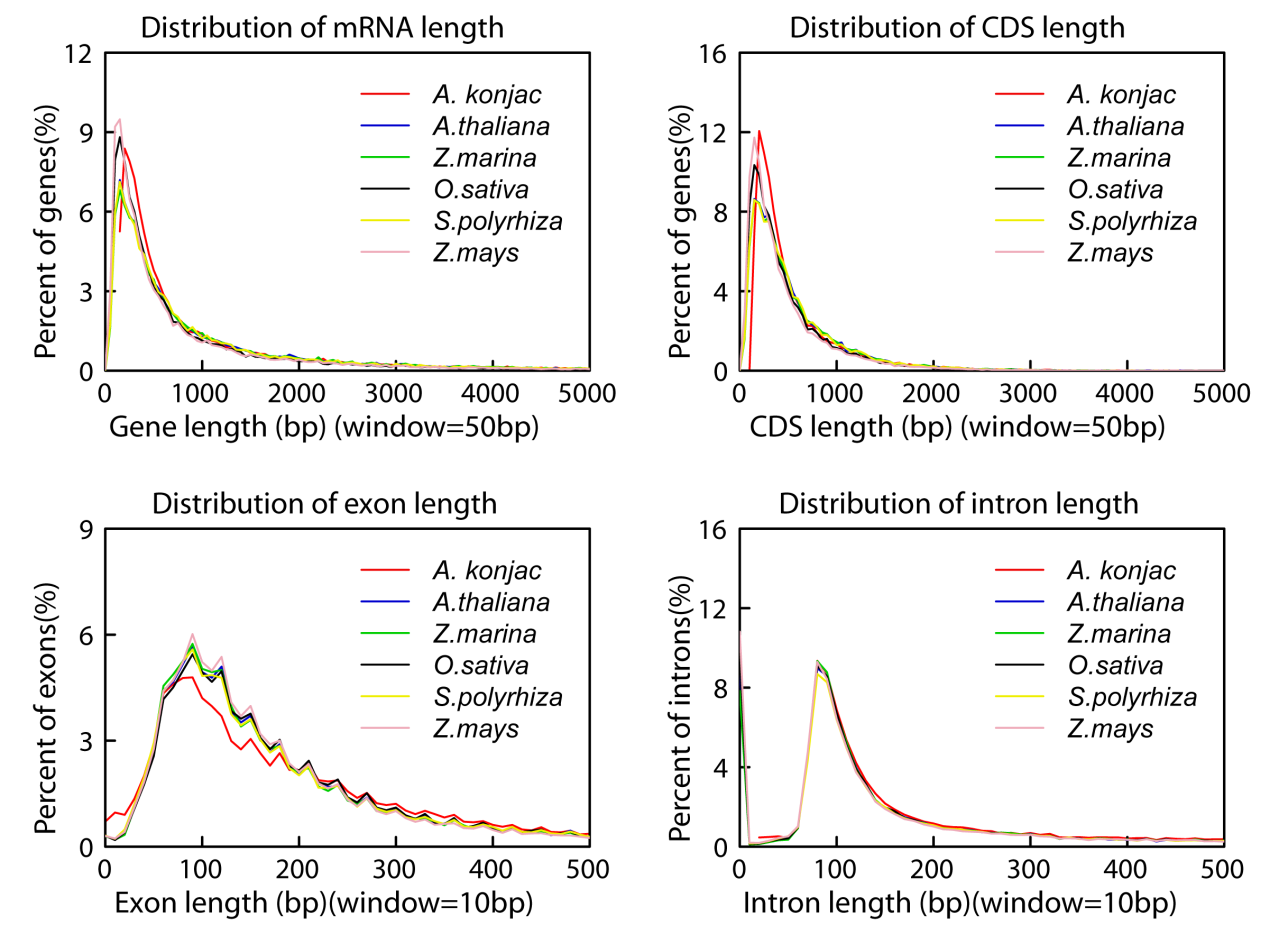


**Fig. S4.** The distribution of mRNA length, CDS length, exon length and exon length in *A. konjac* compared with *A. thaliana, O. sativa, Z. mays, Z. marina,* *S. polyrhiza*.

**Fig. S5.** Venn diagram and GO enrichment analysis. A:Venn diagram showing the number of unique and shared gene families among *A. konjac, O. sativa, Z. mays, Z. marina* and *S. polyrhiza*. B: GO enrichment for unique genes in *A. konjac.*


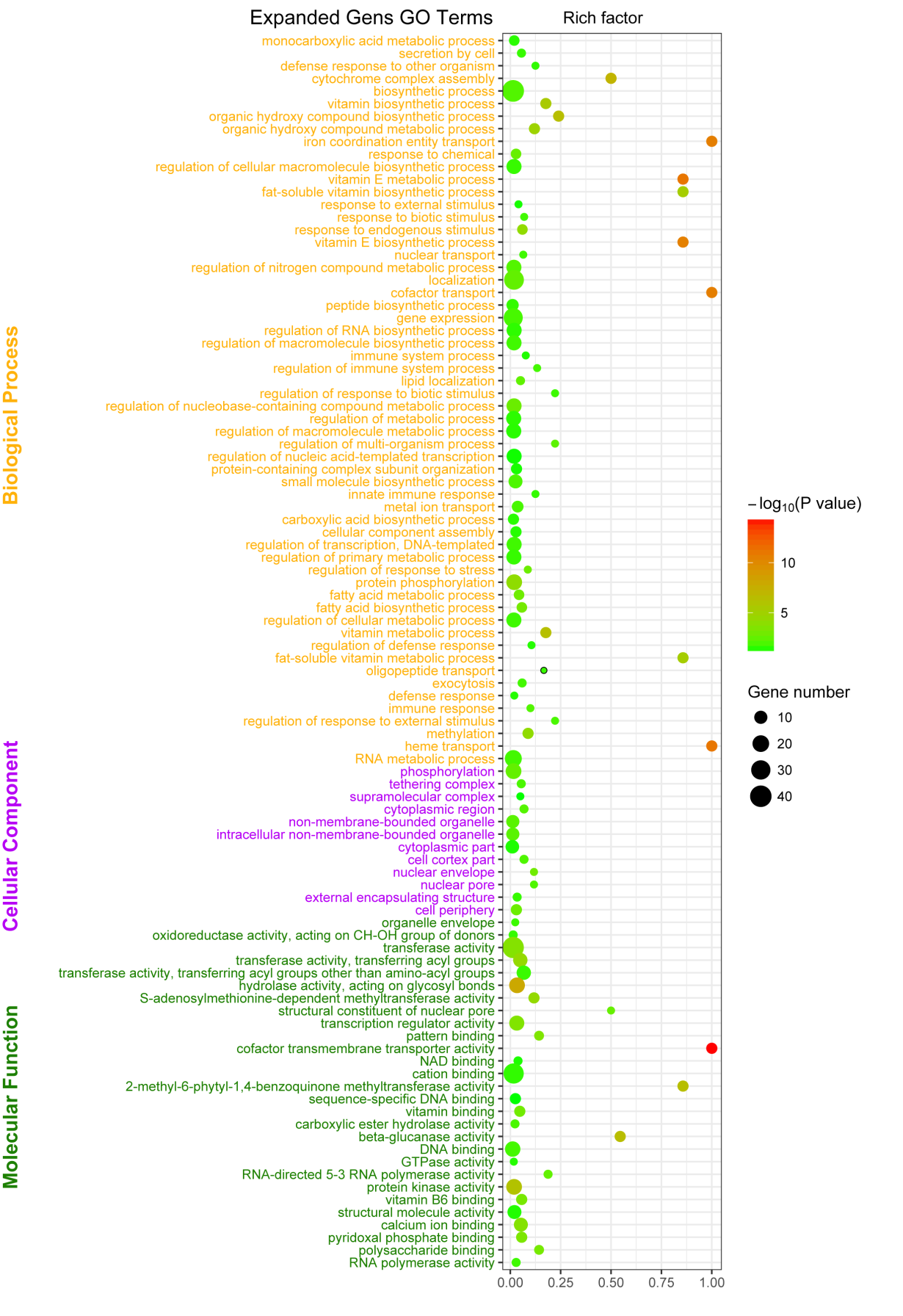


**Fig. S6.** GO enrichment for expanded genes in *A. konjac.*

**Fig. S7.** GO enrichment for contracted genes in *A. konjac.*


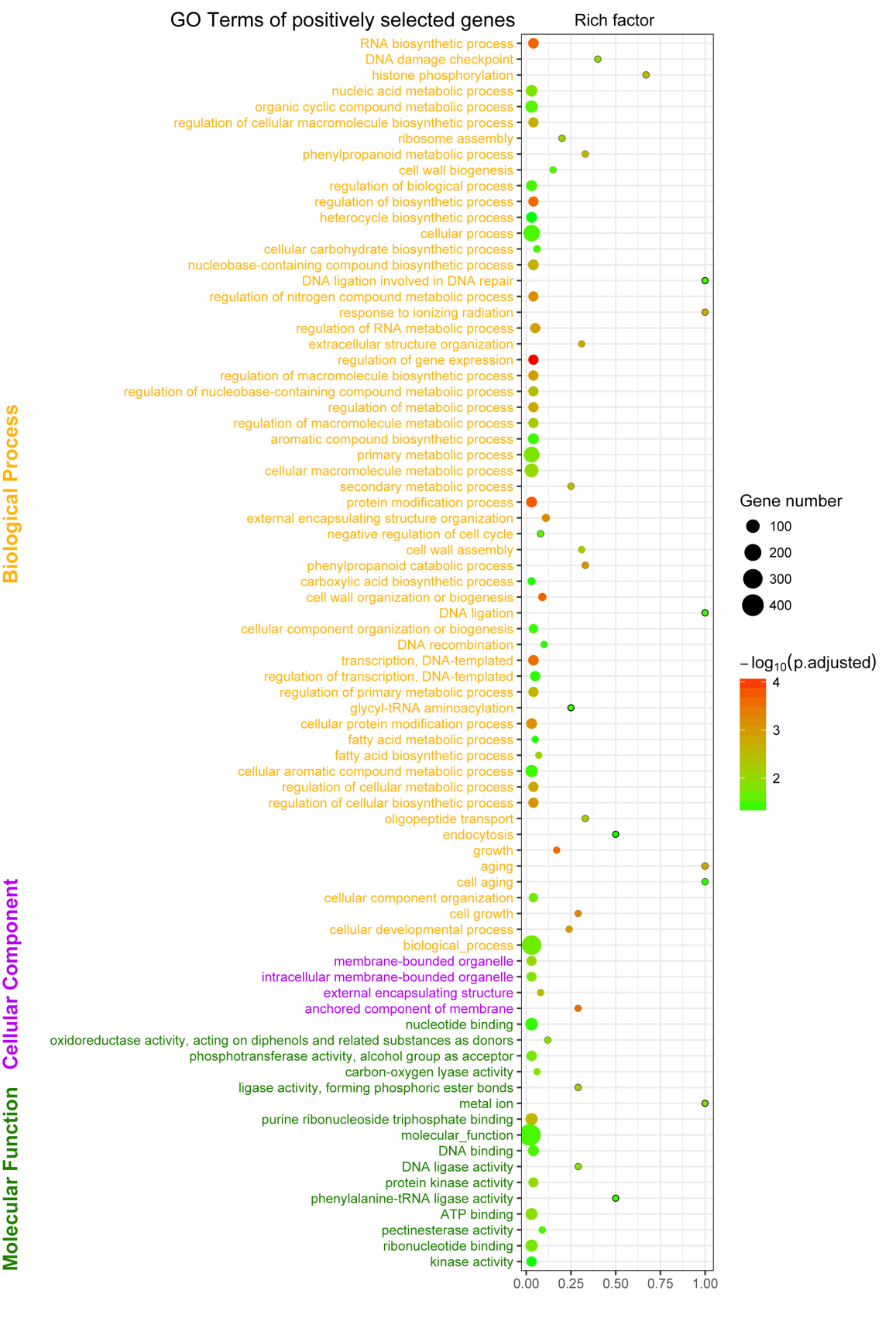


**Fig. S8.** GO enrichment for positively selected genes in *A. konjac* against *S. polyrhiza.*


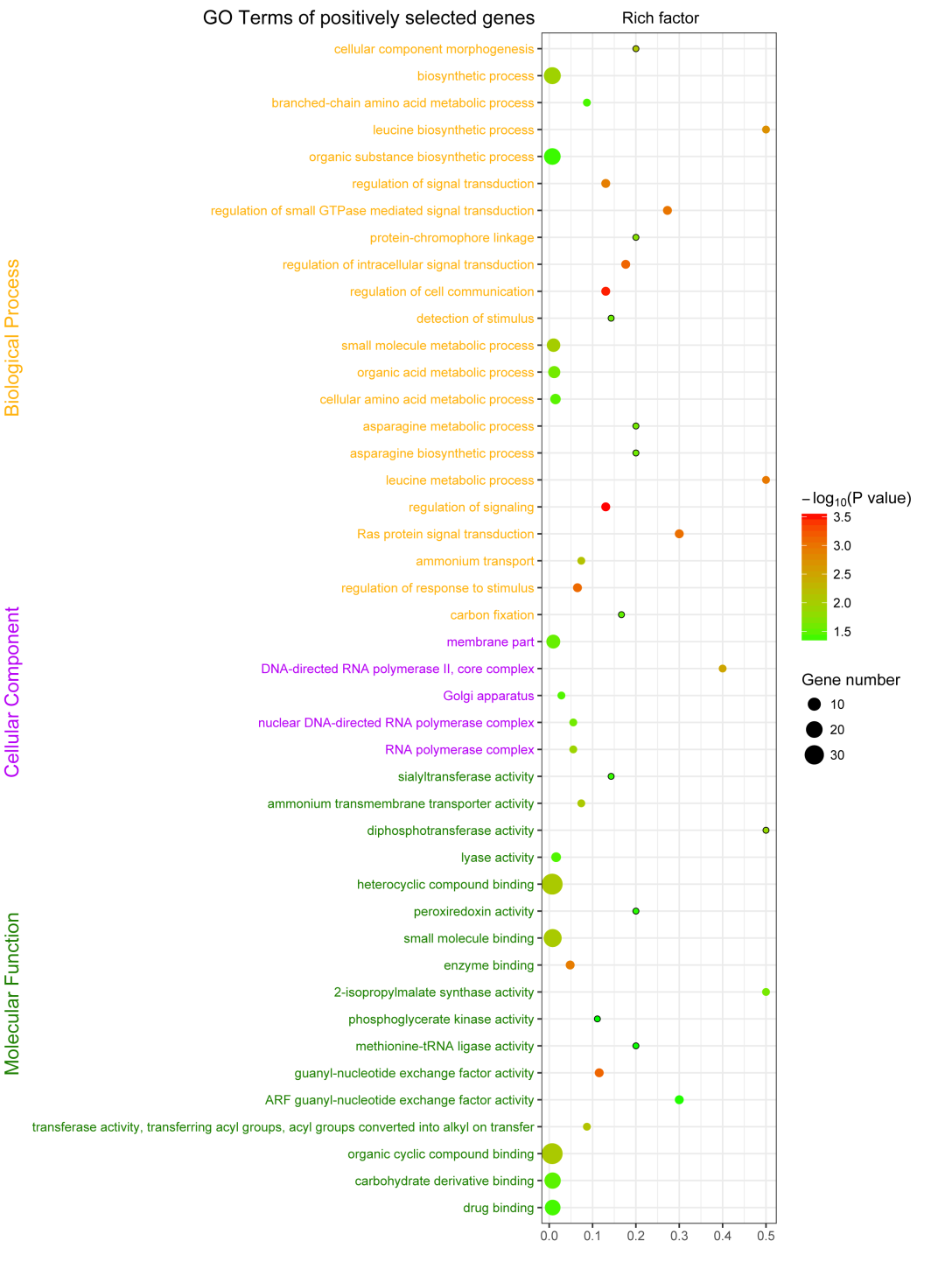


**Fig. S9.** GO enrichment for positively selected genes in *A. konjac* against *Z. marina.*
